# Narrative Review of Emergency Medicine Clinical Research Examining Exclusion by Language

**DOI:** 10.5811/westjem.46547

**Published:** 2025-09-25

**Authors:** Alexa M. Curt, Olivia Kahn-Boesel, Melis Lydston, Melissa A. Meeker, Margaret E. Samuels-Kalow

**Affiliations:** † Massachusetts General Hospital, Department of Emergency Medicine, Boston, Massachusetts; ‡ Massachusetts General Hospital, Department of Medicine, Boston, Massachusetts; § Massachusetts General Hospital, Treadwell Virtual Library, Boston, Massachusetts

## Abstract

**Introduction:**

Over 20% of the United States population speaks a language other than English, and many use the emergency department (ED) to access healthcare. However, there remains concern that patients preferring languages other than English are under-represented in clinical research. Thus, our goal was to assess the proportion of ED studies that excluded patients for recruitment due to language.

**Methods:**

We conducted a narrative review using seven search engines for 2018–2023. We included studies if they mentioned language of participants and prospectively enrolled patients in an ED or prehospital setting. We excluded studies if they only included patients <18 years and/or were conducted exclusively outside the US. Two independent reviewers reviewed studies. Analyses included descriptive statistics.

**Results:**

Of the 10,513 studies we identified, 281 were eligible for review; 163 (58%) excluded non-English language preferred (NELP) patients. Among the 107 interventional studies, 69% excluded NELP patients. Of the 135 studies focused on health equity/social emergency medicine, 47% excluded NELP patients.

**Conclusion:**

We found 163 (58%) studies conducted in the ED that mention language and excluded NELP patients. Additional work is needed to encourage and support inclusive study designs.

## INTRODUCTION

The United States is increasingly multilingual, with an estimated 22% of the US population speaking languages other than English according to the 2020 US Census,^1^ which is a 52% increase from 2000.^1^ These are likely underestimates given their fears centered on immigration status and inability to complete the census form.^2^ Despite the growing percentage of the general population preferring languages other than English, this population remains significantly under-represented in clinical research,^3^ with ongoing disparities in health outcomes compared to English-speaking patients.^4^–^6^ This disparate participation in clinical research limits the ability of the work to inform policy to improve care and equity.

The emergency department (ED) serves a significant proportion of racial and ethnic minority patients^7^ and thus has the potential to be a key location within the healthcare system to mitigate disparities in clinical research recruitment. Despite this, enrollment and consent for clinical studies is more likely to occur in middle-aged White patients.^8^ Many variables could influence this disparate rate of participation, such as mistrust of the healthcare system, fear of additional documentation, or lack of compensation for time.^9^ Although there is likely a complicated web of variables interacting to drive this disparity, studies that explicitly limit recruitment and enrollment to patients who speak English form a critical bottleneck to increasing racial and ethnic minority participation in clinical research.

A recent study showed that 65% of clinical trials conducted in the pediatric population enrolled only English-speaking patients.^10^ However, it is not known how often clinical studies conducted in adult EDs exclude non-English language preferred (NELP) patients from being approached for clinical research studies. Thus, we conducted a narrative review to investigate the number of published or described studies that prospectively enrolled patients in the ED or prehospital setting and reported language of enrollment but excluded NELP patients.

## METHODS

Our review adhered to the Scale for the Assessment of Narrative Review Articles (SANRA) guidelines for narrative reviews.^11^ In March 2023, a medical librarian conducted electronic searches for published literature using Ovid MEDLINE, PubMed Central, Embase.com, Web of Science Core Collection, Cochrane Central Register of Controlled Trials via Ovid, and ClinicalTrials.gov. The search strategy incorporated controlled vocabulary and free-text synonyms for the concepts of emergency care, language limitations, and methods. The full database search strategies are documented in Appendix Supplemental Tables 1–6. We placed a date limit from 2018–2023. The literature search pulled studies within this time frame that occurred in the ED and mentioned language in any capacity. We used a variety of search terms to encompass language for our search, as listed in Supplemental Tables 1–6.

**Figure 1:**
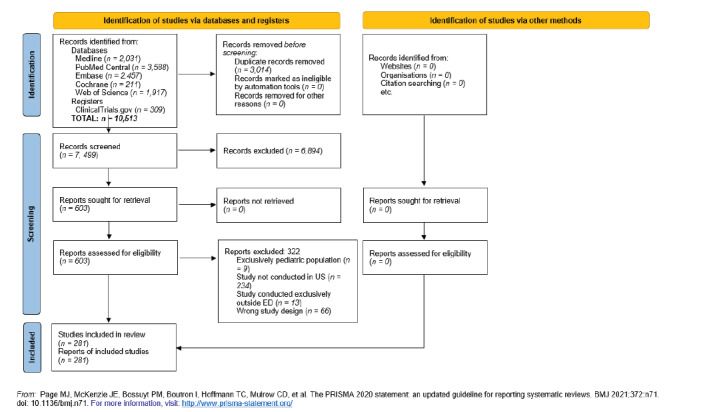
PRISMA* flow diagram demonstrating study evaluation for emergency medicine clinical research examining exclusion by language. *PRISMA, Preferred Reporting Items for Systematic Reviews and Meta-Analyses; *US*, United States; *ED*, emergency department.

Studies were reviewed independently by two reviewers. We resolved discrepancies between reviewers with discussion and consensus. We included studies that approached patients for research consent for any type of clinical research conducted in an ED or in the prehospital setting with emergency medicine services personnel. We also included studies that enrolled patients in additional settings beyond the ED and prehospital setting if they contained an ED or prehospital component. We excluded studies with study populations composed solely of patients < 18 years, although we included studies with populations < 18 years of age if they also enrolled patients > 18. We excluded studies conducted exclusively outside the US. The Preferred Reporting Items for Systematic Reviews and Meta-Analyses (PRISMA) flow diagram reflecting this process is demonstrated in Figure 1.

We combined and de-duplicated all identified studies ed in a single reference manager (EndNote12). We then uploaded the citations into Covidence systematic review software (Veritas Health Innovation Ltd, Melbourne, Australia). Extraction variables are listed in Supplemental Table 7 and include the following: title; year published; authors; journal; journal publication or conference presentation; abstract or manuscript; study design; objective of study (one line describing goal of study); topic of study (health equity/social emergency medicine vs other); primary enrollment or secondary analysis; presence of an intervention; languages recruited (if described); other inclusion criteria for the study; and exclusion criteria. We defined health equity/social emergency medicine as factors related to social determinants of health, social risk, and social needs.^13^

**Population Health Research Capsule**

What do we already know about this issue?

*Non-english language preferred (NELP) patients are frequently under-represented in clinical research, limiting equitable evidence and policy development*.

What was the research question?

*Of studies published in emergency medicine journals between 2018–2023, how many excluded NELP patients?*

What was the major finding of the study?

*Of 281 studies, 58% excluded NELP patients. Among interventional studies, 69% excluded NELP patients*.

How does this improve population health?

*This study highlights the need for more inclusive study designs with support for diverse research populations to better reflect and serve patient populations*.

We ascertained the primary outcome (language of inclusion) by reading the study methods and demographic tables (if included) of the abstracts or manuscripts. We used the inclusion and exclusion criteria on ClinicalTrials.gov to determine whether the clinical trials were excluding patients based on language. We categorized studies as “English only,” “not English only,” and “did not specify” based on the languages of patients recruited for the s-tudy. We then completed descriptive analyses stratified by language recruitment.

## RESULTS

As indicated in the PRISMA flow diagram (Figure 1), a total of 10,513 studies were identified. After removing duplicates, 7,499 abstracts were screened for inclusion criteria by two reviewers (AC, OKB). Next, 603 studies underwent full text review. Finally, 281 studies were extracted as demonstrated in Supplemental Table 7. As shown in Table 1, 163 (58%) of the 281 studies excluded NELP patients and eight (3%) did not specify language of patients recruited. Regarding topic of study, 135 (47%) investigated a research question related to health equity or social emergency medicine. Within these 135 studies, 63 (47%) excluded NELP patients, 70 (52%) included NELP patients, and two (1%) did not specify. The remaining 146 studies covered any other topic of clinical research, of which 100 (68%) excluded NELP patients, 40 (27%) included NELP patients, and six (4%) did not specify. Of the 107 studies (38%) that included an intervention, 74 (69%) excluded NELP patients, 29 (27%) included NELP patients, and four (4%) did not specify languages recruited. For the 174 non-interventional studies, 89 (51%) excluded NELP patients, 81 (47%) included NELP patients, and four (2%) did not specify.

Of the 268 (95%) studies conducted exclusively in an ED or prehospital setting, 157 (59%) excluded NELP patients, 105 (39%) included NELP patients, and six (2%) did not specify. For the remaining 13 studies conducted in additional clinical settings, six (46%) excluded NELP patients, five (38%) included NELP patients, and two (15%) did not specify. Of the 231 studies (82%) that performed analyses on primary enrollment data, 134 (58%) excluded NELP patients, 89 (39%) included NELP patients, and eight (3%) did not specify. For the 50 (18%) studies that performed secondary analyses on data, 29 (58%) datasets excluded NELP patients and 21 (42%) included NELP patients. Within the 50 (18%) abstract publications, 25 (50%) excluded NELP patients, 23 (46%) included NELP patients, and two (4%) did not specify.

For the remaining 231 (82%) papers published, 138 (60%), excluded NELP patients, 87 (38%) included NELP patients, and six (3%) did not specify. Twenty-four (8%) studies included patients < 18 years of age, of which 14 (58%) excluded NELP patients, nine (38%) included NELP patients, and one (4%) did not specify. Of the 244 (86%) that did not include patients < 18 years of age, 145 (59%) excluded NELP patients, 93 (38%) included NELP patients, and six (2%) did not specify. Thirteen studies did not define what ages were recruited; within this group, four (31%) excluded NELP patients, eight (62%) included NELP patients, and one (8%) did not specify.

## DISCUSSION

In this narrative review of prospective ED studies from 2018–2023 that mention language, we found that 163 (58%) studies excluded NELP patients. Among interventional studies, 74 (69%) excluded NELP patients, despite an increasing number of people in the United States who do not speak English.^1^ Interventional studies often have more stringent regulations regarding consent and follow-up, and interpretation of these well-intended regulations may have the unintended consequence of excluding NELP patients. Translating study materials, performing ethical consent, and creating follow-up infrastructure in a non-English language requires significant funds and time. Researchers should be encouraged to request this funding in grants, and institutions should invest in the infrastructure to support multilingual enrollment. This is particularly important with the increasing diversity of the US population to ensure that data driving important policy changes and practice norms are representative.

Among those studies that focused on heath equity/social emergency medicine, it is still worth noting that 47% excluded NELP patients. For this topic in particular, including NELP patients is imperative given the differential rates of social determinants of health experienced by patients who identify as racial and ethnic minorities in the US.^4^–^6^

Additional work is needed to determine how to optimize infrastructure for the ethical inclusion of NELP patients in clinical research. This will likely require a multifaceted effort. Regulatory bodies such as institutional review boards (IRB), grant funders, and journal editors are all key to improving the inclusivity of research studies. The IRBs could encourage studies to include NELP patients and require stringent review of studies with plans to exclude NELP patients. Similarly, journal editors could critically question methods that exclude NELP patients purely for convenience or cost reasons. Institutions could consider separate funds to support the additional steps required to include NELP patients, such as translating study materials and hiring multilingual research staff. Grants could require a section dedicated to explaining how funds from the grant will be used toward the equitable enrollment of NELP patients.

It is important to note we did not review any studies that did not mention language in the abstract or manuscript. Thus, it is possible that these studies excluded NELP patients without explicitly describing this in their paper, which could further exacerbate disparities in enrollment. This underscores the importance of creating a standard that requires publications to state the languages their participants speak and of reading studies through a critical lens if they exclude NELP patients.

It is an unrealistic expectation for every study to have the capacity to consent and enroll every non-English language. We advocate for a minimum of one non-English language with encouragement and incentive for more. The non-English language(s) included should be reflective of the general population served by the institution.

## LIMITATIONS

Although our methods covered a diverse set of search engines and terms, a limitation of this study is the potential for missed studies, including those published prior to 2018 and studies that did not discuss language of participants. However, it is possible that studies that do not discuss language excluded NELP patients from participating without explicitly remarking on this aspect of their enrollment process. Other limitations include the absence of information about reasons for exclusion or other barriers to inclusion (eg, time of enrollment availability), or size of the study (eg, multisite vs single site). Additionally, we were able to perform only descriptive statistics with these data, which limited our ability to draw conclusions regarding associations between characteristics of studies and the exclusion of NELP patients.

## CONCLUSION

This focused narrative review fills an important gap in the current literature describing the rates of exclusion of non-English language preferred patients in adult ED clinical research. Over half of the studies reviewed excluded NELP patients, with higher rates among interventional studies. It is imperative that interventional studies improve their NELP patient participation to be reflective of the general population. Institutional review boards, grant funders, and journal editors can encourage researchers to prioritize inclusionary practices. Future studies can include more robust statistical analyses that investigate associations between NELP patient research inclusion and study type.

## Supplementary Information















## Figures and Tables

**Table 1:** Descriptive statistics of extracted studies examining clinical research conducted in the emergency department and prehospital setting.

		Excluded NELP *n* (%)	Included NELP *n* (%)	Did Not Specify *n* (%)
Topic of Study	Health Equity/Social EM	63 (47%)	70 (52%)	2 (1%)
	Other	100 (68%)	40 (27%)	6 (4%)
Study included Intervention	Yes	74 (69%)	29 (27%)	4 (4%)
	No	89 (51%)	81 (47%)	4 (2%)
Clinical setting of study	Only in ED / Prehospital care	157 (59%)	105 (39%)	6 (2%)
	ED/Prehospital + Other	6 (46%)	5 (38%)	2 (15%)
Data acquisition	Primary Enrollment	134 (58%)	89 (39%)	8 (3%)
	Secondary Analysis	29 (58%)	21 (42%)	0 (0%)
Publication type	Abstract	25 (50%)	23 (46%)	2 (4%)
	Manuscript	138 (60%)	87 (38%)	6 (3%)
Included <18 years of age	Yes	14 (58%)	9 (38%)	1 (4%)
	No	145 (59%)	93 (38%)	6 (2%)
	Did not specify	4 (31%)	8 (62%)	1 (8%)
Year published	2018	30 (61%)	18 (37%)	1 (2%)
	2019	47 (69%)	19 (28%)	2 (3%)
	2020	37 (52%)	33 (46%)	1 (1%)
	2021	30 (54%)	24 (43%)	2 (4%)
	2022	18 (53%)	15 (44%)	1 (3%)
	2023	1 (33%)	1 (33%)	1 (33%)

*ED*, emergency department; *NELP*, non-English language preferred; *EM*, emergency medicine.
