# Supplementary material for: Narrative Review of Emergency Medicine Clinical Research Examining Exclusion by Language
Source: West J Emerg Med. 2025 Sep 25;26(5):1260–4. doi: 10.5811/westjem.46547 (PMC12591656; doi:10.5811/westjem.46547)
Supplement: Supplementary file 2 [file wjem-26-1260-s002.docx]

**Supplemental Table 2, Full Search Strategy for PubMed Central Searched on 3/14/23**

|  | emergenc*[Title] OR emergenc*[Abstract] OR emergent care[Title] OR emergent care[Abstract] OR emergicenter[Title] OR emergicenter[Abstract] OR emergicenters[Title] OR emergicenters[Abstract] OR trauma center[Title] OR trauma center[Abstract] OR trauma centers[Title] OR trauma centers[Abstract] OR trauma unit[Title] OR trauma unit[Abstract] OR trauma units[Title] OR trauma units[Abstract] OR acute care[Title] OR acute care[Abstract] OR immediate response[Title] OR immediate response[Abstract] OR prehospital care[Title] OR prehospital care[Abstract] OR pre hospital care[Title] OR pre hospital care[Abstract] OR 911 dispatch*[Title] OR 911 dispatch*[Abstract] OR "9 1 1" dispatch*[Title] OR "9 1 1" dispatch*[Abstract] OR EMS[Title] OR EMS[Abstract] OR paramedic*[Title] OR paramedic*[Abstract] OR EMT[Title] OR EMT[Abstract] OR EMTs[Title] OR EMTs[Abstract] OR first responder[Title] OR first responder[Abstract] OR first responders[Title] OR first responders[Abstract] OR ET3[Title] OR ET3[Abstract] OR triag*[Title] OR triag*[Abstract] OR ambulance[Title] OR ambulance[Abstract] OR ambulances[Title] OR ambulances[Abstract] OR "Emergency Medicine"[mesh:noexp] OR "Emergency Medical Technicians"[mesh:noexp] OR "Emergency Medical Services"[mesh:noexp] OR "Emergency Service, Hospital"[mesh] OR "Emergency Treatment"[mesh:noexp] OR "Evidence-Based Emergency Medicine"[mesh] OR "Emergency Medical Service Communication Systems"[mesh] OR "Emergency Medical Dispatch"[mesh] OR "Paramedics"[mesh] OR "Emergency Responders"[mesh:noexp] OR "Emergency Services, Psychiatric"[mesh] OR "Triage"[mesh] OR "Ambulances"[mesh] OR "Ambulance Diversion"[mesh] |
| --- | --- |
|  | limit*[Text Word] OR exclud*[Text Word] OR exclusion*[Text Word] OR filter*[Text Word] OR remov*[Text Word] |
|  | translat*[Title] OR translat*[Abstract] OR bilingual*[Title] OR bilingual*[Abstract] OR multilingual*[Title] OR multilingual*[Abstract] OR language[Title] OR language[Abstract] OR languages[Title] OR languages[Abstract] OR English[Text Word] OR NES[Title] OR NES[Abstract] OR LEP[Title] OR LEP[Abstract] OR "Language"[mesh:noexp] OR "Limited English Proficiency"[mesh] OR "Multilingualism"[mesh] OR "Translating"[mesh] |
| Article attributes: | Medline journals |
| Publication date: | From 2018/01/01 to 2023/04/01 |
