# Supplementary material for: Narrative Review of Emergency Medicine Clinical Research Examining Exclusion by Language
Source: West J Emerg Med. 2025 Sep 25;26(5):1260–4. doi: 10.5811/westjem.46547 (PMC12591656; doi:10.5811/westjem.46547)
Supplement: Supplementary file 3 [file wjem-26-1260-s003.docx]

**Supplemental Table 3, Full Search Strategy for Embase.com Searched on 3/14/23**

| #1 | (emergenc* OR 'emergent care' OR emergicenter? OR 'trauma center?' OR 'trauma unit?' OR 'acute care' OR 'immediate response' OR 'prehospital care' OR 'pre hospital care'):ab,ti,kw OR 'emergency medicine'/de OR 'emergency care'/exp OR 'emergency treatment'/de OR 'evidence based emergency medicine'/exp OR 'emergency health service'/exp OR 'first responder (person)'/de OR 'rescue personnel'/exp OR ('911 dispatch*' OR '9 1 1 dispatch*' OR EMS OR paramedic* OR EMT? OR 'first responder?'):ab,ti,kw OR (ET3 OR triag*):ab,ti,kw OR 'ambulance'/exp OR ambulance?:ab,ti,kw |
| --- | --- |
| #2 | (remov* OR limit* OR exclud* OR exclusion* OR filter*):ti,ab,kw OR 'exclusion criteria'/exp |
| #3 | 'multilingualism'/exp OR 'translating (language)'/exp OR (translat* OR bilingual* OR multilingual* OR English OR language? OR NES OR LEP):ab,ti,kw OR 'language'/de OR 'limited English proficiency'/exp OR 'second language proficiency'/exp OR 'English as a second language'/exp OR 'English (language)'/exp |
| #4 | #1 AND #2 AND #3 |
| #5 | #4 AND (2018:py OR 2019:py OR 2020:py OR 2021:py OR 2022:py OR 2023:py) |
