# Supplementary material for: Narrative Review of Emergency Medicine Clinical Research Examining Exclusion by Language
Source: West J Emerg Med. 2025 Sep 25;26(5):1260–4. doi: 10.5811/westjem.46547 (PMC12591656; doi:10.5811/westjem.46547)
Supplement: Supplementary file 4 [file wjem-26-1260-s004.docx]

**Supplemental Table 4, Full Search Strategy for Cochrane Searched on 3/14/23**

| #1 | (emergenc* OR emergent care OR emergicenter? OR trauma center? OR trauma unit? OR acute care OR immediate response OR prehospital care OR pre hospital care).ti,ab,kf. OR Emergency Medicine/ OR Emergency Medical Technicians/ OR Emergency Medical Services/ OR exp Emergency Service, Hospital/ OR Emergency Treatment/ OR exp Evidence-Based Emergency Medicine/ OR exp Emergency Medical Service Communication Systems/ OR exp Emergency Medical Dispatch/ OR exp Paramedics/ OR (911 dispatch* OR "9 1 1" dispatch* OR EMS OR paramedic* OR EMT? OR first responder?).ti,ab,kf. OR Emergency Responders/ OR exp Emergency Services, Psychiatric/ OR exp Triage/ OR (ET3 OR triag*).ti,ab,kf. OR exp Ambulances/ OR ambulance?.ti,ab,kf. OR exp Ambulance Diversion/ |
| --- | --- |
| #2 | (remov* OR limit* OR exclud* OR exclusion* OR filter*).ti,ab,kf. |
| #3 | exp Multilingualism/ OR exp Translating/ OR (translat* OR bilingual* OR multilingual* OR language? OR English OR NES OR LEP).ti,ab,kf. OR Language/ OR exp Limited English Proficiency/ |
| #4 | 1 and 2 and 3 |
| #5 | Limit 4 to yr="2018-2023" |
