# Supplementary material for: Narrative Review of Emergency Medicine Clinical Research Examining Exclusion by Language
Source: West J Emerg Med. 2025 Sep 25;26(5):1260–4. doi: 10.5811/westjem.46547 (PMC12591656; doi:10.5811/westjem.46547)
Supplement: Supplementary file 5 [file wjem-26-1260-s005.docx]

**Supplemental Table 5, Full Search Strategy for Web of Science Searched on 3/13/23**

| #1 | TS=(emergenc* OR "emergent care" OR emergicenter? OR "trauma center?" OR "trauma unit?" OR "acute care" OR "immediate response" OR "prehospital care" OR "pre hospital care" OR "911 dispatch*" OR "9 1 1 dispatch*" OR EMS OR paramedic* OR EMT? OR "first responder?" OR ET3 OR triag* OR ambulance?) |
| --- | --- |
| #2 | TS=(translat* OR bilingual* OR multilingual* OR language? OR English OR NES OR LEP) |
| #3 | TS=(remov* OR limit* OR exclud* OR exclusion* OR filter*) |
| #4 | #1 AND #2 AND #3 |
| Publication date: | 2018-01-01 to 2023-04-01 |
