# Supplementary material for: Narrative Review of Emergency Medicine Clinical Research Examining Exclusion by Language
Source: West J Emerg Med. 2025 Sep 25;26(5):1260–4. doi: 10.5811/westjem.46547 (PMC12591656; doi:10.5811/westjem.46547)
Supplement: Supplementary file 6 [file wjem-26-1260-s006.docx]

**Supplemental Table 6, Full Search Strategy for ClinicalTrials.gov Searched on 3/14/23**

| Condition OR disease: | emergency OR emergencies OR trauma center OR trauma centers OR trauma unit OR trauma units OR acute OR prehospital OR pre hospital OR 911 OR EMS OR paramedic OR paramedics OR EMT OR EMTs OR ET3 OR triage OR triaged OR triages OR triaging OR ambulance |
| --- | --- |
| Other terms: | **English OR language OR languages OR** translate OR translation OR translated OR translates OR bilingual OR bilingualism OR multilingual OR multilingualism OR NES OR LEP |
| Recruitment: | Enrolling by invitation  Recruiting  Completed |
| Study start: | 01/01/2018 To 04/01/2023 |
