# Supplementary material for: Narrative Review of Emergency Medicine Clinical Research Examining Exclusion by Language
Source: West J Emerg Med. 2025 Sep 25;26(5):1260–4. doi: 10.5811/westjem.46547 (PMC12591656; doi:10.5811/westjem.46547)
Supplement: Supplementary file 7 [file wjem-26-1260-s007.docx]

Supplemental Table 7: Extraction Data for Studies Conducting Clinical Research in the ED or Pre-Hospital Setting

| **Title** | **Authors** | **Year** | **Journal or Conference** | **Publication Type** | **Objective of Study** | **Topic of Study** | **Data Acquisition** | **Clinical Setting of Study** | **Study Design** | **Intervention** | **Languages Recruited** | **Include <18 Years Old** | **Other Inclusion Criteria** | **Other Exclusion Criteria** |
| --- | --- | --- | --- | --- | --- | --- | --- | --- | --- | --- | --- | --- | --- | --- |
| Adhesive Tape Placement on Patients' Masks in the ED Increases Compliance of Proper Face Mask Use | Nicholas Pettit, DO, PhD; Ali Zaidi, DO; Brian O'Neill, MD; Marla Doehring, MD | 2022 | Annals of Emergency Medicine | Manuscript | Does applying adhesive tape to the nasal bridge of a mask worn by emergency department (ED) patients improve adherence with universal mask wearing in the ED? | Other clinical study | Primary enrollment | No, only ED | RCT | Yes | English; Spanish | No | all ED pts | pregnant; prisoners; intoxicated or with decompensated psychiatric illness; presenting to the ED with a life-threatening condition; allergic to standard tape and/or Tegaderm |
| Patient preferences for treatment of acute bacterial skin and skin structure infections in the emergency department | Safa S. Almarzoky Abuhussain, Michelle A. Burak, Kelsey N. Kohman, Gabrielle Jacknin, Serina B. Tart, Athena L. V. Hobbs, Danyel K. Adams, Michael D. Nailor, Katelyn R. Keyloun, David P. Nicolau and Joseph L. Kuti | 2018 | BMC Health Services Research | Manuscript | Understand patient preferences for treatment of their acute bacterial skin and skin structure infections | Other clinical study | Primary enrollment | No, only ED | Cross sectional study | No | English | No | Adult patients (>18 years of age) who presented to the ED with acute bacterial skin and skin structure infections | acutely-ill and met the Center for Medicare and Medicaid Services (CMS) definition for severe sepsis, if they had suspected necrotizing fasciitis or osteomyelitis, or if they were unable to provide written informed consent |
| High prevalence of injection drug use and blood-borne viral infections among patients in an urban emergency department | Erik S. Anderson, Carly Russell, Kellie Basham, Martha Montgomery, Helen Lozier, Abigail Crocker, Marisa Zuluaga, Douglas A. E. White | 2020 | PLOS ONE | Manuscript | Assess the prevalence of injection drug use and co-occurring HIV and HCV infection among patients presenting to an urban ED. | Health equity/social EM | Primary enrollment | No, only ED | Cross sectional study; Prevalence study | No | English; Spanish | No | >18 years | medically unstable, unable to provide informed consent |
| Social determinants of health associated with hemodialysis non-adherence and emergency department utilization: a pilot observational study | Kamna S. Balhara, Lori Fisher, Naya El Hage, Rosemarie G. Ramos, and Bernard G. Jaar | 2020 | BMC Nephrology | Manuscript | Identify social determinants of health (SDOH) associated with missing hemodialysis (HD) and presenting to the ED, and describe resource utilization associated with such visits | Health equity/social EM | Primary enrollment | Other: outpatient HD clinic | Cross sectional study; Other: Case control | No | English | No | missed at least one HD session prior to ED visit at site 1, were over age 18 | patients who had already participated, were deemed critically ill by ED clinicians, or were unable to consent |
| Factors Associated with Lack of HIV Testing among Latino Immigrant and Black Patients at 4 Geographically and Demographically Diverse Emergency Departments | Christopher L. Bennett, MD, MA, Sarah J. Marks, MS, Tao Liu, Melissa A. Clark, PhD, Michael P. Carey, PhD, and Roland C. Merchant, MD, MPH, ScD | 2020 | Journal of the International Association of Providers of AIDS Care | Manuscript | Assessing the needs for HIV testing among Latino immigrants and Blacks | Health equity/social EM | Secondary analysis | No, only ED | Cross sectional study | No | English; Spanish | No | 18-64 years-old and able to provide consent and participate in the study | incarcerated; cognitive impairment or other disability that prevented study participation; intoxicated, critically ill, HIV infected, or taking pre-exposure prophylaxis, enrolled in other HIV studies, unable to read at a second-grade level in English or Spanish |
| Barriers to recruitment into emergency department-initiated palliative care: a sub-study of a multi-site, randomized controlled trial | Julia Brickey, Mara Flannery, Allison Cuthel, Jeanne Cho, Corita R. Grudzen and The EMPallA Investigators | 2022 | BMC Palliative Care | Manuscript | Explores the barriers to enrolling seriously ill patients scheduled for discharge from the ED into palliative care research | Other clinical study | Secondary analysis | No, only ED | RCT; Cross sectional study | Yes | English; Spanish | No | patients 50 years of age or older who presented to the ED with a qualifying illness (defined as advanced cancer or end-stage organ failure) and were scheduled for ED discharge or observation status, have health insurance, and reside within the geographic area | previously received hospice or palliative care, were admitted to inpatient services, had dementia, or lived in a skilled nursing facility or similar assisted living facility |
| The Role of Specific Sources of Social Support on Post-Injury Psychological Symptoms | Ashley Brienza, Brian P. Suffoletto, Eric Kuhn, Anne Germain, Stephany Jaramillo, Melissa Repine, Clifton W. Callaway, and Maria L. Pacella-LaBarbara | 2021 | Rehabilitation Psychology | Manuscript | Among a sample of acutely injured patients within 24 hr post-MVC, we aimed to examine whether perceived social support (total and from specific sources) contributes to PTSD symptoms (PTSS) and associated comorbidities at 90 days postinjury. | Other clinical study | Secondary analysis | No, only ED | RCT | Yes | English | No | ages of 18> presenting within 24 hr of MVC or motorcycle-crash-related injury, presence of a musculoskeletal injury, ownership of a smartphone with the ability of downloading apps, endorsement of Criterion A of the Diagnostic and Statistical Manual of Mental Disorders PTSD diagnosis, and, self reported pain intensity 1-4 using the verbal rating scale | major lacerations resulting in significant loss of subcutaneous tissue and nerve injury, moderate to severe traumatic brain injury with a Glasgow Coma Scale score <13, self-inflicted injury, presence of a neurologic disease, administration of benzodiazepines or other psychotropic medication in the ED, actively receiving psychotherapy or pharmacological treatment for PTSD or other psychiatric disorder, MVC/motorcycle crash caused by a medical condition |
| Performance of 2 Single-Item Screening Questions to Identify Future Homelessness Among Emergency Department Patients | Thomas Byrne, PhD; Mindy Hoang; Ann Elizabeth Montgomery, PhD; Eileen Johns, MPA; Marybeth Shinn, PhD; Tod Mijanovich, PhD; Dennis Culhane, PhD; Kelly M. Doran, MD, MHS | 2022 | JAMA Network Open | Manuscript | Examining the performance of housing-related screening questions in healthcare settings | Health equity/social EM | Primary enrollment | No, only ED | Cohort study | No | English; Spanish | No | aged 18 years or older | medically unstable, in psychological distress, in police or prison custody, could not provide informed consent (eg, had dementia or were too intoxicated to participate), did not live in NYC, or had already participated |
| A Brief Music App to Address Pain in the Emergency Department: Prospective Study | Peter R Chai, MA, MD; Emily Schwartz, BA; Mohammad Adrian Hasdianda, MD; Desiree R Azizoddin, PsyD; Anna Kikut, BA; Guruprasad D Jambaulikar, MPH, MD; Robert R Edwards, PhD; Edward W Boyer, MD,PhD; Kristin L Schreiber, MD, PhD | 2020 | JOURNAL OF MEDICAL INTERNET RESEARCH | Manuscript | Music as a pain management tool for ED patients | Other clinical study | Primary enrollment | No, only ED | Cohort study | Yes | English | No | patients with ED observation unit stays of up to 48 hours and who had received orders for opioid analgesia | pacemaker, had hearing loss, were on contact precautions, had previously enrolled in the study, were unstably housed, or had a significant active medical or psychiatric illness |
| Analysis of Diagnoses, Symptoms, Medications, and Admissions Among Patients With Cancer Presenting to Emergency Departments | Jeffrey M. Caterino, MD, MPH; David Adler, MD, MPH; Danielle D. Durham, PhD, MPH; Sai-Ching Jim Yeung, MD, PhD; Matthew F. Hudson, PhD, MPH; Aveh Bastani, MD; Steven L. Bernstein, MD; Christopher W. Baugh, MD, MBA; Christopher J. Coyne, MD, MPH; Corita R. Grudzen, MD, MSHS; Daniel J. Henning, MD, MPH; Adam Klotz, MD; Troy E. Madsen, MD; Daniel J. Pallin, MD, MPH; Cielito C. Reyes-Gibby, DrPH; Juan Felipe Rico, MD; Richard J. Ryan, MD; Nathan I. Shapiro, MD; Robert Swor, DO; Arvind Venkat, MD; Jason Wilson, MD, MA; Charles R. Thomas Jr, MD; Jason J. Bischof, MD; Gary H. Lyman, MD, MPH | 2019 | JAMA Network Open | Manuscript | To provide a benchmark description of patients who present to the ED with active cancer | Other clinical study | Primary enrollment | No, only ED | Cohort study | No | English | No | adults (aged 18 years) with active cancer | pregnancy, incarceration, psychiatric chief complaint, primary evaluation as a trauma response, previous enrollment, or too ill or otherwise unable to participate in survey administration |
| A Qualitative Assessment to Understand the Barriers and Enablers Affecting Contraceptive Use Among Adolescent Male Emergency Department Patients | Lauren S. Chernick, MD, MSc, Jonathan Y. Siden, BSW, David L. Bell, MD, MPH, and Peter S. Dayan, MD, MSc | 2019 | American Journal of Men‚Äôs Health | Manuscript | Investigate if the ED is a potential site to implement interventions to minimize early fatherhood and unintended teenage pregnancy | Health equity/social EM | Primary enrollment | No, only ED | Cross sectional study | No | English | Yes | aged 14-19 years and ever sexually active with females | cognitively impaired, were in foster care or wards of the state, did not speak English, or were too ill for participation per the attending physician |
| Early hemodynamic assessment using NICOM in patients at risk of developing Sepsis immediately after emergency department triage | Steve B. Chukwulebe, David F. Gaieski, Abhishek Bhardwaj, Lakeisha Mulugeta-Gordon4, Frances S. Shofer, and Anthony J. Dean | 2021 | Scandinavian Journal of Trauma, Resuscitation, and Emergency Medicine | Manuscript | Investigated whether serial hemodynamic parameters obtained from a non-invasive cardiac output monitor (NICOM) predicts disease severity in patients at risk for sepsis. | Other clinical study | Primary enrollment | No, only ED | Cohort study | Yes | English | No | ESI 2-3, >18 years old, 2 of 3 SIRS criteria at triage, automated lactate order was generated by ED advance triage protocol | ESI 1, ESI 4-5, altered mental status, inability to obtain informed consent |
| Interrelationships of Economic Stressors, Mental Health Problems, Substance Use, and Intimate Partner Violence among Hispanic Emergency Department Patients: The Role of Language-Based Acculturation | Carol B. Cunradi, Raul Caetano, William R. Ponicki and Harrison J. Alter | 2021 | International Journal of Environmental Research and Public Health | Manuscript | Analyzed the interrelationships of economic stressors, mental health problems, substance use, and intimate partner violence among a sample of Hispanic emergency department patients | Health equity/social EM | Primary enrollment | No, only ED | Cross sectional study | No | English; Spanish | No | 18-50 years old; resident of the county in which the hospital is located; and married, cohabiting, or in a romantic (dating) relationship for the past 12 months | intoxicated, experiencing acute psychosis or suicidal or homicidal ideation; cognitively/psychologically impaired and unable to provide informed consent; held in custody by law enforcement; or in need of immediate medical attention |
| Frequency of Intimate Partner Violence among an Urban Emergency Department Sample: A Multilevel Analysis | Carol B. Cunradi, William R. Ponicki, Raul Caetano and Harrison J. Alter | 2021 | International Journal of Environmental Research and Public Health | Manuscript | The extent to which individual, household, and neighborhood factors are associated with the frequency of IPV among a socially disadvantaged sample of urban ED patient | Health equity/social EM | Primary enrollment | No, only ED | Cross sectional study | No | English; Spanish | No | 18-50 years old; resident of the county in which the hospital is located; and married, cohabiting, or in a romantic (dating) relationship for the past 12 months | intoxicated, experiencing acute psychosis or suicidal or homicidal ideation, were cognitively/psychologically impaired and unable to provide informed consent, in custody by law enforcement, or in need of immediate medical attention |
| Implementation facilitation to promote emergency department-initiated buprenorphine for opioid use disorder: protocol for a hybrid type III effectiveness-implementation study (Project ED HEALTH) | Gail D'Onofrio, E. Jennifer Edelman, Kathryn F. Hawk, Michael V. Pantalon, Marek C. Chawarski, Patricia H. Owens, Shara H. Martel, Paul VanVeldhuisen, Neal Oden, Sean M. Murphy, Kristen Huntley, Patrick G. O'Connor and David A. Fiellin | 2019 | Implementation Science | Manuscript | Evaluating the impact of implementation facilitation on the adoption of ED-initiated buprenorphine for OUD into practice | Health equity/social EM | Primary enrollment | No, only ED | RCT; Clinical trial; Protocol | Yes | English | No | >18 years of age, meet DSM-5 criteria for moderate/severe OUD, have a positive urine test for opioids, and consent to study procedures | medical or psychiatric condition requiring hospitalization at the index ED visit, are actively suicidal, are cognitively impaired, present from an extended care facility, require opioids for a pain condition, or are currently (past 30 days) enrolled in a formal OUD treatment program |
| Understanding diagnostic processes in emergency departments: a mixed methods case study protocol | Michelle Daniel, SunYoung Park, Colleen M Seifert, P Paul Chandanabhumma, Michael D Fetters, Eric Wilson, Andrew B Canvasser, Hardeep Singh, Kalyan Pasupathy, Prashant Mahajan | 2021 | BMJ Open | Manuscript | Describe a study protocol to map diagnostic processes in the ED as a foundation for developing future error mitigation strategies | Other clinical study | Primary enrollment | No, only ED | Qualitative ; Protocol | No | English | Yes | Eligible adult patients will be 21 or older and capable of giving informed consent. Eligible paediatric patients will be between 0 and 21 years of age and their legally authorised representative must be capable of giving informed consent. For paediatric patients 13 years of age or older, assent will also be required | altered mental status |
| Acupuncture in the emergency department for pain management: A BraveNet multi-center feasibility study | Jeffery A. Dusek, PhD, Gene A. Kallenberg, MDc, Robert M. Hughes, DO, Alan B. Storrow, MD, Christopher J. Coyne, MD, David R. Vago, PhD, Arya Nielsen, PhD, Alison Karasz, PhD Ryung S. Kim, PhDk, Jessica Surdam, MPHa , Tracy Segall, MSHSa, M. Diane McKee, MD | 2022 | Medicine | Manuscript | Acupuncture as an alternative opioid-sparing approach to pain reduction | Other clinical study | Primary enrollment | No, only ED | RCT; Protocol | Yes | English | No | 18 years or older, able to communicate in English, a level 3, 4, or 5 on the triage rating scale, adults presenting to the ED with acute non-emergent (musculoskeletal, back, pelvic, non-cardiac chest, abdominal, flank or head) pain >4 on a 0-10-point Numeric Rating Scale due to non-penetrating injury | fever exceeding 100F, presenting with a chief complaint of a psychological/psychiatric concern, presenting with a chief complaint of migraine, current pregnancy, self-reported or documented opioid medication taken orally within 4 hours, presenting with chief complaint of joint dislocation, presenting with chief complaint of bone fracture, or confirmed or suspected COVID-19 infection |
| Emergency Department Screening for Unhealthy Alcohol and Drug Use with a Brief Tablet-Based Questionnaire | Joshua W. Elder, Evan F. Wu, James A. Chenoweth, James F. Holmes, Aman K. Parikh, Aimee K. Moulin, Tommie G. Trevino, and John R. Richards | 2020 | Emergency Medicine International | Manuscript | Determine the feasibility and utility of a brief tablet-based screening method in the ED and if patients would consider a face-to-face meeting with a certified alcohol and drug counselor for more in-depth screening, brief intervention, and referral to treatment helpful via this interface | Health equity/social EM | Primary enrollment | No, only ED | Cross sectional study | Yes | English | No | 1) age>18 years, (2) ESI 2-5 | any evidence of acute intoxication and/or having received sedating medication while in the ED, previous enrollment |
| Facemasks: Perceptions and use in an ED population during COVID-19 | Vidya EswaranI, Anna Marie Chang, R. Gentry Wilkerson, Kelli N. O'Laughlin, Brian Chinnock, Stephanie A. Eucker, Brigitte M. Baumann, Nancy Anaya, Daniel G. Miller, Adrianne N. Haggins, Jesus R. Torres, Erik S. Anderson, Stephen C. Lim, Martina T. CaldwellID, Ali S. Raja, Robert M. Rodriguez, The REVVED-UP Investigators | 2022 | PLOS ONE | Manuscript | To assess beliefs, access, and practices of mask wearing across 15 urban ED populations | Other clinical study | Secondary analysis | No, only ED | Cross sectional study | No | English; Spanish; Portuguese ; French; Creole; Chinese (including Mandarin); Arabic; Vietnamese ; Korean; Russian; Other: Bengali, Tagalog | Yes | adult ED patients | major trauma, transfer from other facilities, incarceration, psychiatric hold, intoxication, altered mental status, critical illness, and temporary visit from other countries |
| Acupuncture for acute musculoskeletal pain management in the emergency department and continuity clinic: a protocol for an adaptive pragmatic randomised controlled trial | Stephanie A Eucker, Oliver Glass, Catherine A Staton, Mitchell R Knisely, Amy O'Regan, Christi De Larco, Michelle Mill, Austin Dixon, Olivia TumSuden, Erica Walker, Juliet C Dalton Alexander Limkakeng, Ann Miller W Maxwell, Alex Gordee, Maggie Kuchibhatla, Sheinchung Chow | 2022 | BMJ Open | Manuscript | Acupuncture is increasingly recognised as a safe, affordable and effective treatment for pain and anxiety in the clinic setting, but it has yet to be established as a primary treatment option in the ED | Other clinical study | Primary enrollment | No, only ED | Protocol | Yes | English | No | adult (age 18 years or older) ED patients with pain in the neck, back, arms and/or legs and a clinical diagnosis of acute (<7days) musculoskeletal pain as determined by an ED provider | 1) suspected to have a non-musculoskeletal cause of pain, (2) unable to receive acupuncture due to injury, infection or other contraindication to the use of needles at acupuncture sites; (3) not possible to attend outpatient clinic (eg, visiting from out-of-state); (4) unable to provide informed consent or to comprehend or complete study measures or procedures due to cognitive impairment, including evidence of drug, medication or alcohol intoxication, or due to severe hearing or speech impairment; (5) unable to safely participate due to critical illness, obvious bony deformity, other serious medical condition (including active COVID-19 infection) and/or based on ED provider judgement |
| Association of a Callback Program With Emergency Department Revisit Rates Among Patients Seeking Emergency Care | Scott Fruhan, MD; Corey B. Bills, MD, MPH | 2022 | JAMA Network Open | Manuscript | To assess the association of an automated telephone call 2 days after ED discharge with the likelihood of an unplanned ED revisit at both 72 hours and 7 days after the index visit | Health equity/social EM | Primary enrollment | No, only ED | RCT; Clinical trial | Yes | English; Spanish; Portuguese ; French; Creole; Chinese (including Mandarin); Arabic; Vietnamese ; Korean; Russian | Yes | any age, seen by an emergency care clinician and subsequently discharged from the ED, left against medical advice, or left prior to formal discharge after the conclusion of their ED care | without a documented US-based 10-digit telephone number in the medical record were excluded from the study, admitted to the hospital, transferred to another inpatient facility, discharged to jail, or who left prior to being seen or prior to |
| Perspectives of COVID-19 vaccine-hesitant emergency department patients to inform messaging platforms to promote vaccine uptake | Alexzandra T. Gentsch LSW, Jonathan Butler PhD, Kelli O'Laughlin MD, MPH, Stephanie A. Eucker MD, PhD, AnnaMarie Chang MD, MSC, Herbie Duber MD, MPH, Rachel E. Geyer MPH  \| Amanda Guth MPH1 \| Hemal K. Kanzaria MD \|  Alena Pauley MScGH \| Kristin L. Rising MD, MSHP \| Cecilia Lara Chavez \| Anna Tupetz DPT \| Robert M. Rodriguez MD | 2022 | Society for Academic Emergency Medicin | Manuscript | Sought to elicit the perspectives of unvaccinated ED patients regarding COVID-19 vaccination concerns and | Other clinical study | Primary enrollment | No, only ED | Qualitative | No | English; Spanish; Other: only enrolled Spanish-speaking participants at 1/4 sites | No | age>18years, had not received a COVID-19 vaccine, anticipated ability to complete a 30- to 40-min interview | unable to provide consent to or complete an interview because of major trauma, intoxication, altered mental status, or critical illness; presenting with a psychiatric chief complaint; in police custody; or under suspicion of acute COVID-19 illness |
| Randomised clinical trial of an emergency department-based peer recovery support intervention to increase treatment uptake and reduce recurrent overdose among individuals at high risk for opioid overdose: study protocol for the navigator trial | William C Goedel, Brandon D L Marshall, Elizabeth A Samuels, Mark G Brinkman, Debra Dettor, Kirsten J Langdon, Linda A Mahoney, Roland C Merchant, Tarek Nizami, George A O'Toole, Susan E Ramsey, Jesse L Yedinak, Francesca L Beaudoin | 2019 | BMJ Open | Manuscript | Compare the effectiveness of behavioural interventions delivered in the ED by certified peer recovery support specialists relative to those delivered by licensed clinical social  workers in promoting OUD treatment uptake and reducing recurrent ED visits for opioid overdose | Health equity/social EM | Primary enrollment | No, only ED | Protocol | Yes | English | No | >18 years old, being treated for an opioid overdose; receiving treatment related to OUD (eg, infectious complication of injection drug use, opioid withdrawal); or self-report an opioid overdose within the previous 12 months | critically ill or injured, have previously enrolled in the trial, are in police custody or incarcerated, pregnant, or live outside of Rhode Island |
| Developing a pulse oximetry home monitoring protocol for patients suspected with COVID-19 after emergency department discharge | David B Gootenberg, Nicholas Kurtzman, Thomas O'Mara, Jennifer Y Ge, David Chiu, Nathan Shapiro, Oren J Mechanic, Alon Dagan | 2021 | BMJ Health and Care Informatics | Manuscript | Assess feasibility and describe a protocol for ED-based outpatient pulse-oximetry monitoring with structured follow-up and determine rates of ED return, hospitalisation and hypoxia among participants | Other clinical study | Primary enrollment | No, only ED | Cohort study; Other: Prospective observational study | No | English; Spanish; Creole; Chinese (including Mandarin); Vietnamese | No | adult patient with presumed or confirmed COVID-19, otherwise dischargeable (eg, able to self-isolate, well appearing) and at risk for outpatient decompensation | need for admission, resting oxygen saturation <92%, ambulatory oxygen saturation <90%, resting heart rate >110 beats per minute or a patient was unable to use the pulse oximetry device |
| Emergency Medicine Palliative Care Access (EMPallA): Protocol for a multicentre randomised controlled trial comparing the effectiveness of specialty outpatient versus nurse-led telephonic palliative care of older adults with advanced illness | Corita R Grudzen, Deborah J Shim, Abigail M Schmucker, Jeanne Cho,  Keith S Goldfeld, The EMPallA Investigators | 2019 | BMJ open | Manuscript | This study aims to compare nurse-led telephonic case management to specialty outpatient palliative care for older adults with serious, life-limiting illness on: (1) quality of life in patients; (2) healthcare utilisation; (3) loneliness and symptom burden and (4) caregiver strain, caregiver quality of life and bereavement. | Other clinical study | Primary enrollment | No, only ED | RCT; Protocol | Yes | English | No | aged 50+ years with a serious, life-limiting condition | Dementia, received hospice, services or two or more palliative care visits in the last 6 months, or reside in a skilled nursing or assisted living facility or chronic care hospital |
| Assessment of Patient-Centered Approaches to Collect Sexual Orientation and Gender Identity Information in the Emergency Department: The EQUALITY Study | Adil Haider, MD, MPH; Rachel R. Adler, ScD; Eric Schneider, PhD; Tarsicio Uribe Leitz, MD, MPH; Anju Ranjit, MD, MPH; Christina Ta, BA; Adele Levine, MPH; Omar Harfouch, MD, MPH; Danielle Pelaez, BA; Lisa Kodadek, MD; Laura Vail, MS; Claire Snyder, PhD; Danielle German, PhD; Susan Peterson, MD; Jeremiah D. Schuur, MD, MHS; Brandyn D. Lau, MPH, CPH | 2018 | JAMA Network Open | Manuscript | To assess of the optimal patient-centered approach for sexual orientation and gender identity collection in the emergency department setting | Health equity/social EM | Primary enrollment | No, only ED | Cohort study | Yes | English | No | older than18 years were eligible to have SOGI information collected, every patient who identified themselves as a SGM | chief concern was psychiatric or alcohol and/or drug-related, or who had an Emergency Severity Index rating of 1 |
| Delirium and its association with short-term outcomes in younger and older patients with acute heart failure | Jin H. HanID, Candace D. McNaughton, William B. Stubblefield, Peter S. Pang, Phillip D. Levy, Karen F. Miller, Sarah Meram, Mette Lind Cole, Cathy A. Jenkins, Hadassah H. Paz, Kelly M. Moser, Alan B. Storrow, Sean P. Collins, Emergency Medicine Research and Outcomes Consortium Investigators | 2022 | PLOS ONE | Manuscript | To determine if delirium was associated with short-term adverse outcomes in a diverse cohort of younger and older patients with acute heart failure | Other clinical study | Primary enrollment | No, only ED | Cohort study | No | English | No | within 12 hours of ED arrival if they were 18 years of age or older, had a confirmed clinical diagnosis of AHF | did not met any of the following criteria: BNP >100 pg/mL, NT-proBNP > 900 pg/mL, radiographic or sonographic signs of pulmonary congestion, or treatment for AHF with intravenous diuretics or vasodilators |
| Feasibility and acceptability of electronic administration of patient reported outcomes using mHealth platform in emergency department patients with non-medical opioid use | Kathryn Hawk , Caitlin Malicki, Jeremiah Kinsman, Gail D'Onofrio, Andrew Taylor and Arjun Venkatesh | 2021 | Addiction Science & Clinical Practice | Manuscript | Feasibility and acceptability of electronically collecting patient reported outcomes from  ED patients with non-medical opioid use to enhance care in the ED and transitions of care | Health equity/social EM | Primary enrollment | No, only ED | Cohort study | Yes | English | No | patients presenting to the YNHH ED for opioid overdose or those screening positive for OUD or non-medical opioid use, based on the NIDA Quick Screen, a brief 30-day substance use screener | age < 18 years, non-English speaking, active psychiatric evaluation, and inability to provide consent or follow-up contact information |
| Perspectives About Emergency Department Care Encounters Among Adults With Opioid Use Disorder | Kathryn Hawk, MD, MPH; Ryan McCormack, MD; E. Jennifer Edelman, MD; Edouard Coupet Jr, MD; Nicolle Toledo, RN; Phoebe Gauthier, MPH; John Rotrosen, MD; Marek Chawarski, PhD; Shara Martel, MPH; Patricia Owens, MS; Michael V. Pantalon, PhD; Patrick O‚ÄôConnor, MD; Lauren K. Whiteside, MD; Ethan Cowan, MD; Lynne D. Richardson, MD; Michael S. Lyons, MD; Richard Rothman, MD; Lisa Marsch, PhD; David A. Fiellin, MD; Gail D'Onofrio, MD | 2022 | JAMA Network Open | Manuscript | To assess the experiences and perspectives regarding ED-initiated health care and OUD treatment among US patients with untreated OUD seen in the ED | Health equity/social EM | Secondary analysis | No, only ED | Qualitative | No | English | No | recruited during an ED visit before the formal parent study implementation-facilitation period | not engaged in OUD treatment when they presented at their ED visit, were not in jail or prison, had capacity to provide informed consent, and either met Diagnostic and Statistical Manual of Mental Disorders (Fifth Edition) criteria for OUD (Project ED HEALTH) or endorsed recurrent illicit opioid use and self-identified as having an opioid problem |
| Association of Oral Health Literacy and Dental Visitation in an Inner-City Emergency Department Population | Emmett Henderson, Preeti Dalawari, Jennifer Fitzgerald, and Leslie Hinyard | 2018 | International Journal of Environmental Research and Public Health | Manuscript | Examine the association between oral health literacy with sociodemographic variables and dental visitation in adults presenting to an urban ED | Health equity/social EM | Primary enrollment | No, only ED | Cross sectional study | No | English | No | age 18-90; no acute distress | sexual assault victims; incarcerated persons/prisoners or patients under arrest; psychiatric patients or homicidal/suicidal patients; patients incapacitated because of drugs or alcohol; patients unable to communicate effectively (e.g., hearing-impaired, intoxicated,or violent patients); and patients in distress/extremis |
| Acute pain pathways: protocol for a prospective cohort study | Molly Moore Jeffery, Mitra Ahadpour, Summer Allen, Richardae Araojo, Fernanda Bellolio, Nancy Chang, Laura Ciaccio, Lindsay Emanuel, Jonathan Fillmore, Gregg H Gilbert, Patricia Koussis, Christine Lee, Heather Lipkind, Celeste Mallama, Tamra Meyer, Megan Moncur, Teryl Nuckols, Michael A Pacanowski, David B Page, Elektra Papadopoulos, Jessica D Ritchie, Joseph S Ross, Nilay D Shah, Mat Soukup, Christopher O St. Clair, Stephen Tamang, Sam Torbati, Douglas W Wallace, Yueqin Zhao, Rebekah Heckmann | 2022 | BMJ Open | Manuscript | To better understand pain management and pain control among individuals experiencing acute pain for various common diagnoses | Other clinical study | Primary enrollment | No, only ED | Cohort study; Protocol | No | English | Yes | Age 18 and over; or age 15 and over undergoing third molar extraction at Mayo Clinic in Rochester, experiencing an acute pain condition of less than 8 weeks duration at the time opioids are offered, offered prescription for opioid analgesic to treat acute pain, opioid naive (no use of prescribed opioids or illicit opioids, including medical or non-medical use, in the past 6 months) by self-report, willing and able to give consent and participate in study, able to access a mobile device (smartphone or tablet)  or computer with web access to complete study questionnaires; able to connect Fit bit to a device that can regularly link to Hugo for data transfer, willing to use the health data aggregating platform, released/discharged to home after their visit | pain thought by treating clinician to be caused by a systemic disease very likely to progress to chronic pain (eg, sickle cell disease, fibromyalgia, lupus, multiple sclerosis), cancer or end-of-life related pain, unable to give consent and be enrolled within 3days of being offered the prescription |
| Interruption of initial patient assessment in the emergency department and its effect on patient perception of care quality | KIMBERLY D. JOHNSON, CHRISTOPHER J. LINDSELL, CRAIG FROEHLE, and GORDON LEE GILLESPIE5 | 2021 | International Journal for Quality in Health Care | Manuscript | to determine how the frequency and duration of interruptions during triage are associated with errors, patient satisfaction and patient‚Äôs perception of the care they received | Other clinical study | Primary enrollment | No, only ED | Cohort study | No | English | No | ED nurses, physicians and patients | a member of a vulnerable population (i.e. under age 18, incarcerated, cognitive impairment), assigned an ESI of 1 (high acuity), non-English speakers or declined to participate |
| Reducing Hospitalizations and Emergency Department Visits in Patients With Venous Thromboembolism Using a Multicomponent Care Transition Intervention | Alok Kapoor, MD, MSc, Sarah Bloomstone, BA, Saud Javed, MD, Matt Silva, PharmD, Ann Lynch, PharmD, Dinesh Yogaratnam, PharmD, BCPS, Brian Carlone, PharmD, Katelyn Springer, PharmD, Abiramy Maheswaran, MD,  Xiaoshuang Chen, MD, Ahmed Nagy, MD, Rasha Elhag, MD, Edna Markaddy, MD, Timothy Aungst, PharmD, Donna Bartlett, PharmD, BCGP, RPh, Diana Houng, PharmD, BCPS, Chad Darling, MD, David McManus, MD, Shoshana J. Herzig, MD, MPH,  Bruce Barton, PhD, and Kathy Mazor, EdD | 2020 | The Journal of Health Care Organization, Provision, and Financing | Manuscript | To assess the impact of a care transition intervention on hospitalizations and emergency department visits after venous thromboembolism | Other clinical study | Primary enrollment | Other: office | RCT | Yes | English | No | aged 18 and older with an episode of VTE diagnosed in the previous 2 weeks in hospital or ambulatory settings (office or ED) and prescribed  warfarin, direct oral anticoagulant, or low molecular weight heparin | discharged to a nursing home |
| A Prospective Observational Study of Emergency Department‚ÄìInitiated Physical Therapy for Acute Low Back Pain | Howard S Kim, MD, MS, Jody D Ciolino, PhD, Nicola Lancki, MPH, Kyle J Strickland, PT, DPT, Daniel Pinto, PT, PhD, Christine Stankiewicz, PT, DPT, D Mark Courtney, MD, MSCI, Bruce L Lambert, PhD, Danielle M McCarthy, MD, MS | 2020 | Physical Therapy | Manuscript | To compare patient-reported outcomes in patients receiving ED-initiated physical therapy and patients receiving usual care for acute low back pain | Other clinical study | Primary enrollment | No, only ED | Cohort study | No | English | No | presented to the ED with a complaint of acute low back pain | <18 years old, or did not have an email address or telephone number for follow-up data reporting |
| Embedded emergency department physical therapy versus usual care for acute low back pain: a protocol for the NEED-PT randomised trial | Howard S Kim, Kayla M Muschong, Ivy L Fishman, Jacob M Schauer, Amee L Seitz, Kyle J Strickland, Bruce L Lambert,5 Danielle M McCarthy, My H Vu, Jody D Ciolino | 2022 | BMJ Open | Manuscript | A randomised trial to evaluate physical therapy in the ED setting | Other clinical study | Primary enrollment | No, only ED | RCT; Protocol | Yes | English | No | 18 years, evaluated by a participating study physician during normal business hours (Monday to Friday, 08:00 to 16:00), anatomic low back (defined using the consensus international definition of pain located between the 12th rib and buttocks), symptom duration <30 days | chronic low back pain, any prior lumbar spine surgery, inability to ambulate at baseline, or any of the following as determined by the treating physician: obvious nonmusculoskeletal aetiology for low back pain (eg, shingles, kidney stone), other concomitant injuries or pain (eg, closed head injury and low back pain), red-flag symptoms indicating life-threatening pathology (bladder/bowel incontinence, saddle anaesthesia, debilitating motor weakness), or likely to be admitted to the hospital |
| Fears of disclosure and misconceptions regarding domestic violence reporting amongst patients in two US emergency departments | Leigh Kimberg, Juan A. Vasquez, Jennifer Sun, Erik Anderson, Clarissa Ferguson, Mireya Arreguin, Robert M. Rodriguez | 2021 | PLOS ONE | Manuscript | To assess undocumented Latino immigrants, Latino legal residents/citizens and non-Latino legal residents/citizens beliefs about disclosure of DV victimization to healthcare providers and healthcare provider reporting of DV to law enforcement and immigration authorities | Health equity/social EM | Primary enrollment | No, only ED | Cross sectional study | No | English; Spanish | Does not say | adult patients | being evaluated for trauma; transferred from another facility; unable to participate in an interview because of intoxication, altered mental status or critical illness; presenting while incarcerated; on a psychiatric hold |
| Evaluation of Diagnostic and Triage Accuracy and Usability of a Symptom Checker in an Emergency Department: Observational Study | Hamish S F Fraser, BSc, MBChB, MS; Gregory Cohan, BA; Christopher Koehler, BA, MMS, MD; Jared Anderson, BA, MD; Alexis Lawrence, BA, MD; John Patea, MA, MPH; Ian Bacher, BA, PhD; Megan L Ranney, BA, MPH, MD | 2022 | JMIR mHealth and uHealth | Manuscript | To determine the diagnostic and triage accuracy and usability of a symptom checker in use by patients presenting to an emergency department | Other clinical study | Primary enrollment | No, only ED | Cross sectional study | No | English | No | >18 years, presenting for emergency evaluation of a medical (nontrauma and non-mental health) problem, and deemed by the triage nurse to not be critically ill, had to be able to consent and complete the symptom checker assessment before physician evaluation |  |
| Implementation facilitation to introduce and support emergency department-initiated buprenorphine for opioid use disorder in high need, low resource settings: protocol for multi-site implementation-feasibility study | Ryan P. McCormack , John Rotrosen, Phoebe Gauthier, Gail D‚ÄôOnofrio, David A. Fiellin, Lisa A. Marsch, Patricia Novo, David Liu, E. Jennifer Edelman, Sarah Farkas, Abigail G. Matthews, Caroline Mulatya, Dagmar Salazar, Jeremy Wolff, Randolph Knight, William Goodman and Kathryn Hawk | 2021 | Addiction Science & Clinical Practice | Manuscript | To learn whether ED-initiated buprenorphine with referral for treatment of OUD is generalizable | Health equity/social EM | Primary enrollment | No, only ED | Protocol | Yes | English | No | adult ED patients who were determined by ED clinical staff to be eligible for and willing to receive BUP according to criteria in site-specific clinical protocols | excluded if currently engaged in medication for OUD (MOUD) treatment or opioid-requiring pain management, a participant in a substance use intervention study, medically or psychiatrically unstable, or a prisoner |
| Facilitating HIV/AIDS and HIV testing literacy for emergency department patients: a randomized, controlled, trial | Roland C. Merchant, Tao Liu, Melissa A. Clark and Michael P. Carey | 2018 | BMC Emergency Medicine | Manuscript | Investigate if videos and pictorial brochures might be equivalent methods of delivering HIV/AIDS and HIV testing information to emergency departments patients | Health equity/social EM | Primary enrollment | No, only ED | RCT | Yes | English; Spanish; Other: 2/4 sites were English only, the other 2/4 were Spanish only | No | 18-64-years-old | positive baseline rapid HIV test, enrolled in a conflicting HIV study, cannot provide informed consent and cannot participate, plan to leave in the US for 12 months |
| Reach out behavioral intervention for hypertension initiated in the emergency department connecting multiple health systems: study protocol for a randomized control trial | William J. Meurer, Mackenzie Dinh, Kelley M. Kidwell, Adam Flood, Emily Champoux, Candace Whitfield, Deborah Trimble, Joan Cowdery, Dominic Borgialli, Sacha Montas, Rebecca Cunningham, Lorraine R. Buis, Devin Brown, and Lesli Skolarus | 2020 | Trials | Manuscript | Studies if electronic health records in conjunction with mobile health behavior interventions can be leveraged to identify hypertensive patients to impact otherwise unreached populations | Health equity/social EM | Primary enrollment | No, only ED | RCT; Protocol | Yes | English | No | age of 18 or greater, at least one BP with SBP >160 mmHG or DBP>100 mmHG, have a cell phone with text-messaging capability and willingness to send and receive texts, likely to be discharged from the ED | prisoner, pregnancy, pre-existing condition making 1-year follow-up unlikely, terminal illness with death expected within 90 days, current use of three or more antihypertensive agents, patients with other serious medical conditions that prevent self-monitoring of BP, critical illness with placement in resuscitation bay, dementia/cognitive impairment |
| A randomized controlled trial testing the effectiveness of a paramedic-delivered care transitions intervention to reduce emergency department revisits | Ranran Mi, Matthew M. Hollander, Courtney M. C. Jones, Eva H. DuGoff, Thomas V. Caprio, Jeremy T. Cushman, Amy J. H. Kind, Michael Lohmeier and Manish N. Shah | 2018 | BMC Geriatrics | Manuscript | Test the effectiveness of the paramedic-delivered, modified care transition intervention program using a randomized controlled trial design. | Other clinical study | Primary enrollment | No, only ED | RCT | Yes | English | No | age 60 years or older, reside in Dane County, Wisconsin or Monroe County, New York, and have a primary care physician affiliated with UWHealth or UR Medicine, live in independent home dwellings, operationalized as not living in skilled nursing facilities or assisted living facilities, have a working telephone, and have the ability to provide informed consent (or have a legally authorized representative provide consent) | participated in the study previously; are actively enrolled in hospice, a transitions program, or an intensive care management program; are homeless; present for a behavioral health problem; or are severely ill, as defined by an Emergency Severity Index category of 1 |
| Brain Barriers: Elucidating Complex LanguageBased Barriersto Neurological Care in LimitedEnglish Proficiency, Refugee, and Immigrant Populations | Alexandra Miner | 2022 | Neurology | Abstract | To elucidate specific patient, provider, and resource-based variables that act as barriers to neurological care in limited-English proficiency communities | Health equity/social EM | Primary enrollment | Other: free clinic and outpatient neurology clinic | Cross sectional study; Qualitative | No | Spanish; Portuguese ; French; Creole; Chinese (including Mandarin); Arabic; Vietnamese ; Korean; Russian | Does not say | LEP patients | non- neurological chief complaints and diagnoses |
| Paramedic detection of large vessel occlusions using mnihss: A prospective cohort pilot study | Mulkerin, WilliamFrancisco, JonathanLima, Brandon Spokoyny, Ilana Gilbert, GregoryBrown, IanBernier, Eric Niknam, Kian Govindarajan, Prasanth | 2019 | INTERNATIONAL STROKE CONFERENCE | Abstract | To test the performance of modified NIHSS (mNIHSS) for the detection of large vessel occlusion when  used by paramedics | Other clinical study | Primary enrollment | No, only ED | Cohort study | No | English | No | over the age of 18 years, with acute neurological deficit consistent with suspected acute stroke | vulnerable patients |
| Single Dose Aminoglycosides for Acute Uncomplicated Cystitis in the Emergency Department Setting | Jacob Sieger, DO, Vincent Peyko, Pharm | 2023 | Clinical Trials website | Manuscript | To determine if gentamicin is as effective treatment of acute uncomplicated cystitis (urinary tract infection) using aminoglycosides versus the current standards of care | Other clinical study | Primary enrollment | No, only ED | RCT; Clinical trial; Protocol | Yes | Did not specify languages recruited | No | 18 years of age, woman, pre-menopausal, non-pregnant with clinical signs of urinary tract infection and nitrite positive urine | Contraindications include hypersensitivity to gentamicin or other aminoglycosides, Patients younger than 18 years of age, Pregnant or post-menopausal patients, Patients without symptoms of UTI, nitrite negative urine, or any patient with working diagnosis of complicated UTI such as pyelonephritis or any patient catheterized, ANY history of pre-existing renal impairment, Patient that has any listed or reports taking ANY nephrotoxic or wit drug-drug interaction medication in the past 7 days, ANY history of hearing loss, tinnitus, or vertigo; ANY patient that has received general anesthesia or neuromuscular blockade in past 7 days, Patients with ANY history of neuromuscular disorders, Patients with ANY history of thrombocytopenia, Patient that has any listed or reports taking ANY anticoagulants in the past 7 days |
| Randomized controlled pilot study of an educational video plus telecare for the early outpatient management of musculoskeletal pain among older emergency department patients | Timothy F. Platts-Mills, Allison G. Hollowell, Gary F. Burke, Sheryl Zimmerman, Joseph A. Dayaa, Benjamin R. Quigley, Montika Bush, Morris Weinberger and Mark A. Weaver | 2018 | Trials | Manuscript | Pilot study of an educational video about pain management with and without follow-up telephone support for older adults presenting to the ED with musculoskeletal pain | Other clinical study | Primary enrollment | No, only ED | RCT | Yes | English | No | aged 50 years and older presenting to the ED with musculoskeletal pain | complaining of head, chest, or abdominal pain and patients with pain suspected to be due to infection, ischemia, or another non-musculoskeletal problem, such as a kidney stone |
| Brief educational video plus telecare to enhance recovery for older emergency department patients with acute musculoskeletal pain: study protocol for the BETTER randomized controlled trial | Timothy F. Platts-Mills, Samuel A. McLean, Morris Weinberger Sally C. Stearns, Montika Bush, Brittni B. Teresi, Karen Hurka-Richardson, Kurt Kroenke, Robert D. Kerns, Mark A. Weaver and Francis J. Keefe | 2020 | Trials | Manuscript | Compares two versions of an intervention to usual care for preventing the transition from acute to chronic musculoskeletal pain among older adults in the emergency department | Other clinical study | Primary enrollment | No, only ED | RCT; Protocol | Yes | English | No | 50 years and older presenting to the ED with acute MSK pain | have been admitted to the hospital in the past 30 days, have pain due to self-injury, have an ESI of 1, are a prisoner, or have a diagnosis of somatoform disorder, schizophrenia, dementia, or bipolar disorder |
| Implementation of strength-based case management for opioid-dependent patients presenting in medical emergency departments: rationale and study design of a randomized trial | Amber Regis, Sarah E. Meyers-Ohki, Sarah E. Mennenga, Peter P. Greco, Richard Glisker, Rhonda Kolaric, Ryan P. McCormack, Richard C. Rapp and Michael P. Bogenschutz | 2020 | Trials | Manuscript | Investigate whether strength-based case management is an effective model for linking opioid-dependent ED patients with addiction treatment and pharmacotherapy. | Health equity/social EM | Primary enrollment | No, only ED | RCT; Protocol | Yes | English | No | 18 years of age or older, registered as a patient in the ED during screening hours, endorsed at least three opioid-dependence criteria by self-report on the DSM-IV checklist, and self-reported misuse of opioids within the 30 days before screening | inability to participate due to emergency treatment, significant impairment of cognition or judgment rendering the person incapable of informed consent, status as a prisoner or in police custody, current engagement in substance use disorder treatment, inability to provide two reliable locators, unavailability for follow-up, prior participation in the current study, or current participation in a research study related to substance use |
| Hearing Impairment, Strategies, and Outcomes in Emergency Departments | Joshua Chodosh | 2019 | Clinical Trials.gov | Abstract | Establish the feasibility of screening for hearing loss in the ED | Other clinical study | Primary enrollment | No, only ED | RCT; Protocol | Yes | English; Spanish | No | 60 Years and older, ESI 4-5, Hearing Handicap Impairment Evaluation Screen greater than or equal to 24, Capacity to consent to participate in research | Inability to consent to participate in research, ESI 1-3, Hearing Handicap Impairment Evaluation Screen less than 24 |
| Declared impact of the US President's statements and campaign statements on Latino populations' perceptions of safety and emergency care access | Robert M. Rodriguez, Jesus R. Torres, Jennifer Sun, Harrison Alter, Carolina Ornelas, Mayra Cruz, Leah Fraimow-Wong, Alexis Aleman, Luis M. Lovato, Angela Wong, Breena Taira | 2019 | PLOS ONE | Manuscript | To assess how President Trump's statements relate to undocumented Latino immigrants‚Äô and Latino legal residents/citizens‚Äô perceptions of safety and their presentations for emergency care | Health equity/social EM | Primary enrollment | No, only ED | Cross sectional study | No | English; Spanish; Portuguese ; French; Creole; Chinese (including Mandarin); Arabic; Vietnamese ; Korean; Russian | No | adult patients | 1) trauma; 2) transfer from another facility; 3) inability to participate in an interview because of intoxication, altered mental status or critical illness; 4) incarceration; and 5) on psychiatric hold |
| Validation of a 5-item tool to measure patient assessment of clinician compassion in the emergency department | Praveen Sabapathi, Michael B. Roberts, Brian M. Fuller, Michael A. Puskarich, Christopher W. Jones, J. Hope Kilgannon, Valerie Braz, Christina Creel-Bulos, Nathaniel Scott, Kristina L. Tester, Anthony Mazzarelli, Stephen Trzeciak, and Brian W. Roberts | 2019 | BMC Emergency Medicine | Manuscript | To test if the 5-item compassion measure (a tool previously validated in the outpatient setting to measure patient assessment of clinician compassion) is a valid and reliable tool to quantify a distinct construct (i.e. clinical compassion) among patients evaluated in the emergency department | Other clinical study | Primary enrollment | No, only ED | Cross sectional study | No | English | No | age>18, who were evaluated in the EDs of the participating institutions | unable to answer the survey questions |
| Data from emergency medicine palliative care access (EMPallA): a randomized controlled trial comparing the effectiveness of specialty outpatient versus telephonic palliative care of older adults with advanced illness presenting to the emergency department | Abigail M. Schmucker, Mara Flannery, Jeanne Cho, Keith S. Goldfeld, Corita Grudzen, and The EMPallA Investigators | 2021 | BMC Emergency Medicine | Manuscript | Aims to: 1) report baseline demographic and quality of life (QOL) data for the EMPallA cohort, 2) identify the association between illness type and baseline QOL while controlling for other factors, and 3) explore baseline relationships between illness type, symptom burden, and loneliness | Other clinical study | Primary enrollment | No, only ED | RCT | Yes | English; Spanish | No | adults aged 50 years or older, who have advanced cancer (metastatic solid tumor) or end-stage organ failure | dementia documented in the electronic health record (EHR) problem list, having received hospice services or two or more palliative care visits in the last six months, residing in a long-term care facility, or being admitted to the hospital for more than 48 h post ED encounter |
| Patients' willingness to share digital health and non-health data for research: a cross-sectional study | Emily Seltzer, Jesse Goldshear, Sharath Chandra Guntuku , Dave Grande, David A. Asch, Elissa V. Klinger and Raina M. Merchant | 2019 | BMC Medical Informatics and Decision Making | Manuscript | Patients perception of the data they generate online and its relatedness to health, their willingness to share data for research, and their preferences regarding data use | Other clinical study | Primary enrollment | No, only ED | Cross sectional study | No | English | No | Patients seeking care in a high volume, urban, academic Emergency Department from July to November 2017 were approached by research assistants for study participation | < 18 years old or High acuity and Trauma Level I |
| Feasibility and Short-Term Effects of a Multi-Component Emergency Department Blood Pressure Intervention: A Pilot Randomized Trial | Journal of the American Heart Association | 2022 | Andrew J. Spieker, PhD; Lyndsay A. Nelson, PhD; Russell L. Rothman, MD, MPP; Christianne L. Roumie, MD, MPH; Sunil Kripalani, MD, MSc; Joseph Coco, MS; Daniel Fabbri, PhD; Phillip Levy, MD, MPH; Sean P. Collins, MD, MSc; Tommy Wang, MD; Dandan Liu, PhD; Candace D. McNaughton | Manuscript | Compare short-term blood pressure measures between the Vanderbilt Emergency Room Bundle intervention and usual care plus education | Other clinical study | Primary enrollment | No, only ED | RCT | Yes | English | No | age 21 to 84 years (at the time of the study, the National Institutes of Health defined adult as >21 years of age, enrollment within 6 hours of initial ED evaluation | patients of age >85 years were excluded because of concerns of safety in a more frail population, an established primary care provider with a clinic visit within the previous 365 days, at least 1 antihypertensive medication prescription, first systolic blood pressure (SBP) measured upon ED arrival of at least 140 mm Hg measured during routine care, anticipated discharge from the ED, willingness and ability to receive text messages and return for follow-up visits, receiving hemodialysis, had symptoms preventing oral intake or affecting BP (eg, vomiting, acute alcohol withdrawal, known pregnancy), or had received vasoactive or anti-hypertensive medication in between ED presentation and enrollment |
| Feasibility and Acceptability of a Text Message-Based Intervention to Reduce Overuse of Alcohol in Emergency Department Patients: Controlled Proof-of-Concept Trial | Elizabeth Burner, Mark Zhang, Sophie Terp, Kelsey Ford Bench, Joshua Lee, Chun Nok Lam, Jesus R Torres, Michael Menchine and Sanjay Arora | 2020 | Journal of Medical Internet Research | Manuscript | To assess the feasibility of and patient satisfaction with a text-based mHealth extension of an ED screening program to reduce risky alcohol use in low-income, urban patients | Health equity/social EM | Primary enrollment | No, only ED | Clinical trial; Other: quasiexperimental trial | Yes | English; Spanish | No | Adult ED patients | precluded by a clinical condition, language barrier (any language other than English or Spanish), or other inability to verbally consent to screening |
| Implementation of Multidisciplinary Assessments for Geriatric Patients in an ED Observation Unit | Lauren Southerland | 2019 | Clinical Trials.gov | Abstract | Implement and investigate an ED protocol to screen older adults for these syndromes and address them with multidisciplinary geriatric assessments in an ED Observation Unit | Other clinical study | Primary enrollment | No, only ED | Cohort study; Clinical trial; Protocol | Yes | English | No | Adult patients >= 65 years old assigned to the ED Observation Unit for care | Need for inpatient care at the time of initial ED disposition, acute psychiatric issues requiring stabilization by psychiatry team, prisoners, unable to consent and no legally authorized representative available |
| Implementing a Social Determinants Screening and Referral Infrastructure During Routine Emergency Department Visits, Utah, 2017-2018 | Andrea S. Wallace, Brenda Luther, Jia-Wen Guo, Ching-Yu Wang, Shawna Sisler, Bob Wong | 2020 | PREVENTING CHRONIC DISEASE PUBLIC HEALTH RESEARCH, PRACTICE, AND POLICY | Manuscript | Developed and evaluated a process for identifying social needs among emergency department patients, for facilitating access to community-based resources, and for integrating clinical and community-based data | Health equity/social EM | Primary enrollment | No, only ED | Cross sectional study | Yes | English | No | positive social needs screening by registration staff | did not speak english or spanish |
| Qualitative study of patient experiences and care observations during agitation events in the emergency department: implications for systems-based practice | Ambrose H Wong, Jessica M Ray, Christopher Eixenberger, Lauren J Crispino, John B Parker, Alana Rosenberg, Leah Robinson, Caitlin McVaney, Joanne DeSanto Iennaco,Steven L Bernstein, Kimberly A Yonkers, Anthony J Pavlo | 2022 | BMJ Open | Manuscript | To use a systems-based approach, which considers the larger context and system of healthcare delivery, to identify sociotechnical, structural, and process-related factors leading to agitation events and physical restraint use in the ED | Other clinical study | Primary enrollment | No, only ED | Qualitative | No | Did not specify languages recruited | No | agitation events during adult patient (>18 years of age) visits in the ED |  |
| Association Between Perceived Discrimination and Emergency Department Use Among Safety-Net Patients in the Southwestern United States | Kimberly R. Enard, PhD, Lucinda Nevarez, PhD, LMSW, Deborah M. Ganelin, MHA | 2018 | Southern Medical Journal | Manuscript | Describe the prevalence and nature of perceived discrimination or perceived unfair treatment, and to examine the association of PD/PUT with healthcare utilization among adult safety-net patients | Health equity/social EM | Primary enrollment | No, only ED | Cross sectional study | No | English; Spanish | No | 18 to 64 years old, uninsured or covered by Medicaid, participated in a patient navigation program, ESI 3-5 | judgment of the navigators (eg, patients appeared to be unapproachable or unstable) |
| Design and patient characteristics of the randomized controlled trial TExT-MED+FANS A test of mHealth augmented social support added to a patient-focused text-messaging intervention for emergency department patients with poorly controlled diabetes | Elizabeth Burner, Janisse Mercado, Antonio, Hernandez-Saenz, Anne Peters, Wendy Mack, Lourdes Baezconde-Garbanati, Sanjay Arora, Shinyi Wu | 2019 | Contemporary Clinical Trials | Manuscript | Describe protocol and enrollment for RCT mHealth social support tool for patients with poorly controlled diabetes | Health equity/social EM | Primary enrollment | No, only ED | RCT | Yes | English; Spanish | No | Age 18 or greater, Stable ownership of mobile phone, Able to send and receive text messages, A1C 8.5 or greater Identifies a support person who can be contacted within 2 weeks to enroll | Psychiatric involuntary hold, or in police custody, Altered mental status, Clinically unstable to consent and complete baseline assessment |
| Impact of Prior Trauma Exposure on the Development of PTSD Symptoms after Suspected Acute Coronary Syndrome | Justin Young, Adam Schweber, Jennifer A. Sumner, Bernard P. Chang, Talea Cornelius, Ian M. Kronish | 2021 | General Hospital Psychiatry | Manuscript | To determine the association between PTSD symptoms due to prior trauma and prior trauma type with PTSD symptoms after suspected acute coronary syndrome | Other clinical study | Secondary analysis | No, only ED | Cohort study | No | English; Spanish | No | provisional diagnosis of "probable ACS" by an ED physician | ST-segment elevations upon presentation, unable to provide informed consent or complete study questionnaires from cognitive or functional impairment, needed immediate psychiatric intervention, had terminal non-cardiovascular illness |
| Feasibility of a Brief Intervention to Facilitate Advance Care Planning Conversations for Patients with Life-Limiting Illness in the Emergency Department | Sarah E. Pajka, BA, Mohammad Adrian Hasdianda, MD, MSc, MMSc, Naomi George, MD, Rebecca Sudore, MD, Mara A. Schonberg, MD, MPH, Edward Bernstein, MD, James A. Tulsky, MD, Susan D. Block, MD, and Kei Ouchi, MD, MPH | 2021 | JOURNAL OF PALLIATIVE MEDICINE | Manuscript | To determine the feasibility of an ED-based, brief negotiated interview (BNI) to stimulate ACP conversations among seriously ill older adults | Other clinical study | Primary enrollment | No, only ED | Cohort study; Other: prospective, one-arm, pre/postintervention study | Yes | English | No | 65 years and older with one or more serious illness | clearly documented goals for medical care or with a medical order for life-sustaining treatment in the electronic medical record |
| Minimizing Variability in Interpretation Modality Among Spanish-Speaking Patients With Limited English Proficiency | Seiichi Villalona, Christian Jeannot, Mery Yanez Yuncosa, W. Alex Webb, Carol Boxtha, and Jason W. Wilson | 2020 | Hispanic Health Care International | Manuscript | Understand how emergency department (ED) staff went about treating patients with LEP and examine the impact of consistent interpretation modality on overall patient satisfaction and comprehension | Health equity/social EM | Primary enrollment | No, only ED | Cross sectional study | No | Spanish | No | at least 18 years of age, identified Spanish as their preferred language of communication, and were triaged with a low emergency severity index | any past medical history of psychiatric/mental health conditions that would interfere with the informed consent process |
| Use of a Rapid Diagnostic for Chlamydia trachomatis and Neisseria gonorrhoeae for Women in the Emergency Department Can Improve Clinical Management: Report of a Randomized Clinical Trial | Charlotte A. Gaydos, MA, DrPH; Michele-Corinne Ako, BS; Mitra Lewis, BS; Yu-Hsiang Hsieh, PhD; Richard E. Rothman, MD, PhD; Andrea F. Dugas, MD | 2019 | Annals of Emergency Medicine | Manuscript | To assess rapid C trachomatis and N gonorrhoeae testing on overtreatment and undertreatment of women evaluated for C trachomatis and N gonorrhoeae | Other clinical study | Primary enrollment | No, only ED | RCT | Yes | English | No | Women aged 18 to 50 years and undergoing pelvic examination including C trachomatis and N gonorrhoeae testing as part of their ED standard of care | if they were unable, to provide informed consent, and if they had a known pregnancy |
| Trajectories of posttraumatic stress in patients with confirmed and rule-out acute coronary syndrome | Laura Meli, Jeffrey Birk, Donald Edmondson, George A. Bonanno | 2020 | General Hospital Psychiatry | Manuscript | Many patients evaluated in the emergency department (ED) for acute coronary syndrome (ACS) develop post-traumatic stress symptoms (PTSS), but little is known about symptom trajectories over time | Other clinical study | Secondary analysis | No, only ED | Cohort study | No | English; Spanish | No | the first 1000 English- and Spanish-speaking patients enrolled from November 2013 to February 2016 in the REACH study | STEMI due to hospital emergency department fast track procedures for catheterization, inability to follow the protocol (due to dementia or substance abuse), need for immediate psychiatric intervention, and lack of availability for follow-up |
| The HEART Pathway Randomized Controlled Trial One-year Outcomes | Jason P. Stopyra, Robert F. Riley, Brian C. Hiestand, Gregory B. Russell, James W. Hoekstra, Cedric W. Lefebvre, Bret A. Nicks, David M. Cline, Kim L. Askew, Stephanie B. Elliott, David M. Herrington, Gregory L. Burke, Chadwick D. Miller, Simon A. Mahler, | 2019 | Academic Emergency Medicine | Manuscript | Determine the impact of the HEART Pathway on health care utilization and safety outcomes at 1 year in patients with acute chest pain | Other clinical study | Secondary analysis | No, only ED | RCT; Clinical trial | Yes | English | No | at least 21 years old and had any symptoms that could suggest possible ACS, provider ordered an electrocardiogram and troponin for the evaluation of ACS | new ST-segment elevation > 1 mm; hypotension; life expectancy < 1 year; a noncardiac medical, surgical, or psychiatric illness determined by the provider to  require admission; prior enrollment; non‚ÄìEnglish speaking; and incapacity or unwillingness to consent |
| Bringing Close Others to the Emergency Department for an Acute Coronary Event is Associated with Increased Patient Perception of Threat | Talea Cornelius, Laura Meli, Katherine R. Thorson, Bernard P. Chang, Donald Edmondson, Tessa V. West | 2019 | General Hospital Psychiatry | Manuscript | Tested the unexplored role that close others play in patients' experience of threat during evaluation for acute coronary syndrome in the ED, as well as the indirect effect of close others on ACS-induced PTSD | Other clinical study | Secondary analysis | No, only ED | Cohort study | No | English; Spanish | No | Hospital ED for evaluation for suspected ACS (i.e., non-ST elevation myocardial infarction or unstable angina) |  |
| Who is informed of trauma informed care? Patients' primary language and comprehensiveness of initial trauma assessment | Souma Kundu, MPH, Todd W. Costantini, MD, Jay J. Doucet, MD, and Allison E. Berndtson, MD | 2022 | Trauma Acute Care Surgery | Manuscript | Hypothesized that there would be limited use of certified medical interpretation during major trauma resuscitations, less comprehensive assessments, and less empathetic communication for Spanish-speaking patients with limited English proficiency compared with English-speaking patients | Health equity/social EM | Primary enrollment | No, only ED | Cohort study | No | English; Spanish | No | alert and medically stable during the initial evaluation with a GCS score of >13 | videos with poor quality and patients who were younger than 18 years, incarcerated, had injuries that prevented them from verbally communicating, or had major concerns with the "Airway, Breathing, or Circulation" steps of ATLS requiring emergency interventions before completion of the primary and secondary survey |
| Emergency department-provided home blood pressure devices can help detect undiagnosed hypertension | Elizabeth M. Goldberg, Taneisha Wilson, Bianca Jambhekar, Sarah J. Marks, Michael Boyajian, Roland C. Merchant | 2019 | High Blood Pressure & Cardiovascular Prevention | Manuscript | Home blood pressure monitoring is used routinely in outpatient settings, yet its utility after the ED visit for those with elevated BP in  the ED is unclear | Other clinical study | Primary enrollment | No, only ED | Cohort study | No | English | No | at least 18 years-old and have a triage BP >120 mmHg systolic and/or >80 mmHg diastolic | known HTN diagnosis or were taking antihypertensive medication, receiving dialysis, pregnant, prisoners, intoxicated, admitted, or in infectious disease isolation |
| Can Older Adult Emergency Department Patients Successfully Use the Apple Watch to Monitor Health? | Kevin Chen, MD, Hari Dandapani, BS, Kate M. Guthrie, PhD, Elizabeth Goldberg, MD, ScM | 2013 | Rhode Island Medical Journal | Manuscript | Determine usability of the Apple Watch in older adult ED patients after a fall | Other clinical study | Primary enrollment | No, only ED | Cohort study | Yes | English | No | community-dwelling, fall not due to syncope or external force or acute serious illness, and likely to be discharged to home/assisted living/rehabilitation at completion of ED visit | altered mental status, injuries that prevented mobilization, allergies to any device component, and those who were unable to wear the Apple Watch at home or had a diagnosis of advanced cancer or those in hospice care |
| Effectiveness of a Care Transitions Intervention for Older Adults Discharged Home from the Emergency Department: A Randomized Controlled Trial | Gwen C. Jacobsohn PhD, MA, Courtney M. C. Jones PhD, MPH, Rebecca K. Green MPH, Amy L. Cochran PhD, Thomas V. Caprio MD, MPH, MS, Jeremy T. Cushman MD, MS, Amy J. H. Kind MD, PhD, Michael Lohmeier MD, Ranran Mi MA, Manish N. Shah MD, MPH | 2021 | Society for Academic Emergency Medici | Manuscript | Tested the effectiveness of the Care Transitions Intervention with community-dwelling older adult ED patients, hypothesizing that it would reduce revisits and increase performance of self-management behaviors during the 30 days following discharge | Other clinical study | Primary enrollment | No, only ED | RCT | Yes | English | No | at least 60 years of age; resided in either Dane County, Wisconsin, or Monroe County, New York; had a primary care provider affiliated with either health system; had a working telephone; and were discharged from the ED to a community residence within 24 h of arrival | visually or hearing impaired; did not have a permanent residence; were actively enrolled in hospice, a transitions program, or a care management program; presented with a primary behavioral or psychiatric problem; or had an ESI category of 1 |
| Effectiveness and Cost of Recruiting Participants to a Research Registry Using an Emergency Department Research Associate Program | Carrie Dykes, Joseph Glick, Beau Abar and Ann Dozier | 2020 | Clinical and Translational Science | Manuscript | Is registry recruitment in the ED feasible and how does that compare with passive methods | Other clinical study | Primary enrollment | No, only ED | Cross sectional study | No | English; Spanish; Portuguese ; French; Creole; Chinese (including Mandarin); Arabic; Vietnamese ; Korean; Russian; Other: non-English speaking included if a translator was present | No | local mailing address, email address, or phone number | previously registered, or inability to give informed consent |
| The active role of interpreters in medical discourse: An observational study in emergency medicine | Natalie C. Bendaa, Ann M. Bisantz, Rebecca L. Butler, Rollin J. Fairbanks, Jeff Higginbotham | 2022 | Patient Education and Counseling | Manuscript | Observe English proficient and LEP patients throughout their stay in the ED to understand what strategies are used for communicating beyond verbatim translation and how personnel affect these strategies | Health equity/social EM | Primary enrollment | No, only ED | Cross sectional study | No | English; Spanish | No | over the age of 18 | unable to provide consent |
| Barriers to healthcare after the Affordable Care Act: A qualitative study of Los Angeles safety net patients' experiences with insurance and healthcare | Sonali Saluja, Danny McCormick, Michael Cousineau, Janina Morrison and Michael Hochma | 2019 | Health Equity | Abstract | Understand about barriers toobtaining insurance and accessing healthcare in Los Angeles in the post-ACA era | Health equity/social EM | Primary enrollment | No, only ED | Qualitative | No | English; Spanish | No | ages 18-64 years, who had either MediCal, MyHealthLA, or were unsinsured | too ill to interview |
| Exploring the Components of an Efficacious Computer Brief Intervention for Reducing Marijuana Use among Adults in the Emergency Department | Rebecca Waller, Erin E. Bonar, Anne C. Fernandez, Maureen A. Walton, Stephen T. Chermack, Rebecca M. Cunningham, Frederic C. Blow | 2019 | Journal of Substance Abuse Treatment | Manuscript | To examine the efficacious components of a computer-delivered brief intervention (CBI) for reducing marijuana use among adults presenting to a low-income urban emergency department (ED), which a prior report found to decrease marijuana use at a 6-month follow-up | Health equity/social EM | Secondary analysis | No, only ED | RCT | Yes | English | No | Patients aged 18-60 years presenting to the ED | inability to consent (e.g., psychosis, medically unstable, police custody), seeking care for suicidal ideation or acute sexual assault, or severe hearing or visual impairment |
| Health Care Utilization by Women Sexual Assault Survivors after Emergency Care: Results of a Multisite Prospective Study | Nicole A. Short, PhD, Megan Lechner, MSN, Benjamin S. McLean, Andrew Tungate, PhD, Jenny Black, BSN, Jennie Buchanan, MD, Rhiannon Reese, MA, Jeffrey Ho, MD, Gordon Reed, MD, Melissa Platt, MD, Ralph Riviello, MD, Catherine Rossi, MSN, Patricia Nouhan, MD, Carolyn Phillips, MD, Sandra L. Martin, PhD, Israel Liberzon,  MD, Sheila A.M. Rauch, PhD, Kenneth Bollen, PhD, Ronald C. Kessler, MD, Samuel A. McLean, MD | 2021 | Depression Anxiety | Manuscript | Evaluated health outcomes and service utilization among women in the six weeks after sexual assault | Other clinical study | Primary enrollment | No, only ED | Cohort study | No | English | No | at least 18 years of age and presenting for emergency care within 72 hours of sexual assault | inability to provide informed consent, pregnancy (due to varying trajectories of mood, pain, and sleep across pregnancy and the postpartum period), planning on living with the assailant after the assault (due to likelihood of repeat traumatization and thus differing patterns of PTSS and other outcomes), fracture (as  fracture is rare after SA and can influence pain outcomes, an important aim of the study), hospital admission, no telephone access, no mailing  address, unwilling to provide blood sample, incarceration, and inability in opinion of study  staff to be able to follow the study protocol |
| Using a novel rapid viral test to improve triage of emergency department patients with acute respiratory illness during flu season | Courtney J. Pedersen, Daniel T. Rogan, Samuel Yang, James V. Quinn | 2018 | Journal of Clinical Virology | Manuscript | To investigate the value of POC-PCR in rapidly identifying RSV and influenza in the setting of ED triage | Other clinical study | Primary enrollment | No, only ED | Cohort study | Yes | English; Spanish | Yes | acute resp illness symptoms - measured fever at home or in ED > 38C and a cough, sore throat, or rhinorrhea with a duration of symptoms > 12 h and < 1 week; all ages were eligible | arriving by ambulance or who had already received oseltamivir for their current illness |
| Subject Retention in Prehospital Stroke Research Using a Telephone-Based Physician-Investigator Driven Enrollment Method | Bryant J. Rosell, Kristina Shkirkova, Jeffrey L. Saver, David S. Liebeskind, Sidney Starkman May Kim-Tensera, Marc Eckstein, Latisha Sharma, Robin Conwit, Scott Hamilton, Nerses Sanossian | 2019 | Cerebrovascular Diseases Extra | Manuscript | Describe rates of subject retention in a prehospital trial of acute stroke therapy | Other clinical study | Secondary analysis | Other: prehospital | RCT; Clinical trial | Yes | English; Spanish | No | Consent conversations are conducted in a language in which the consent provider is fluent either English or Spanish |  |
| Predictors of empirically derived substance use patterns among sexual minority groups presenting at an emergency department | Brooke J. Arterberry, Alan K. Davis, Maureen A. Walton, Erin E. Bonar, Rebecca M. Cunninghamd, Frederic C. Blow | 2019 | Addictive Behaviors | Manuscript | Evaluate substance use patterns among lesbian gay bisexual individuals using latent class analysis | Health equity/social EM | Secondary analysis | No, only ED | RCT | Yes | English | No | adult patients (ages 21 to 65) presenting to the ED who identify as lesbian, gay, bisexual | presenting to the ED with altered cognition that would impact ability to consent (e.g., active psychosis, psychiatric admission, intoxicated, unconscious) or for reasons that would require intensive social work intervention during the ED visit (e.g., suicidal ideation, sexual assault), visual/hearing impairment, violent, participation refusal, blood alcohol content over 0.10, and currently engaging in alcohol treatment |
| Prevalence of Homelessness by Gender in an Emergency Department Population in Pennsylvania | Brett J. Feldman, MSPAS, PA-C; Alexandra M. Craen, MD; Joshua Enyart, DO; Timothy Batchelor, BS; Timothy J. Friel, MD; Stephen W. Dusza, DrPH; Marna Rayl Greenberg, DO, MPH | 2018 | The Journal of the American Osteopathic Association | Manuscript | To assess the prevalence of homelessness by gender in 3 EDs in Pennsylvania | Health equity/social EM | Primary enrollment | No, only ED | Cross sectional study | No | English | No | registered patients in the ED, be aged at least 18 years, speak English, have the capacity to answer survey questions, be willing to participate, and not have taken the survey before | critically ill |
| Frequency of primary neck pain in mild traumatic brain injury/concussion patients | Jeffrey A. King, DC, MS, Michael A. McCrea, PhD, Lindsay D. Nelson, PhD | 2020 | Archives of Physical Medicine and Rehabilitation | Manuscript | To determine (1) the frequency of neck pain overall and relative to other symptoms in patients presenting to a level I trauma center emergency department with mild traumatic brain injury and (2) the predictors of primary neck pain in this population | Other clinical study | Primary enrollment | No, only ED | Cohort study | No | English | No | 18-45 years old, loss of consciousness <30 minutes, posttraumatic amnesia <24 hours, no acute intracranial findings on brain imaging (if available), and ability to present for the initial assessment within 72 hours of injury | an injury that precluded participation in the study protocol (eg, hand injury that prevented use of a computer mouse), current diagnosis of a psychotic disorder, history or clinical suspicion of other conditions (eg, epilepsy, stroke, dementia) known to cause cognitive dysfunction, and history of moderate or severe traumatic brain injury |
| Posttraumatic Stress Disorder in Patients Who Rule Out Versus Rule In for Acute Coronary Syndrome | Ian M. Kronish, Donald Edmondson, Nathalie Moise, Bernard P. Chang, Ying Wei, David Lopez Veneros, William Whang | 2018 | General Hospital Psychiatry | Manuscript | To compare the risk of developing PTSD among patients who rule out versus rule in for ACS | Other clinical study | Secondary analysis | No, only ED | Cohort study | No | English; Spanish | No | given a provisional diagnosis of "probable ACS" by their treating ED physician during the initial evaluation of chest pain | ST-segment elevations on their electrocardiograms when presenting to the ED as these patients were immediately sent to the cardiac catheterization laboratory upon arrival and were unavailable for consent in the ED, unable to provide informed consent or complete study questionnaires due to cognitive impairment, need of immediate psychiatric intervention, or terminal illness |
| Patients' Response to an Emergency Department-Based HIV Testing Program and Perception of Their Friends' Attitudes on HIV Testing among Patients Seeking Care at an Urban Emergency Department in Baltimore, Maryland, USA | Cassie Wicken, Ama Avornu, Carl A. Latkin, Melissa A. Davey-Rothwell, Jim Kim, Raza Zaidi, Richard Rothman, Yu-Hsiang Hsieh | 2020 | Journal of Infection and Public Health | Manuscript | To understand the role of social network members and peer attitudes on emergency department patients‚ willingness to be tested for HIV | Health equity/social EM | Primary enrollment | No, only ED | Cross sectional study; Qualitative | Yes | English | No | age 18-65 years, able to give informed consent, not critically ill | Patients who were critically ill, unable to provide informed consent, not English-speaking, or prisoners were excluded |
| From Their Perspective: The Connection between Life Stressors and Health Care Service Use Patterns of Homeless Frequent Users of the Emergency Department | Megan Moore, Kelsey M. Conrick, Ashok Reddy, Ann Allen, and Craig Jaffe | 2019 | National Association of Social Workers | Manuscript | We present the views of a group of homeless frequent users of the ED about their service use and the influence of current and past stressors on their patterns of health care utilization. We then asses barriers and facilitators to care and service access to gain insight into methods for improving services to homeless patients. | Health equity/social EM | Primary enrollment | No, only ED | Qualitative | No | English | No | eligibility for or participation in the HUCM program, ability to communicate verbally | unable to provide consent |
| Integrating social determinants of health screening and referral during routine emergency department care: evaluation of reach and implementation challenges | Andrea S. Wallace, Brenda L. Luther, Shawna M. Sisler, Bob Wong and Jia-Wen Guo | 2021 | Implementation Science Communications | Manuscript | To implement universal social needs screening and community service referrals in an academic emergency department (ED), evaluating for feasibility, reach, and stakeholder perspectives | Health equity/social EM | Primary enrollment | No, only ED | Cross sectional study | Yes | English; Spanish | No | All patients being admitted to the ED, English/ Spanish speaking | Staff were asked to use their discretion and omit patients with cognitive impairment, trauma, language other than English or Spanish, or residents of skilled nursing facilities |
| Mobile device ownership among emergency department patients | Eugene Kim, John Torous, Steven Horng, Anne V. Grossestreuer, Jorge Rodriguez, Terrance Lee, Larry A. Nathanson | 2019 | International Journal of Medical Informatics | Manuscript | To determine characteristics of mobile device ownership among Emergency Department patients, patients' feelings regarding their use in healthcare, and desired functionality in mobile applications | Other clinical study | Primary enrollment | No, only ED | Cross sectional study | No | English | No | aged 18 and older | critical illness, had an altered mental status, or were otherwise unable to complete the survey |
| Patterns of Peritraumatic Threat Perceptions in Patients Evaluated for Suspected Acute Coronary Syndrome According to Prior and Current Posttraumatic Stress Symptoms | Deanna R. Zhu, Jacob Julian, Sung J.A. Lee, Anusorn Thanataveerat, Jennifer A. Sumner | 2018 | General Hospital Psychiatry | Manuscript | Investigated how threat perceptions during ACS evaluation in the emergency department (ED) and upon recall were associated with posttraumatic psychopathology burden due to prior trauma and the suspected ACS | Other clinical study | Secondary analysis | No, only ED | Cohort study | No | English; Spanish | No | Patients provisionally diagnosed with probable ACS by the treating ED physician | patients that required emergency transfer for cardiac catheterization, eemed unable to follow the protocol by the attending physician or the research coordinator due to mental impairment or active substance abuse |
| Intravenous peramivir vs oral oseltamivir in high-risk emergency department patients with influenza: Results from a pilot randomized controlled study | Yu-Hsiang Hsieh, Andrea F. Dugas, Frank LoVecchio, Breana McBryde, Erin P. Ricketts, Kathryn Saliba-Shaw, Richard E. Rothman | 2019 | Influenza and Other Respiratory Viruses | Manuscript | Compare outcomes of ED patients at high risk for influenza complications treated with IV peramivir vs oral oseltamivir | Other clinical study | Primary enrollment | No, only ED | RCT | Yes | English; Spanish; Other: 1/2 study site enrolled Spanish | No | 18 years or older; ED positive influenza test meeting the 2011 CDC criteria for antiviral treatment; with symptoms onset of less than 96 hours; able to provide informed consent; and expressed willingness to comply with all study procedures including follow-up requirements | unable or unwilling to provide informed consent; previously enrolled in the study during the current influenza season; unable to take oral medication; using any neuraminidase inhibitors within the past 7 days; known allergic reaction to neuraminidase inhibitors; pregnant or breastfeeding; and having end-stage renal disease, end stage liver disease, G6PD deficiency, or immunodeficiency |
| Commitments of intended drinking behavior change in latino emergency department patients using automated bilingual computerized alcohol screening and intervention | F.E. Vaca, F. Abujarad, J. Dziura, M. Pantalon, J. Reynolds, A. Hsaio, C. Field, M. Pantalon,G.  D'Onofrio | 2019 | Alcoholism: clinical and experimental research | Abstract | AutomatedBilingual Computerized Alcohol Screening and Intervention (AB-CASI) in an urban ED and commitment of intended behavior change among Latino patients | Health equity/social EM | Secondary analysis | No, only ED | RCT; Cross sectional study | Yes | English; Spanish | No | Latino patients | unable to provide consent |
| Persistent and Widespread Pain Among African Americans Six Weeks after MVC: Emergency Department-based Cohort Study | Francesca L. Beaudoin, MD, MS, PhD Wanting Zhai, ScM Roland C. Merchant, MD Melissa A. Clark, PhD Michael C. Kurz, MD Phyllis Hendry MD Robert A. Swor, DO David Peak, MD Claire Pearson, MD Robert Domeier, MD Christine Ortiz, MD Samuel A. McLean, MD | 2020 | Western Journal of Emergency Medicine | Manuscript | Evaluated the incidence and predictors of moderate to severe axial musculoskeletal pain and widespread pain six weeks after a MVC in a large cohort of Black adults | Other clinical study | Primary enrollment | No, only ED | Cohort study | No | English | No | patients 18-65 years old presented to the ED within 24 hours of an MVC and were discharged home after evaluation, non-Hispanic Black or African-American | admitted to the hospital, had any fractures other than phalangeal fractures, had more than four lacerations requiring sutures or a single laceration more than 20 centimeters in length, or had intracranial or spinal injuries, patients who were not alert and oriented, pregnant patients, inmates |
| The association between symptom onset characteristics and prehospital delay in women and men with acute coronary syndrome | Sahereh Mirzaei, Alana Steffen, Karen Vuckovic, Catherine Ryan, Ulf G Bronas, Jessica Zegre-Hemsey, Holli A DeVon | 2020 | European Journal of Cardiovascular Nursing | Manuscript | To determine if there was an association between gradual vs abrupt symptom onset and prehospital delay for patients with acute coronary syndrome and to examine the relationship between activities at symptom onset and gradual vs abrupt symptom onset | Other clinical study | Secondary analysis | No, only ED | Cohort study | No | English | No | >21 years old and arrived by private transportation or emergency medical services | exacerbation of heart failure (brain natriuretic peptide >500 pg/ml), transferred from a hemodialysis center, referred for evaluation of a dysrhythmia, and cognitive impairment |
| Prevalence of Emergency Department Social Risk and Social Needs | Melanie F. Molina, MD Caitlin N. Li, MD Emily C. Manchanda, MD Benjamin White, MD Mohammad K. Faridi, MPH Janice A. Espinola, MPH Henry Ashworth, MPH Gia Ciccolo, MPH Carlos A. Camargo Jr., MD, DrPH Margaret Samuels-Kalow, MD, MPhil, MSHP | 2020 | Western Journal of Emergency Medicine | Manuscript | To describe the relationship between social risk and social need, and identify factors associated with differential responses to social risk and social need questions | Health equity/social EM | Primary enrollment | No, only ED | Cross sectional study | No | English; Spanish | Yes | all newly arriving patients and parents of pediatric patients (<18 years of age) entering the treatment area | patients were inappropriate for enrollment, eg, intoxication or altered mental status to the degree of inhibiting decision-making capacity, or high medical acuity requiring immediate attention |
| Therapist and Computer-Based Brief Interventions for Drug Use within a Randomized Controlled Trial: Effects on Parallel Trajectories of Alcohol Use, Cannabis Use, and Anxiety Symptoms | Laura E. Drislane, Rebecca Waller, Meghan E. Martz, Erin E. Bonar, Maureen A. Walton, Stephen T. Chermack, & Frederic C. Blow | 2019 | Society for the Study of Addiction | Manuscript | Examine whether a therapist-delivered BI (TBI) or computer-guided BI (CBI) to address drug use, alcohol consumption (when relevant) and HIV risk behaviors, relative to enhanced usual care (EUC), was associated with reductions in parallel trajectories of alcohol use, cannabis use and anxiety symptoms | Health equity/social EM | Secondary analysis | No, only ED | RCT | Yes | English | No | aged 18-60 year | psychosis, medical instability, in police custody, seeking care for suicidal ideation or acute sexual assault, illiterate, severe hearing or visual impairment |
| Normal Saline and Lactated Ringer's have a Similar Effect on Quality of Recovery: A Randomized Controlled Trial | Andrew Friederich, BA; Natalie Martin, BA; Morgan B. Swanson, BS; Brett A. Faine, PharmD, MS; Nicholas M. Mohr, MD, MS | 2019 | Annals of Emergency Medicine | Manuscript | To test the hypothesis that balanced crystalloids improve quality of recovery more than normal saline solution (0.9% sodium chloride) in stable ED patients | Other clinical study | Primary enrollment | No, only ED | RCT | Yes | English | No | adult (18 to 100 years) ED patients presenting with one of the following complaints: nausea, vomiting or emesis, diarrhea, abdominal pain, dizziness, weakness, heat stroke or heat exhaustion, dehydration, fatigue, or volume depletion | pregnant, were prisoners, were undergoing current chemotherapy, had signs of jaundice, had already received greater than 250 mL of intravenous fluids, or were unable to provide informed consent |
| Resource Utilization Across the Continuum of HIV Care: an Emergency Department-based Cohort Study | Abia Abia, MD, Richard E. Rothman, MD, PhD, Amir M. Mohareb, MD, Grace Li Hsien Lim, MBBS, Anuj V. Patel, MD, Benjamin Bigelow, BS, Eili Y. Klein, PhD, Gai Cole, DrPH, MBA, Kelly A. Gebo, MD, MPH, Richard D. Moore, MD, MHS, Yu-Hsiang Hsieh, PhD | 2021 | American Journal of Emergency Medicine | Manuscript | To determine the healthcare resource utilization for people living with HIV (PLWH) presenting to the emergency department (ED) across the HIV Care Continuum | Health equity/social EM | Primary enrollment | No, only ED | Cohort study | No | English | No | record of a previous reactive HIV immunoassay, HIV RNA PCR assay, or previous HIV diagnosis in the medical record | age younger than 18 years, impaired cognition, and inability to consent due to acuity of their illness |
| Ability of Older Adults to Report Elder Abuse: An Emergency Department-Based Cross-Sectional Study | Natalie L. Richmond, BS, Sheryl Zimmerman, PhD, Bryce B. Reeve, PhD, Joseph A. Dayaa, BA, Mackenzie E. Davis, BA, Samantha B. Bowen, BA, John A. Iasiello, BA, Rachel Stemerman, BA, Rayad B. Shams, BS, Jason S. Haukoos, MD, MSc, Philip D. Sloane, MD, MPH, Debbie Travers, PhD, RN, Laura A. Mosqueda, MD, Samuel A.  McLean, MD, MPA, Timothy F. Platts-Mills, MD, MSc | 2020 | Journal for American Geriatric Society | Manuscript | To characterize assessments of a patient' ability to report elder abuse within the context of an emergency department (ED) based screen for elder abuse | Health equity/social EM | Primary enrollment | No, only ED | Cross sectional study | No | English | No | ED patients, aged 65 years or older | on a psychiatric hold, enrolled in another research study, critically ill, receiving face-mask oxygen or positive pressure ventilation, already admitted or discharged, or not in their room during at least two attempts |
| Trajectories of driving after drinking among marijuana-using youth in the emergency department: Substance use, mental health, and peer and parental influences | Aaron D. Dora-Laskey, Jason E. Goldstick, Lisa Buckley, Erin E. Bonar, Marc A. Zimmerman, Maureen A. Walton, Rebecca M. Cunningham, and Patrick M. Carter | 2020 | SUBSTANCE USE & MISUSE | Manuscript | Investigated the relationships of driving after drinking trajectories and negative peer and parental influences, substance use, and mental health among predominantly marijuana-using youth seeking ED treatment | Health equity/social EM | Secondary analysis | No, only ED | Cohort study | No | English | Yes | drug-using youth (ages 14-24) seeking ED care for assault or as part of a non-assaulted comparison group | Patients presenting with sexual assault, suicidality, suspected child abuse, or any cognitive impairment precluding consent (e.g., alcohol intoxication, psychosis) werd dxcluded, as were non-English speakers (<1%) and those in active police custody (3.2%) |
| Associations between emergency department crowding and perceptions of interpersonal care in patients presenting with suspected acute coronary syndrome | Nadia A Liyanage-Don, David S Edelman, Bernard P Chang, Katharina Schultebraucks, Anusorn Thanataveerat, Ian M Kronish | 2022 | Emergency Medicine Journal | Manuscript | Examined whether ED crowding was associated with perceptions of interpersonal care in patients evaluated for acute coronary syndrome | Other clinical study | Secondary analysis | No, only ED | Cohort study | No | English; Spanish | No | age >18 years and suspected ACS | ST-elevation myocardial infarction, terminal non-cardiovascular illness, severe cognitive impairment or inability to complete the study |
| Impacts of an opioid overdose prevention intervention delivered subsequent to acute care | Caleb J Banta-Green, Phillip O Coffin, Joseph O Merril l, Jeanne M Sears, Chris Dunn, Anthony S Floyd, Lauren K Whiteside, Norbert D Yanez, Dennis M Donovan | 2019 | British medical journal | Manuscript | Tested an intervention for opioid users at elevated risk for overdose that was delivered during or after an acute care episode that combined opioid overdose education, a take-home naloxone kit and brief behaviour change counselling to determine the impact on participants‚Äô subsequent opioid overdoses, ED visits and hospitalisations | Health equity/social EM | Primary enrollment | Other: other hospital units during subsequent inpatient admission or at respite care (a recuperative care facility adjacent to HMC for homeless people who require medical assistance and shelter and do not require inpatient treatment) | RCT | Yes | English | No | being at elevated risk of opioid overdose based on: (1) reason for visit was opioid overdose; (2) use of pharmaceutical opioids not prescribed two or more times in the prior month; (3) use of other opioids, alcohol, sedatives or stimulants within 2hours of using opioids two or more times in the prior month; (4) average daily dose of prescribed opioids greater than 10mg morphine equivalent dose or higher for 15 or more of the last 30 days; or (5) enrolled in an opioid agonist therapy (OAT) programme and receiving methadone or buprenorphine; opioids needed to be used at least twice in the last 30 days (or if institutionalised recently, in the most recent month they were  not institutionalised) with pharmaceutical users also needing to have other risks present | 1) refusing access to follow-up medical or drug treatment records; (2) inability to communicate in English; (3) current suicidal ideation; (4) significant cognitive or psychiatric impairment; (5) inability to provide adequate contact information to assist with follow-up (the number of required contacts was reduced after a month of recruitment from three to one as most homeless people were being excluded that would have negatively impacted the generalisability of the findings); (6) under age 18 years or over age 70 years; (7) not living in Washington State or planning to move from Washington State within a year; (8) receiving treatment for sexual assault; or (9) currently having non-expired naloxone |
| Health-Related Material Needs and Substance Use Among Emergency Department Patients | Evan Gerber, BS, Lillian Gelberg, MD, MSPH, John Rotrosen, MD, Donna Castelblanco, MBE, Tod Mijanovich, PhD, and Kelly M. Doran, MD, MHS | 2020 | SUBSTANCE ABUSE | Manuscript | Examined the intersection between ED patient health-related material needs and substance use | Health equity/social EM | Primary enrollment | No, only ED | Cross sectional study | No | English; Spanish | No | 18 years old, medically and psychiatrically stable, not in prison/police custody | RAs were instructed to return to patients who were too intoxicated to provide informed consent after they were more sober |
| Improving Follow-Up Attendance for Discharged Emergency Care Patients Using Automated Phone System to Self-Schedule: A Randomized Controlled Trial | Kyla L. Bauer, Omolade O. Sogade, Brian F. Gage, Brent Ruoff, and Lawrence M. Lewis | 2020 | Society for Academic Emergency Medicine | Manuscript | Determine if a bidirectional, automated phone and text messaging system to help discharged patients schedule their own follow-up appointments and find alternative providers when necessary improved follow-up adherence. | Other clinical study | Primary enrollment | No, only ED | RCT; Clinical trial | Yes | English | No | 1) age 18 years or older, 2) accessible short message service (SMS)-capable mobile phone or residential landline, 3) able to read English or have English-speaking family member to assist with phone communications, 4) discharged directly from the ED, and 5) given a clinical referral to make an outpatient follow-up appointment at time of discharge to a specific clinic or provider | 1) were unable or refused to provide consent, 2) could not be contacted by a phone call or SMS, 3) non-English speaking, 4) were admitted to the hospital, and 5) already had a follow-up appointment scheduled before being discharged from the ED |
| GAPcare: The Geriatric Acute and Post-Acute Fall Prevention Intervention in the Emergency Department: Preliminary Data | Elizabeth M. Goldberg, MD, ScM, Sarah J. Marks, MS, Aderonke Ilegbusi, MPH, Linda Resnik, PT, PhD, Daniel H. Strauss, BA, and Roland C. Merchant, MD, MPH, ScD | 2019 | Journal of the American Geriatrics Society | Manuscript | Fall prevention intervention for older adults | Other clinical study | Primary enrollment | No, only ED | RCT | Yes | English; Spanish | No | 65 years old and older were eligible to participate if they presented to the ED within 7 days of a fall, and their ED clinician determined they were likely to be discharged from the ED | altered mental status (eg, intoxicated), who were undomiciled, or could not provide a phone number |
| Human Papillomavirus Awareness, Vaccine Status, and Risk Factors in Female Emergency Patients | Lauren A. Walter, Elizabeth Leader, James W. Galbraith | 2020 | Western Journal of Emergency Medicine | Manuscript | To gather information pertaining to HPV and cervical cancer risk factors, awareness of HPV and the vaccine, as well as HPV vaccine uptake in female patients presenting to an ED | Other clinical study | Primary enrollment | No, only ED | Cross sectional study | No | English | No | Adult female patients, aged 18-32 | who did not speak English or for whom communication barriers were present (eg, altered mental status, dementia); were medically or psychiatrically unstable; or who were undergoing active emergent evaluation or treatment; and individuals presenting for sexual assault |
| Gender Preferences of Patients When Selecting Orthopaedic Providers | Hannah A. Dineen, MD; J. Megan M. Patterson, MD; Scott M. Eskildsen, MD; Zoe S. Gan, BA;  Quefeng Li, PhD; Brendan C. Patterson, MD; Reid W. Draeger, MD | 2019 | Iowa Orthopedic Journal | Manuscript | To determine whether patients have a preference for the gender of their orthopaedic surgeon, and what traits in orthopaedic surgeons are important to their patients | Other clinical study | Primary enrollment | Other: urgent care for outpatient orthopaedic complaints | Cross sectional study | No | English | No | patients seen consecutively in the Emergency Department and orthopaedic urgent care | under the age of 18 years old, or an established orthopaedic patient to minimize bias from an ongoing relationship with an orthopaedic provider |
| Prevalence and Temporal Characteristics of Housing Needs in an Urban Emergency Department | Leah Fraimow-Wong, BA Jennifer Sun, BA Partow Imani, MS Daniel Haro, BA Harrison J. Alter, MD, MS | 2021 | Western Journal of Emergency Medicine | Manuscript | To determine the proportion of patients in our ED who are unhoused or marginally housed and when they typically present to the ED | Health equity/social EM | Primary enrollment | No, only ED | Cross sectional study | No | English; Spanish | No | 18 years of age or older | medically unstable, unresponsive, or had altered mental status were not surveyed, nor were those who had already participated |
| Gender Differences in Pain Experience and Treatment after Motor Vehicle Collisions: A Secondary Analysis of the CRASH Study | Tracy E. Madsen, Samuel McLean, Wanting Zhai, Sarah Linnstaedt, Michael C. Kurz, Robert Swor, Phyllis Hendry, David Peak, Christopher Lewandowski, Claire Pearson,  Brian O'Neil, Elizabeth Datner, David Lee, and Francesca Beaudoin | 2018 | Clinical Therapeutics | Manuscript | To describe gender differences in pain experiences and treatment, specifically the use of opioids and benzodiazepines after ED discharge, for MVC-related pain | Health equity/social EM | Secondary analysis | No, only ED | Cohort study | No | English | No | patients 18 to 65 years old presenting to one of 8 participating EDs across 4 states (Florida, Massachusetts, Michigan, and New York) within 24 hours of a MVC from February 2009 to October 2011 were eligible for enrollment. For the AA cohort, patients 18 to 65 years old presenting to one of 13 participating EDs across 7 states (Alabama, Florida, Michigan, Pennsylvania, Washington, D.C., Massachusetts, New Jersey) within 24 hours of a MVC from 2012 to 2016 were eligible for enrollment. | admitted to the hospital, had fractures with the exception of phalanx fractures, had four or more lacerations requiring sutures or one laceration greater than 20 centimeters long, or had intracranial or spinal injuries, not alert and oriented, were pregnant, were inmates |
| Predictors of Older Adult Adherence with Emergency Department Discharge Instructions | Ivy Benjenk, BSN, MPH, Eva H. DuGoff, PhD, MPP, Gwen C. Jacobsohn, PhD, Nia Cayenne, BS, Courtney M. C. Jones, PhD, MPH, Thomas V. Caprio, MD, MPH, MS, Jeremy T. Cushman, MD, MS, Rebecca K. Green, MPH, Amy J. H. Kind, MD, PhD, Michael Lohmeier, MD, Ranran Mi, MA, and Manish N. Shah, MD, MPH | 2020 | Society for Academic Emergency Medicine | Manuscript | Determine the individual-level factors associated with adherence with ED discharge instructions among older adult ED outpatients | Health equity/social EM | Secondary analysis | No, only ED | RCT | Yes | English | No | age >60 years, having a primary care physician within the ED‚Äôs health care system, community-dwelling, and having a working phone | admitted to the hospital, stayed in the ED longer than 24 hours, were discharged from the ED to hospice or a long-term care facility, were homeless, had a transitional care team or intensive care management team in place, or were in the ED primarily for a behavioral health indication |
| Impact of Emergency Physician-Provided Patient Education About Alternative Care Venues | Pankaj B. Patel, MD, David R. Vinson, MD, Marla N. Gardner, BA, David A. Wulf, BS, Patricia Kipnis, PhD, Vincent Liu, MD, MS, and Gabriel J. Escobar, MD | 2018 | American Journal of Managed Care | Manuscript | To evaluate the effects of mailed information and a brief scripted educational phone call from an emergency physician on subsequent ED utilization by low-risk adults with a recent treat-and-release ED visit | Other clinical study | Primary enrollment | No, only ED | RCT | Yes | English | No | 18 years or older, Kaiser membership, and a low-acuity treat-and-release ED visit without PCP contact in the 24 hours prior to their ED visit | could not respond on their own or through a family member or guardian, and those who died during the 6-month postintervention period |
| Randomized Trial of Intravenous Lidocaine versus Hydromorphone for Acute Abdominal Pain in the Emergency Department | Elliott Chinn, DO, Benjamin W. Friedman, MD, MS, Farnia Naeem, BS, Eddie Irizarry, MD, Freda Afrifa, PharmD, Eleftheria Zias, RPh, Michael P. Jones, MD, Scott Pearlman, MD, Andrew Chertoff, MD, Andrew Wollowitz, MD, E. John Gallagher, MD | 2019 | Annals of Emergency Medicine | Manuscript | Compared the efficacy and safety of intravenous lidocaine to that of hydromorphone for the treatment of acute abdominal pain in the ED | Other clinical study | Primary enrollment | No, only ED | RCT | Yes | English; Spanish | No | 8 and 64 years old, weighed between 60 and 120 kg, and presented to one of our EDs for treatment of acute, severe abdominal pain | cardiac conduction system impairment, known renal or liver disease, hemodynamic instability (as determined by the attending physician), pregnancy, breastfeeding, or for allergy to either medication, self-reported use of prescription or illicit opioids within the previous week, or if they had a chronic pain disorder |
| Development of the Emergency Department Senior Abuse Identification (ED Senior AID) tool | Timothy F. Platts-Mills, MD, MS, Joseph A. Dayaa, MS, Bryce B. Reeve, PhDb, Kayla Krajick, BSc, Laura Mosqueda, MD, Jason S. Haukoos, MD, MS, Mehul D. Patel, PhD, MSPH, Carrie F. Mulford, PhD, Samuel A. McLean, MD, MPH, Phil D. Sloane, MD, MPH, Debbie Travers, PhD, RN, Sheryl Zimmerman, PhD, MSW | 2018 | Journal for Elder Abuse Neglect | Manuscript | To develop an ED-based tool to identify elder abuse | Health equity/social EM | Primary enrollment | No, only ED | Cross sectional study; Qualitative | No | English | No | 65 years and older presenting to the ED | ESI 1, on a psychiatric hold, were receiving facemask oxygen or positive pressure ventilation, were enrolled in another research study, or if access to the patient was restricted by a medical provider |
| Risk of Posttraumatic Stress Disorder and Major Depression in Civilian Patients After Mild Traumatic Brain Injury: A TRACK-TBI Study | Murray B. Stein, Sonia Jain Joseph T Giacino, Harvey Levin, Sureyya Dikmen, Lindsay D. Nelson, Mary J Vassar, David O Okonkwo, Ramon Diaz-Arrastia, Claudia S Robertson, Pratik Mukherjee, Michael McCrea, Christine L Mac Donald, John K Yue, Esther Yuh, Xiaoying Sun, Laura Campbell-Sills, Nancy Temkin, Geoffrey T Manley, and the TRACK-TBI Investigators | 2019 | JAMA Psychiatry | Manuscript | Who is at greatest risk for developing mental health problems such as posttraumatic stress disorder (PTSD) or major depression after sustaining a mild traumatic brain injury (mTBI) | Other clinical study | Primary enrollment | No, only ED | Cohort study | No | English; Spanish | No | GCS scores of 13 to 15 on arrival in the ED and received a CT scan per order of the evaluating ED physician, injury occurrence within 24 hours of admission, acute brain CT imaging ordered for clinical care purposes | significant multiple trauma that would interfere with follow-up; penetrating TBI; prisoners or patients in custody; pregnancy; patients whose reason for being in the ED was for mandated psychiatric assessment; major debilitating mental (eg, schizophrenia, bipolar disorder) or neurological disorders (eg, stroke, dementia) or any other disorder that would interfere with assessment and follow-up or provision of informed consent; and current participation in a clinical therapeutic trial |
| Acute Clinical Predictors of Symptom Recovery in Emergency Department Patients with Uncomplicated Mild Traumatic Brain Injury or Non-Traumatic Brain Injuries | Lindsay D. Nelson, Robyn E. Furger, Jana Ranson, Sergey Tarima, Thomas A. Hammeke, Christopher Randolph, William B. Barr, Kevin Guskiewicz, Christopher M. Olsen, E. Brooke Lerner, and Michael A. McCrea | 2018 | JOURNAL OF NEUROTRAUMA | Manuscript | To investigate the predictors of symptom recovery in a prospective sample of emergency department trauma patients with either mild TBI or non-mild TBI injuries | Other clinical study | Secondary analysis | No, only ED | Cohort study | No | English | No | 18-45 (the age range of interest to the study sponsor), initial Glasgow Coma Scale score 13‚Äì15, loss of consciousness (LOC) <30 min, post-traumatic amnesia <24 h, absence of acute intracranial findings on brain imaging | injury that precluded participation in the study protocol (e.g., hand injury that prevented use of a computer mouse), current diagnosis of a psychotic disorder, history or clinical suspicion of other conditions (e.g., epilepsy, stroke, dementia) known to cause cognitive dysfunction, or a history of moderate or severe TBI |
| Latent Class Analysis of Barriers to Care Among Emergency Department Patients | Abar, Beau Holub, Ashley Hong, Steven et al | 2019 | Western Journal of Emergency Medicine: Integrating Emergency Care with Population Health | Manuscript | Social needs/health equity (Participants reported on the extent to which their ability  to see a doctor in the prior year was limited by nine specific  barriers) | Health equity/social EM | Primary enrollment | No, only ED | Cross sectional study | No | English | No | patients >18 years of age | presentation to the psychiatric ED, presentations for intoxication, suicide attempt, mental health arrest or overdose, and patients who had an Emergency Severity Index score of 1 and/or were in the critical care bay |
| Factors associated with risky drinking, cannabis & illicit drug use and intimate partner violence among hispanics: An emergency department study | C.B. Cunradi; R. Caetano; H.J. Alter; W.R. Ponicki | 2021 | Research Society on Alcohol | Abstract | Analyzed the interrelationships of socioeconomic stressors, mental health problems, substance use, and intimate partner violence (IPV) among a sample of socially disadvantaged Hispanic men and women | Health equity/social EM | Primary enrollment | No, only ED | Cross sectional study | No | English; Spanish; Other: used bilingual RAs | No | married, cohabiting, or in a romantic relationship for the past 12 months | unable to provide consent |
| Missed hiv & hcv screening in emergency-department patients with opioid-use disorder | Michael S. Lyons, MD, MPH, Marek C. Chawarski, PhD, Richard Rothman, MD, PhD,  Lauren Whiteside, MD, Ethan Cowan, MD, MS, Lynne D. Richardson, MD, Kathryn Hawk, MD, MHS, Judith I. Tsui, MD, MPH, Robert P. Schwartz, MD, Patrick O‚ÄôConnor, MD, MPH, Gail D'Onofrio, MD, MS, David A. Fiellin, MD, E. Jennifer Edelman, MD, MHS | 2023 | Addiction Medicine | Manuscript | Assessed the frequency of emergency department HIV and HCV screening in a high-risk cohort of ED patients with untreated opioid use disorder | Health equity/social EM | Secondary analysis | No, only ED | Cohort study; Other: prospective, observational study | No | English | No | adult >18 years old, in the ED during enrollment hours, had moderate to severe untreated OUD, urine toxicology indicated opioids other than or in addition to fentanyl before any ED opioid medication for clinical care | unable to provide consent |
| Empower Seriously Ill Older Adults to Formulate Their Goals for Medical Care in the Emergency Department | Kei Ouchi, MD, MPH, Naomi George, MD, Anna C. Revette, PhD, Mohammad Adrian Hasdianda, MD, Lauren Fellion, PA-C, Audrey Reust, PA-C, Lynda H. Powell, PhD, Med, Rebecca Sudore, MD, Jeremiah D. Schuur, MD, MHS, Mara A. Schonberg, MD, MPH, Edward Bernstein, MD, James A. Tulsky, MD, and Susan D. Block, MD | 2019 | JOURNAL OF PALLIATIVE MEDICINE | Manuscript | To develop an intervention to empower seriously ill older adults to formulate their future care goals in the ED | Other clinical study | Primary enrollment | No, only ED | Non randomized experimental study ; Cohort study; Other: prospective intervention development study | Yes | English | No | >65 years old with serious illness or patients whose treating ED clinician answered "No" to the "surprise question"("would not be surprised if died in the next 12 months") | advance directives or whose treating ED clinician determined the patient to be inappropriate |
| Medication Allergy and Adverse Drug Reaction Documentation Discrepancies in an Urban, Academic Emergency Department | Eric S. Kiechle & Colleen M. McKenna & Hannah Carter & Alexander Zeymo & Bradley W. Gelfand & Lindsey M. DeGeorge & Diane A. Sauter & Maryann Mazer-Amirshahi | 2018 | Journal of Medical Toxicology | Manuscript | To determine the prevalence and nature of medication allergy and adverse drug reactions discrepancies | Other clinical study | Primary enrollment | No, only ED | Cross sectional study | No | English | No | >18 years old, conscious and clinically stable, and able to provide informed consent | undergoing emergent stabilization or were unwilling or unable to provide consent |
| Co-Occurrence of Multiple Risk Factors and Intimate Partner Violence in an Urban Emergency Department | Raul Caetano, MD, PhD Carol B. Cunradi, MPH, PhD Harrison J. Alter, MD, MPH Christina Mair, PhD | 2020 | Western Journal of Emergency Medicine: Integrating Emergency Care with Population Health | Manuscript | We examine the 12-month rate of physical IPV and its association with multiple joint risk factors in an urban ED. | Health equity/social EM | Primary enrollment | No, only ED | Cross sectional study | No | English; Spanish | No | 18-50 years old; residence in the county where the study was conducted; and married, cohabiting, or in a romantic (dating) relationship for the prior 12 months | intoxicated, experiencing acute psychosis or suicidal or homicidal ideation, were cognitively and/or psychologically impaired and unable to provide informed consent, in custody by law enforcement, or in need of immediate medical attention |
| Language equivalence of the modified falls efficacy scale (MFES) among English- and Spanish-speaking older adults: Rasch analysis | Robert J. Lucero, Sergio Romero, Robert Fieo, Yamnia Cortes, Jeannie P. Cimiotti and Lusine Poghosyan | 2020 | BMC Geriatrics | Manuscript | To investigate item-level measurement properties of the Modified Falls Efficacy (MFES) Scale among English- and Spanish-speaking urban-dwelling older adults as a means to evaluate language equivalence of the tool | Health equity/social EM | Secondary analysis | No, only ED | Cross sectional study | No | English; Spanish | No | at least 55 years of age, admitted to the ED, and discharged home with a nonserious injury | cognitive impairment |
| Emergency Department Patients' COVID-19 Vaccination Status and Self-Reported Barriers | Bethany W. Harvey, MD Kyle J. Kelleran, PhD Heidi Suffoletto, MD Changxing Ma, PhD Nan Nan, MA Michelle D. Penque, MD E. Brooke Lerner, PhD | 2022 | Western Journal of Emergency Medicine | Manuscript | To identify self-reported barriers to COVID 19 vaccination and possible areas for intervention | Other clinical study | Primary enrollment | No, only ED | Cross sectional study | No | English | No | each adult patient in the ED was considered for enrollment | too ill to participate, not capable of providing consent, actively receiving care, being subject to infectious precautions, or sleeping |
| Patient-Reported Opioid Pill Consumption After an ED Visit: How Many Pills Are People Using? | Danielle M. McCarthy , MD, MS, Howard S. Kim, MD, MS, Scott I. Hur, MPH, Patrick M. Lank, MD,MS, Christine Arroyo, Lauren A. Opsasnick, MS, Katherine Piserchia, Laura M. Curtis, MS, Michael S. Wolf, PhD MPH, and D. Mark Courtney, MD, MS | 2021 | Pain Medicine | Manuscript | To describe opioid consumption patterns after an ED visit for acute pain | Other clinical study | Secondary analysis | No, only ED | RCT | Yes | English | Yes | age >17 years, received a prescription for hydrocodone-acetaminophen | chronically use opioids |
| Use of a tablet computer application to engage patients in updating their medication list | Kimberly Hart, M.A., Sunil Kripalani, M.Sc., Caitlin Schaninger, M.D., Stuart Bracken, M.B.A., Christopher Lindsell, Ph.D., Dane R. Boyington, Ph.D. | 2019 | American Journal of Health-System Pharmacy | Manuscript | The development and initial evaluation of the PictureRx Medication History Application, a tablet computer-based program that queries patients‚Äô prescription fill data from the Surescripts Medication History service and renders it graphically for review and editing at the point of care | Other clinical study | Primary enrollment | No, only ED | Non randomized experimental study ; Other: quasi-experimental trial | Yes | English | No | located in one of the intervention rooms with a tablet device, at least 18 years of age, and reported taking at least one prescription medication | too ill or otherwise unable to complete informed consent |
| Predictors and Reasons Why Patients Decline to Participate in Home Hospital: a Mixed Methods Analysis of a Randomized Controlled Trial | David M Levine, MD MPH MA, Mary Paz, BS, Kimberly Burke, BS, and Jeffrey L Schnipper, MD MPH | 2021 | Journal of General Internal Medicine | Manuscript | Describe predictors and reasons why patients decline home hospital | Other clinical study | Secondary analysis | No, only ED | RCT | Yes | English; Spanish; Portuguese ; French; Creole; Chinese (including Mandarin); Arabic; Vietnamese ; Korean; Russian; Other: did not specify in methods, but English Spanish and Other are in table in results | No | eligible for inclusion based on their home's geographic location, their illness type, and their acuity | Patients were ineligible if they required critical care, an invasive procedure, or advanced imaging, among other criteria |
| Substance use and homelessness among emergency department patients* | Kelly M. Doran, Neloufar Rahai, Ryan P. McCormack, Jacqueline Milian, Donna Shelley, John Rotrosen, Lillian Gelberg | 2018 | Drug and Alcohol Dependence | Manuscript | Examined the overlap of homelessness and substance use in ED patients | Health equity/social EM | Primary enrollment | No, only ED | Cross sectional study | No | English; Spanish | No | >18 years old | medically unstable, in psychological distress, in police/prison custody, could not provide consent, or had already participated |
| Nobody Wants to Be Narcan‚Äôd: A Pilot Qualitative Analysis of Drug Users' Perspectives on Naloxone | Jeffrey T. Lai, MD Charlotte E. Goldfine, MD Brittany P. Chapman, BSc Melissa M. Taylor, BA Rochelle K. Rosen, PhD Stephanie P. Carreiro, MD Kavita M. Babu, MD | 2021 | Western Journal of Emergency Medicine | Manuscript | Evaluate whether individuals' drug use patterns have changed due to naloxone availability and explore individuals' knowledge of, access to, experiences with, and perceptions of naloxone | Health equity/social EM | Primary enrollment | No, only ED | Qualitative | No | English | No | 18-65 years of age, had presented to the ED with an opioid-related chief complaint, had a history of non-medical opioid use, and were able to provide informed consent | previously participated in this study or were in police custody |
| Tricuspid Annular Plane of Systolic Excursion (TAPSE) for the Evaluation of Patients with Severe Sepsis and Septic Shock | Shadi Lahham MD, MS Clifton Lee, BS Qumber Ali, BS John Moeller, MD Chanel Fischetti, MD Maxwell Thompson, MD Soheil Saadat MD, MPH, PhD John C. Fox, MD | 2020 | Western Journal of Emergency Medicine | Manuscript | Evaluation of the tricuspid annular plane of systolic excursion (TAPSE) in patients with sepsis | Other clinical study | Primary enrollment | No, only ED | Cohort study | No | English; Spanish | No | at least 18 years old and were undergoing evaluation for sepsis and septic shock | pregnant, incarcerated, mechanically ventilated prior to initial evaluation, unable to provide medical consent, history of pulmonary hypertension, known pulmonary  embolism, or heart failure |
| Mechanisms of Behavior Change in a Brief Dual-target Motivational Intervention: Reduction in Alcohol Use Mediates Intervention Effects on Risky Sex | Nancy P. Barnett, Suzanne M. Colby, Christopher W. Kahler, Don Operario, and Peter M. Monti | 2019 | Psychology of Addictive Behaviors | Manuscript | The efficacy of brief dual-target motivation intervention to reduce alcohol use and sexual risk behavior | Health equity/social EM | Secondary analysis | No, only ED | Cross sectional study | Yes | English | No | English speaking ages 18 to 65, meeting the criterion for hazardous drinking (total score 8 for males and 6 for females) on the Alcohol Use Disorders Identification Test or endorsing at least one episode of binge drinking (5 drinks for males, 4 drinks for females) in the last 3 months and engaging in one or more of the following sex-risk behaviors in the last 3 months: condomless sex (i.e., vaginal or anal); consuming alcohol/other drugs prior to or during sex; or sexual activity with a nonsteady partner, multiple partners, or with a steady partner where infidelity is known or suspected | mutually monogamous relationship for longer than 6 months, those receiving treatment for a self-inflicted injury/suicide attempt, and those in police custody |
| The quality of symptoms in women and men presenting to the emergency department with suspected acute coronary syndrome | Sahereh Mirzaei, MSN, RN, Alana Steffen, PhD, Karen Vuckovic, PhD, APRN, FAHA, Catherine Ryan, PhD, RN, FAHA, FAAN, Ulf Bronas, PhD, FAHA, FSVM, Jessica Zegre-Hemsey, PhD, RN, Holli A. DeVon, PhD, RN, FAHA, FAAN | 2020 | Journal of Emergency Nursing | Manuscript | To describe the quality of symptoms (chest discomfort/pain description, location/radiation, and overall symptom distress) reported by women  and men ruled-in and ruled-out for ACS in the EDs | Other clinical study | Secondary analysis | No, only ED | Cohort study | No | English | No | 21 years old, and arrived by private transportation or emergency medical service | heart failure (brain natriuretic peptide > 500 pg/ml), referred for dysrhythmia evaluation, transferred from a dialysis center, or cognitive impairment that precluded them from providing informed consent |
| MyEDCare: Evaluation of a Smartphone-Based Emergency Department Discharge Process | Peter A. D. Steel, David Bodnar, Maryellen Bonito, Jane Torres-Lavoro, Dona Bou Eid, Andrew Jacobowitz, Amos Shemesh, Robert Tanouye, Patrick Rumble, Daniel DiCello, Rahul Sharma, Brenna Farmer, Sandra Pomerantz, Yiye Zhang | 2021 | Applied Clinical Informatics Journal | Manuscript | Evaluated the feasibility of utilizing MyEDCare, a text message and smartphone-based electronic ED discharge process at two urban EDs | Other clinical study | Primary enrollment | No, only ED | Cohort study | Yes | English | No | discharged from the ED who did not opt-out from MyEDCare were enrolled in the process | discharged to locations that would require paper documentation (e.g., to law enforcement custody, skilled care facilities, shelters), patients whose employers requested discharge paperwork, patients discharged with a primary psychiatric diagnosis |
| Individual and Social Factors Related to Trajectories of Blackouts among Underage Drinkers in the Emergency Department | Erin E. Bonar, Jason E. Goldstick, Rebecca M. Cunningham, Anne C. Fernandez, Alan K. Davis, Mark A. Ilgen, and Maureen A. Walton | 2019 | Alcohol and Alcoholism | Manuscript | Examined longitudinal trajectories of blackout frequency following an ED visit and identified baseline characteristics associated with blackout trajectory membership | Other clinical study | Primary enrollment | No, only ED | RCT | Yes | English | Yes | ED patients (ages 14-20) | presenting with suicidal ideation, sexual assault or child abuse, altered mental status, and absence of a parent/guardian to provide consent (if under age 18) |
| Randomized Clinical Trial of IV Acetaminophen as an Analgesic Adjunct for Older Adults with Acute Severe Pain | Andrew K. Chang, MD, MS, Polly E. Bijur, PhD, Ashar Ata, PhD, Caron Campbell, MD, Scott Pearlman, MD, Deborah White, MD, Andrew Chertoff, MD, Andrew Restivo, MD, and E. John Gallagher, MD | 2018 | Society for Academic Emergency Medicine | Manuscript | Examined intravenous acetaminophen as an analgesic adjunct to IV opioids in the care of older ED patients with acute severe pain | Other clinical study | Primary enrollment | No, only ED | RCT | Yes | English; Spanish | No | aged 65 years and older presenting to the ED with acute pain (onset within 7 days) were enrolled if their pain was of sufficient severity to warrant use of IV opioids | history of adverse reaction to hydromorphone, morphine, or acetaminophen; hypotension; room air oxygen saturation < 95%; CO2 measurement > 46; heart rate < 60 beats/min; alcohol or other drug intoxication as judged by the attending physician; use of other opioids within the past 24 hours; use of a monoamine oxidase inhibitor within 30 days; history of a chronic pain syndrome; and patients with transdermal pain patches |
| Partner Presence in the Emergency Department and Adherence to Daily Cardiovascular Medications in Patients Evaluated for Acute Coronary Syndrome | Talea Cornelius, Jeffrey L. Birk, Kyle Bourassa,  Redeana C. Umland, Ian M. Kronish | 2020 | Journal of Behavioral Medicine | Manuscript | Tested whether partner status/partner presence in the ED were associated with patients' adherence to daily cardiovascular medications and whether  weffects differed by age/gender | Other clinical study | Primary enrollment | No, only ED | Cohort study; Other: ancillary study off of a parent study | No | English; Spanish | No | at least 18 years of age who presented to the ED with an admitting diagnosis of NSTEMI or UA | (1) terminal non-cardiovascular illness with life expectancy<1 year; (2) severe mental illness; (3) significant cognitive impairment; (4) known active  alcohol or substance use disorder; and (5) unavailability for follow-up. For the ancillary medication adherence study, additional exclusion criteria were: (6) not prescribed a cardiovascular medication at discharge; (7) unable to self-administer medications; and (8) concern that using the eCAP would disrupt their usual medication-taking behavior (e.g., patients who use only pillboxes) |
| "I wanted to participate in my own care": Evaluation of a Patient Navigation Program | Elizabeth A. Samuels, MD, MPH, MHS Lauren Kelley, MSW, MPA Timothy Pham, MPH Jeremiah Cross, MD Juan Carmona, MS Peter Ellis, MD, MPH Darcey Cobbs-Lomax, MBA, MPH Gail D‚ÄôOnofrio, MD, MS Roberta Capp, MD, MHS | 2021 | Western Journal of Emergency Medicine | Manuscript | To understand patient experiences and satisfaction with an ED-initiated patient navigation intervention for US Medicaid-enrolled frequent ED users | Health equity/social EM | Primary enrollment | No, only ED | Cohort study | Yes | English; Spanish | No | 18-62 years old; Medicaid-enrolled; residents of one of the twelve towns in the greater New Haven area; had 4-18 visits to ED in the prior year; less than 50% of their prior year ED visits were for a psychiatric or substance use concern; and they were not being primarily treated for a psychiatric or substance use concern at the time of enrollment | frequent ED utilization for substance use disorders and behavioral health problems because they have additional and often complex clinical, behavioral, and social needs that the intervention was not designed or equipped to support |
| Interventions to Improve Adherence to Cervical Cancer Screening Recommendations among Emergency Department Patients: Enrollment Data in Anticipation of Interventional Trial Results | David Adler, MD, MPH Beau Abar, PhD Nancy Wood, MS Adrienne Bonham, MD | 2019 | The Journal of Emergency Medicine | Abstract | Improve adherence to cervical cancer screeing recommendations among emergency department patients. | Other clinical study | Primary enrollment | No, only ED | RCT | Yes | English | No | female sex and age 21-65 years | past hysterectomy with cervical removal, known human immunodeficiency virus infection, inability to consent, non‚ÄìEnglish-speaking, or lack of text-capable mobile phone and/or inability to use text function |
| Effect of Intranasal Vasoconstrictors on Blood Pressure: A Randomized, Double-Blind, Placebo-Controlled Trial | Shawna D. Bellew, MD, Katie L. Johnson, MD, Micah D. Nichols, and Tobias Kummer | 2018 | The Journal of Emergency Medicine | Manuscript | Determine the effects of intranasal vasoconstrictors on blood pressure | Other clinical study | Primary enrollment | No, only ED | RCT; Clinical trial | Yes | English | No | patients discharged from the ED | younger than 18 years, had known allergies to any of the study agents, currently receiving antihypertensive or antidysrhythmic agents, had clinically significant cardiopulmonary comorbidities (e.g., a history of hypertension, dysrhythmia, coronary artery disease, heart failure), were known to be using a monoamine oxidase inhibitor agent, or had a history of angle-closure glaucoma, benign prostatic hyperplasia, nasal surgery, or nasal abnormalities |
| Patient-reported symptoms improve prediction of acute coronary syndrome in the emergency department | Jessica K. Zgre-Hemsey, Larisa A. Burke, Holli A. DeVon | 2018 | Research in Nursing & Health | Manuscript | (i)the prognostic value of symptoms for an ACS diagnosis in conjunction with electrocardiographic (ECG) and troponin results; and (ii) if any of 13 symptoms were associated with prehospital delay in those presenting to the emergency department (ED) with potential ACS | Other clinical study | Secondary analysis | No, only ED | Cohort study | No | English | No | Patients >21 years of age and with symptoms that triggered an evaluation for ACS | did not speak english |
| Homeless Shelter Entry in the Year Following an Emergency Department Visit: Results from a Linked Data Analysis | Kelly M. Doran, MD, MHS; Eileen Johns, MPA; Maryanne Schretzman, DSW; Sara Zuiderveen, MPP; Marybeth Shinn, PhD; Rajneesh Gulati, MD; Ian Wittman, MD; Dennis Culhane, PhD; Donna Shelley, MD, MPH; Tod Mijanovich, PhD | 2020 | Annals of Emergency Medicine | Manuscript | Determine the incidence and timing of homeless shelter entry after an ED visit among patients who are not currently homeless | Health equity/social EM | Primary enrollment | No, only ED | Cohort study | No | English; Spanish | No | aged 18 years or older | medically unstable or in physical distress, were too intoxicated to participate, were in psychological distress, were in police or prison custody, could not provide consent (eg, dementia), lived outside NYC, or had already participated |
| Substance Use Reporting as a Predictor of HIV Test Rates Among Emergency Department Patients | I. D. Aronson, C. M. Cleland, S. Rajan, L. A. Marsch, T. C. Bania | 2019 | AIDS and Behavior | Manuscript | Whether or not watching a video of same race person explaining the benefits of HIV testing increased rates of testing in patients | Health equity/social EM | Primary enrollment | No, only ED | Cross sectional study | Yes | English | No | declined an HIV test offered at triage; were aged 18 years or older; and were not known to be HIV positive | a prisoner; classified by ED staff as in most urgent need of medical care or experiencing a severe psychological problem; intoxicated; unconscious |
| Patient Characteristics and Perspectives of Firearm Safety Discussions in the Emergency Department | Lauren Hudak, MD, MPH Henry Schwimmer, MD William Warnock, BA Sarah Kilborn, MD Tim Moran, PhD Jeremy Ackerman, MD, PhD Jonathan Rupp, PhD | 2021 | Western Journal of Emergency Medicine | Manuscript | Examining ED patient baseline characteristics, perspectives on healthcare-based safety discussions, and experience with and access to firearms, will allow practitioners to craft more effective messaging and interventions | Health equity/social EM | Primary enrollment | No, only ED | Cross sectional study | No | English | No | 18 years of age, cognitively impaired, medically unstable, in police custody, had previously participated) | unable to provide consent |
| The Impact of Support Provided by Close Others in the Emergency Department on Threat Perceptions | Talea Cornelius, Lilly Derby, Melissa Dong & Donald Edmondson | 2020 | Psychology & Health | Manuscript | Tested the hypothesis that close others provide more negative support than non-close others as a potential explanation for this effect | Other clinical study | Secondary analysis | No, only ED | Cohort study | No | English; Spanish | No | 1) age 18 years, (2) fluent in English or Spanish, and (3) an admitting diagnosis of NSTEMI or UA when presenting to the ED | terminal non-cardiovascular illness with life expectancy < 1 year, (2) severe mental illness requiring urgent psychiatric hospitalisation or intervention, (3) significant cognitive impairment, (4) alcohol or substance abuse that would impede ability to complete study protocol, and (5) unavailability for follow-up  To be included in this secondary analysis, patients had to have arrived in the ED with a companion and provided information on support provided by that companion |
| The Rapid Evaluation of COVID-19 Vaccination in Emergency Departments for Underserved Patients Study | Robert M. Rodriguez, MD; Jesus R. Torres, MD, MPH; Anna Marie Chang, MD, MSCE; Adrianne N. Haggins, MD, MS; Stephanie A. Eucker, MD, PhD; Kelli N. O'Laughlin, MD, MPH; Erik Anderson, MD; Daniel G. Miller, MD; R. Gentry Wilkerson, MD; Martina Caldwell, MD, MS; Stephen C. Lim, MD; Ali S. Raja, MD, MPH; Brigitte M. Baumann, MD, MSCE; Joseph Graterol, MD; Vidya Eswaran, MD; Brian Chinnock, MD | 2021 | Annals of Emergency Medicine | Manuscript | Compared patients having and lacking a regular source of medical care and other ED patient characteristics, we assessed COVID-19 vaccine hesitancy, reasons for not wanting the vaccine, perceived access to vaccine sites, and willingness to get the vaccine as part of ED care | Health equity/social EM | Primary enrollment | No, only ED | Cross sectional study | No | English; Spanish; Portuguese ; French; Creole; Chinese (including Mandarin); Arabic; Vietnamese ; Korean; Russian; Other: translated survey materials | No | patients (>18 years of age) using convenience sampling according to the availability of study personnel | major trauma, transfer from another facility, incarceration, psychiatric hold, intoxication, altered mental status, critical illness, or temporary visit from another country |
| Targeted Screening for HIV Pre-Exposure Prophylaxis Eligibility in Two Emergency Departments in Washington, DC | Paige Kulie, MPH, Amanda D. Castel, MD, MPH, Zhaonian Zheng, MS, Natasha N. Powel MD, MPH, Aneil Srivastava, BS, Sandhya Chandar, BS, and Melissa L. McCarthy, ScD | 2020 | AIDS PATIENT CARE and STD | Manuscript | Estimated the proportion of patients who were PrEP eligible among a targeted sample of emergency department patients with chief complaints indicative of HIV risk | Other clinical study | Primary enrollment | No, only ED | Cross sectional study | No | English | No | 18 years or older, had been sexually active (oral, anal, and/or vaginal) in the past 6 months, were HIV negative, and presented with a chief complaint related to a GU problem, substance use, or intentional injury | previously enrolled, were a prisoner or in police custody, or were unable to provide informed consent |
| Feasibility of emergency department initiated, mobile health, blood pressure intervention: an exploratory, randomized clinical trial | William J. Meurer, MD, MS, Mackenzie Dome, MS, Devin Brown, MD, MS, Destinee Delemos, MD, Sandra Oska, Victoria Gorom, and Lesli Skolarus, MD, MS | 2019 | Academic Emergency Medicine | Manuscript | To assess the feasibility of a text messaging intervention by determining the proportion of ED patients who responded to prompted home blood pressure self-monitoring and had persistent hypertension | Other clinical study | Primary enrollment | No, only ED | RCT | Yes | English | No | Adult ED patients were eligible if they had a documented systolic BP (sBP) of >160 mm Hg or a diastolic BP of >100 mm Hg, were likely to be discharged from the ED, and possessed a mobile phone with text messaging available | critically ill, otherwise unable to give informed consent, incarcerated/institutionalized residents, pregnant, or had a preexisting condition that made follow-up for 4 months unlikely |
| Do High Sensitivity Troponin and Natriuretic Peptide Predict Death or Serious Cardiac Outcomes After Syncope? | Carol L. Clark, MD, MBA, Thomas A. Gibson, MS, Robert E. Weiss, PhD, Annick N. Yagapen, MPH, Susan E. Malveau, MSBE, David H. Adler, MD, MPH, Aveh Bastani, MD, Christopher W. Baugh, MD, MBA, Jeffrey M. Caterino, MD, MPH, Deborah B. Diercks, MD, MPH, Judd E. Hollander, MD, Bret A. Nicks, MD, MHA, Daniel K. Nishijima, MD, MAS, Manish N. Shah, MD, MPH, Kirk A. Stiffler, MD, Alan B. Storrow, MD, Scott T. Wilber, MD, and Benjamin C. Sun, MD, MPP | 2019 | Society for Academic Emergency Medicine | Manuscript | To determine if hscTnT and NT-proBNP drawn in the ED are independently associated with 30-day death/serious cardiac outcomes in adult patients presenting with syncope | Other clinical study | Secondary analysis | No, only ED | Cohort study; Other: observational | No | English; Spanish | No | age >60 years presenting with a chief complaint of syncope or near syncope | presented with seizure, stroke, transient ischemic attack, head trauma, intoxication from drugs or alcohol, or hypoglycemia as the presumptive cause of symptoms, persistent confusion relative to baseline mental status and those who required medical or electrical interventions (e.g., intravenous glucose, defibrillation) to restore consciousness, prior enrollment, patients unlikely to complete follow-up, including those who lacked phone access, lacked a permanent address |
| A qualitative study of emergency department patients who survived an opioid overdose: Perspectives on treatment and unmet needs | Kathryn Hawk MD, MHS, Lauretta E. Grau PhD, David A. Fiellin MD, Marek Chawarski PhD, Patrick G. O'Connor MD, MPH, Nikolas Cirillo MS, Chris Breen, Gail D'Onofrio MD, MS | 2020 | Society for Academic Emergency Medicine | Manuscript | To explore patients' perspectives on substance use treatment, perceived needs, and contextual factors that shape the choice of patients seen in the ED to engage with treatment and other patient support services in the acute post‚Äìopioid overdose period | Health equity/social EM | Primary enrollment | No, only ED | Cross sectional study; Qualitative | No | English | No | had experienced an opioid overdose immediately prior to ED arrival, were not suicidal, were not anticipated to require hospital admission, were not currently receiving MOUD | unable to provide consent |
| Patients' and clinicians' perceptions of antibiotic prescribing for upper respiratory infections in the acute care setting | David A. Broniatowski, Eili Y. Klein, Larissa May, Elena M. Martinez, Chelsea Ware, and Valerie F. Reyna | 2018 | Medical Decision Making | Manuscript | Determine whether patients' rationales for antibiotic use are shared by providers, with the aim of understanding the drivers of effective interventions | Other clinical study | Primary enrollment | Other: online sample and health care providers | Cross sectional study | No | English | No | eligible to participate if they resided in the United States, were at least 18 years old, had successfully completed 1000 or more human intelligence tasks (HITs), and had a HIT approval rate of 98% or higher | unable to provide consent |
| Focusing on Inattention: The Diagnostic Accuracy of Brief Measures of Inattention for Detecting Delirium | Annachiara Marra, MD, PhD, James C. Jackson, PsyD, E. Wesley Ely, MD, MPH, Amy J. Graves, MS, John F. Schnelle, PhD, Robert S. Dittus, MD, MPH, Amanda Wilson, MD, and Jin H. Han, MD, MSc | 2018 | Journal of Hospital Medicine | Manuscript | To determine the diagnostic accuracy of reciting the months of year backwards from December to July and December to January for delirium as diagnosed by a psychiatrist | Other clinical study | Secondary analysis | No, only ED | Cohort study; Other: prospective observational study | No | English | No | 65 years or older, not in a hallway bed, and in the ED for less than 12 hours | previously enrolled, deaf or blind, comatose, suffered from end-stage dementia, or were unable to complete all the study assessment |
| Occult Suicidality and Psychiatric Disease Among Emergency Department Patients with Low-acuity Chief Complaints | Stephen M. McBride, BS Valerie A. Braz, PhD Christopher W. Jones, MD | 2018 | Western Journal of Emergency Medicine | Manuscript | Investigate patient receptiveness to ED-based mental health screening and intervention | Other clinical study | Primary enrollment | No, only ED | Cross sectional study | No | English | No | 18 years or older and had an ESI triage score of 4 or 5 | suffered from dementia or other cognitive impairment, if they presented to the ED for treatment of an acute psychiatric emergency, if they were intoxicated, or if they were incarcerated |
| Social Relationships, Homelessness, and Substance Use Among Emergency Department Patients | Amanda Jurewicz, BA, Deborah K. Padgett, MA, PhD, MPH, Ziwei Ran, MSW, Donna G. Castelblanco, MBE, Ryan P. McCormack, MD, Lillian Gelberg, MD, Donna Shelley, MD, MPH, and Kelly M. Doran, MD, MHS | 2022 | SUBSTANCE ABUSE | Manuscript | Examines how social relationships can precipitate or ameliorate homelessness and the connection (if any) between substance use and social relationships among ED patients experiencing homelessness | Health equity/social EM | Secondary analysis | No, only ED | Qualitative | No | English | No | 18 years or older and had become homeless within the past six months | unable to provide informed consent (e.g., dementia, intoxicated), in police custody, psychologically distressed, or otherwise medically unfit (e.g., critically ill) |
| Opioid use during the six months following an emergency department visit for acute pain. A prospective cohort study | Benjamin W. Friedman, MD, MS; Lorena Abril Ochoa, MD; Farnia Naeem, BS; Hector R. Perez, MD, MS; Joanna L. Starrels, MD, MS; Eddie Irizarry, MD; Andrew Chertoff, MD; Polly E. Bijur, PhD; E. John Gallagher, MD | 2020 | Annals of Emergency Medicine | Manuscript | To determine the frequency of recurrent or persistent opioid use during the 6 months after the ED visit | Health equity/social EM | Primary enrollment | No, only ED | Cohort study | No | English; Spanish | No | adults aged 18 years and older with a chief complaint of acute pain from any cause who were discharged home from the ED and were given a prescription for an oral opioid | use of any analgesic greater than 10 days per month on average before the onset of acute pain, and any patients who required admission to the hospital |
| A Qualitative Study of "What Matters" to Older Adults in the Emergency Department | Cameron J. Gettel, MD, MHS Arjun K. Venkatesh, MD, MBA, MHS Hollie Dowd, BA Ula Hwang, MD, MPH Rockman F. Ferrigno, MD Eleanor A. Reid, MD, MSc Mary E. Tinetti, MD | 2022 | Western Journal of Emergency Medicine | Manuscript | Adapting the "What Matters" questions to the ED setting, we sought to describe the concerns and desired outcomes of both older adult patients seeking ED care and their treating clinicians | Other clinical study | Primary enrollment | No, only ED | Cross sectional study; Qualitative | No | English | No | age >70; ability to answer questions without the assistance of caregivers; and an ESI score of 3, 4, or 5 | a status of medically unfit (as determined by the treating clinician) or evidence of cognitive impairment |
| Observational study to understand interpreter service use in emergency medicine: Why the key may lie outside of the initial provider assessment | Natalie C Benda, Rollin J Fairbanks, D Jeffrey Higginbotham, Li Lin, Ann M Bisantz | 2019 | Emergency Medicine Journal | Manuscript | Better understand interpreter service use across  the continuum of care, | Health equity/social EM | Primary enrollment | No, only ED | Cross sectional study | No | Spanish | No | adult patients | unable to provide consent |
| Patient Views on Emergency Department Screening and Interventions Related to Housing | Audrey Kelly MD, Daniela Fazio, Deborah Padgett MA, PhD, MPH, Ziwei Ran MSW, Donna G. Castelblanco MBE, Diana Kumar MPH, Kelly M. Doran MD, MHS | 2021 | Academic Emergency Medicine | Manuscript | To better understand patient perspectives on ED-based screening and interventions related to housing instability, as a step toward improving future efforts | Health equity/social EM | Primary enrollment | No, only ED | Qualitative | No | English | No | 18 years or older, and had become homeless within the preceding 6 months (regardless of whether this was their first episode of homelessness) | intoxicated, psychiatrically or medically unstable, unable to provide informed consent, or in police or prison custody |
| Adolescents' Acceptance of Long-Acting Reversible Contraception After an Educational Intervention in the Emergency Department: A Randomized Controlled Trial | Tatyana Vayngortin, Lela Bachrach, Sima Patel, Kathleen Tebb | 2020 | Western Journal of Emergency Medicine | Manuscript | Assessed contraception use among adolescents in the ED and evaluated the impact of an educational  video on their interest in and uptake of LARCs | Other clinical study | Primary enrollment | No, only ED | RCT | Yes | English | Yes | Female patients 14 to 21 years old reporting prior sexual activity | critically ill, seeking care for a psychiatric chief complaint or sexual assault, not proficient in English, and/or currently using a LARC method |
| Geriatric assistive devices improve older patient engagement and clinical care in an emergency department | Naleef Fareed, Lauren T. Southerland, Brian M. Rao, Cynthia J. Sieck | 2021 | American Journal Emergency Medicine | Manuscript | To evaluate Geriatric Emergency Department equipment use and the impact on patient experience | Other clinical study | Primary enrollment | No, only ED | Cross sectional study; Qualitative | No | English | No | 65 years who had used any of the GED equipment | those in skilled nursing facilities or incarcerated |
| Anti-immigrant Rhetoric and the Experiences of Latino Immigrants in the Emergency Department | Carolina Ornelas, MPH Jacqueline M. Torres, PhD, MPH Jesus R. Torres, MD, MPH Harrison Alter, MD, MS Breena R. Taira, MD, MPH Robert M. Rodriguez, MD | 2021 | Western Journal of Emergency Medicine | Manuscript | To better understand Latino patients experiences and fears with regard to anti-immigrant rhetoric, immigration enforcement, and ED utilization | Health equity/social EM | Primary enrollment | No, only ED | Cross sectional study; Qualitative | No | English; Spanish | Does not say | Latino individuals | 1) trauma; 2) transfer from another facility; 3) inability to participate in an interview because of intoxication, altered mental status, or critical illness; 4) incarceration; and 5) on psychiatric hold |
| Impact of Social Determinants of Health, Health Literacy, Self-perceived Risk, and Trust in the Emergency Physician on Compliance with Follow-up | James Sutton, Leon Gu, Deborah B. Diercks | 2021 | Western Journal of Emergency Medicine | Manuscript | We hypothesized that a patient's social factors, health literacy, self-perceived risk, and trust in the emergency physician may impact follow-up compliance | Health equity/social EM | Primary enrollment | No, only ED | Cohort study | No | English | No | older than 18 years, and presented with chest pain, later determined to be low risk for ACS | pregnant patients, prisoners, homeless patients, or those with human immunodeficiency virus |
| Transportation Preferences of Patients Discharged from the Emergency Department in the Era of Ridesharing Apps | Amar Tomar, Siddhi S. Ganesh, John R. Richards | 2019 | Western Journal of Emergency Medicine | Manuscript | To determine the preferences of patients discharged from the ED with regard to their transportation home, and their awareness and past use of ridesharing services such as Lyft and Uber | Health equity/social EM | Primary enrollment | No, only ED | Cross sectional study | No | English | No | adult patients 18 years of age or older, or adult parents of patients under 18, who were about to be discharged from the ED | patients requiring ambulance transport home |
| Limited Ability of Three Health Literacy Screening Items to Identify Adult English- and Spanish-Speaking Emergency Department Patients With Lower Health Literacy | Roland C. Merchant, MD, ScD; Sarah J. Marks, MS; Melissa A. Clark, PhD; Michael P. Carey, PhD; Tao Liu, PhD | 2020 | Annals of Emergency Medicine | Manuscript | Investigated three health literacy screening items' ability to identify English- and Spanish-speaking adult emergency department patients with lower health literacy | Health equity/social EM | Secondary analysis | No, only ED | Cross sectional study | No | English; Spanish | No | 18 to 64 years-old; English or Spanish speaking; not critically ill or injured; not prison inmates, under arrest, or undergoing home confinement; not presenting for an acute psychiatric illness; and not intoxicated; and if they lacked a physical or cognitive impairment that prevented them from providing consent or participating in the study | HIV infected (because the parent study involved HIV testing), not remaining in the United States for the subsequent year (because the parent study involved follow-up), unable to provide a method of reaching them after enrollment |
| The Presence of Companions during Emergency Department Evaluation and its Impact on Perceptions of Clinician-Patient Communication | Talea Cornelius, PhD, Nathalie Moise, MD, MS, Jeffrey L. Birk, PhD, Donald Edmondson,  PhD, and Bernard P. Chang, MD, PhD | 2018 | Emergency Medicine Journal | Manuscript | Tested whether the presence of companions in the ED relate to stronger clinician-patient communication | Other clinical study | Secondary analysis | No, only ED | Cohort study | No | English; Spanish | No | > 18 years of age who presented to the ED with an admitting diagnosis of NSTEMI or UA | terminal non-cardiovascular illness with life expectancy < 1 year, severe mental illness, significant cognitive impairment, known significant alcohol or substance abuse, and unavailability for follow-up |
| A Qualitative Analysis of Patients' Perceptions of Shared Decision-Making in the Emergency Department: "Let me know I have a choice" | Elizabeth M. Schoenfeld, MD, MS; Sarah L. Goff, MD; Gwendolyn Downs, DO; Robert J. Wenger, DO; Peter K. Lindenauer, MD, MSc; Kathleen M. Mazor, EdD | 2018 | Academic Emergency Medicine | Manuscript | To explore the use of shared decision making from the perspectives of ED patients, focusing on what affects patients' desired level of involvement and what barriers and facilitators patients find most relevant to their experience | Other clinical study | Primary enrollment | No, only ED | Qualitative | No | English | No | clinically stable ED patients | hemodynamically stable, did not require acute interventions, and were not immediately leaving the department for testing, admission or discharge |
| Patient Preference for Pain Medication in the Emergency Department Is Associated with Non-fatal Overdose History | Lauren K. Whiteside, Jason Goldstick, Aaron Dora-Laskey, Laura Thomas, Maureen Walton, Rebecca Cunningham, Amy S.B. Bohnert | 2018 | Western Journal of Emergency Medicine | Manuscript | To determine if patient-reported preference for specific pain medications was associated with a history of lifetime overdose among patients seeking care in the emergency department | Other clinical study | Secondary analysis | No, only ED | Cross sectional study | No | English | No | 18-60 years old without regard for chief complaint | did not understand English; were in police or corrections custody; had cognitive or other impairment precluding ability to consent (e.g., visual or hearing impaired); were medically unstable requiring immediate resuscitation (e.g., from major injury or sepsis), or were presenting for evaluation and treatment of sexual assault or suicidal ideation |
| Adverse childhood experiences are associated with at-risk drinking, cannabis, and illicit drug use in females but not males: An Emergency Department study | Carol B. Cunradi, Raul Caetano, Harrison J. Alter & William R. Ponicki | 2020 | The American Journal of Drug and Alcohol Abuse | Manuscript | Understanding how ACEs are associated with substance use among urban ED patients | Health equity/social EM | Primary enrollment | No, only ED | Cross sectional study | No | English; Spanish | No | clinically stable ED patients | <18 years old |
| Can an Emergency Department Initiated Intervention Prevent Subsequent Falls and Healthcare Utilization in Older Adults? A Randomized Controlled Trial | Elizabeth M. Goldberg, MD, ScM; Sarah J. Marks, MS; Linda J. Resnik, PT, PhD; Sokunvichet Long, BS; Hannah Mellott, BA; Roland C. Merchant, MD, ScD | 2020 | Annals of Emergency Medicine | Manuscript | Determine whether an emergency department‚Äìinitiated fall-prevention intervention can reduce subsequent fall-related and all-cause ED visits and hospitalizations in older adults | Other clinical study | Primary enrollment | No, only ED | RCT | Yes | English; Spanish | No | 65 years and older were eligible to participate if they presented to the ED within 7 days of a fall and were being discharge home | altered mental status (eg, intoxicated), who were homeless, or could not provide a telephone number for follow-up |
| Acceptability of Contraceptive Services in the Emergency Department: A Cross-sectional Survey | Andreia B. Alexander, MD, PhD, MPH Kimberly Chernoby, MD, JD, MA Nathan VanderVinne, DO Yancy Doos Navneet Kaur Caitlin Bernard, MD Jeffrey A. Kline, MD | 2021 | Western Journal of Emergency Medicine: Integrating Emergency Care with Population Health | Manuscript | To determine the extent to which adult women of childbearing age who present to the ED would be receptive to receiving contraception and/or information about contraception in the ED + identify the barriers faced in attempting to obtain SRH care in the past | Other clinical study | Primary enrollment | No, only ED | Cross sectional study | No | English | No | women aged 18-50 in the ED setting | intoxicated, exhibiting hostile behavior, chief complaint of sexual assault |
| Patients' Perspectives on Emergency Department COVID-19 Vaccination and Vaccination Messaging Through Randomized Vignettes | Michael J. Waxman, Maile Ray, Elissa M. Schechter-Perkins, Kiran Faryar, Karen Coen Flynn, Mandi Breen, Susan M. Wojcik, Fiona Berry, Amy Zheng, Ashar Ata, E. Brooke Lerner, Michael S. Lyons, and Sandra McGinnis | 2022 | Public Health Reports | Manuscript | Prior to expanding COVID-19 vaccination to the acute care setting, we assessed ED patients‚Äô COVID-19 vaccine status, perspectives, and hypothetical receptivity to ED-based vaccination | Other clinical study | Primary enrollment | No, only ED | Cross sectional study | No | English | No | Adult patients aged >18 years were randomly selected from EDs in each of the 5 study sites | incarcerated, had limited English proficiency, were unable to complete the survey, were critically ill, or had other clinical circumstances limiting study involvement |
| Drinking Context: Specific Dose-Response Models of Intimate Partner Violence Among an Urban Emergency Department Sample | CAROL B. CUNRADI, m.p.H., pH.d., WILLIAM R. PONICKI, m.A, HARRISON J. ALTER, m.d., m.S., RAUL CAETANO, m.d., pH.d., CHRISTINA MAIR, m.p.H., pH.d., & JULIET LEE | 2020 | Journal of Studies on Alcohol and Drugs | Manuscript | aAssociation between physical intimate partner violence and frequencies of drinking and volume consumed | Health equity/social EM | Primary enrollment | No, only ED | Cross sectional study | No | English; Spanish | No | 18-50 years old; resident of the county in which the hospital is located; and married, cohabiting, or in a romantic (dating) relationship for the past 12 months | intoxicated, experiencing acute psychosis or suicidal or homicidal ideation, were cognitively/psychologically impaired and unable to provide informed consent, in custody by law enforcement, or in need of immediate medical attention |
| Use of Amphetamine-Type Stimulants Among Emergency Department Patients With Untreated Opioid Use Disorder | Marek C. Chawarski, PhD; Kathryn Hawk, MD, MHS; E. Jennifer Edelman, MD, MHS; Patrick O'Connor, MD, MPH; Patricia Owens, MS; Shara Martel, MPH; Edouard Coupet, Jr, MD, MS; Lauren Whiteside, MD; Judith I. Tsui, MD, MPH; Richard Rothman, MD; Ethan Cowan, MD; Lynne Richardson, MD; Michael S. Lyons, MD; David A. Fiellin, MD; Gail D'Onofrio, MD, MS | 2020 | American College of Emergency Physicians | Manuscript | Determine the effectiveness of an implementation facilitation strategy to increase the rate of ED-initiated buprenorphine with referral for ongoing addiction treatment in actual ED settings | Health equity/social EM | Secondary analysis | No, only ED | RCT; Clinical trial | Yes | English | No | English-speaking adults (18 years or older) for untreated opioid use disorder | unable to provide consent |
| Telemedicine Physical Examination Utilizing a Consumer Device Demonstrates Poor Concordance with In-Person Physical Examination in Emergency Department Patients with Sore Throat: A Prospective Blinded Study | Moneeb Akhtar, BS, Paul G. Van Heukelom, MD, Azeemuddin Ahmed, MD, MBA, Rachel D. Tranter, PA-C, Erinn White, PA-C, Nathaniel Shekem, PA-C, David Walz, PA-C, Catherine Fairfield, BSN, J. Priyanka Vakkalanka, ScM, and Nicholas M. Mohr, MD, MS | 2018 | Telemedicine and e-Health | Manuscript | Determine whether the physical exam in patients with pharyngitis correlates with an in-person examination. Additionally, we aimed to understand patient and provider comfort with telemedicine as an examination medium for sore throat, with the intention of validating that telemedicine provides noninferior information with high concordance to the in-person evaluation. | Other clinical study | Primary enrollment | No, only ED | Cross sectional study | No | English | No | age >18 years, presenting during day and evening shifts by a single research assistant | Prisoners, pregnant women, and those unable to provide consent |
| Potential of Mobile Health Technology to Reduce Health Disparities in Underserved Communities | Tara van Veen, Sophia Binz, Meri Muminovic, Kaleem Chaudhry, Katie Rose, Sean Calo, Jo-Ann Rammal, John France, Joseph B. Miller | 2019 | Western Journal of Emergency Medicine | Manuscript | To determine the readiness to use mHealth technology in underserved communities | Health equity/social EM | Primary enrollment | No, only ED | Cross sectional study; Qualitative | No | English | Yes | patients and parents of children presenting to the low-acuity section of the ED with chief complaints of sore throat, cough and congestion, non-traumatic headache, and symptoms of sexually transmitted infections | patients <2 years and >50 years old, severe illness with expected hospital admission, and inability to provide informed consent |
| Association of Social Needs and Housing Status Among Urban Emergency Department Patients | Kadia Wormley, Drusia Dickson, Harrison Alter, Ndidi Njoku, Partow Imani, Erik S. Anderson | 2022 | Western Journal of Emergency Medicine | Manuscript | People experiencing homelessness have high rates of social needs when presenting for emergency department (ED) services, but less is known about patients with housing instability who do not meet the established definitions of homelessness | Health equity/social EM | Primary enrollment | No, only ED | Cross sectional study | No | English; Spanish | No | >18 years who spoke English or Spanish and presented to the ED during study hours | medically unstable, unresponsive, had altered mental status precluding participation, or had already participated in the study |
| Predicting the transition to chronic pain six months after an ED visit for acute pain. A prospective cohort study | Benjamin W. Friedman, MD, MS, Lorena Abril, MD, Farnia Naeem, BS, Eddie Irizarry, MD, Andrew Chertoff, MD, Michael McGregor, MD, Polly E. Bijur, PHD, and E. John Gallagher, MD | 2020 | The Journal of Emergency Medicine | Manuscript | To determine how often acute pain transitions to chronic pain among patients in the ED and whether persistent pain 1 week after the ED visit was associated with chronic pain | Other clinical study | Primary enrollment | No, only ED | Cohort study | No | English; Spanish | No | 18 years and older, patients presenting to an ED for acute, new-onset pain for whom an opioid was prescribed on discharge from the ED | use of any analgesic > 10 days per month on average prior to the onset of acute pain and any patients who required admission to the hospital |
| Impacts of the Pandemic on Social Determinants of Health in an Academic Emergency Department | Shannon Findlay, MD, MPH Uche Okoro, MBBS, DrPH Sangil Lee, MD, MS Karisa Harland, MPH, PhD Marisa Evers, MD Elizabeth Gaffney, BS Mary McCormick, BA Chris Buresh, MD, MPH | 2022 | Western Journal of Emergency Medicine | Manuscript | Determine the effects of the COVID-19 pandemic on our patient population in the emergency department | Health equity/social EM | Primary enrollment | No, only ED | Cross sectional study | No | English; Spanish; French; Chinese (including Mandarin); Arabic; Other: Swahili | Yes | patients (or their guardians if the patient was <18 years of age), stable enough to give verbal consent and complete the form | unstable psychiatric or behavioral issues, altered mental status, or imprisonment status |
| Arrests Among High-Risk Youth Following Emergency Department Treatment for an Assault Injury | Patrick M. Carter, MD, Aaron D. Dora-Laskey, MD, MS, Jason E. Goldstick, PhD, Justin E. Heinze, PhD, Maureen A. Walton, MPH, PhD, Marc A. Zimmerman, PhD, Jessica S. Roche, MPH, Rebecca M. Cunningham, MD | 2018 | American Journal of Preventive Medicine | Manuscript | Characterize arrests among high-risk, assault-injured, drug-using youth following emergency department treatment | Health equity/social EM | Primary enrollment | No, only ED | Cohort study | No | English | No | youth (aged 18-24 years) with past 6-month drug use who were seeking emergency department treatment for either an assault or for non-violence reasons | presentation for sexual assault, suicidal ideation/attempt, child maltreatment, or cognitive conditions precluding consent (e.g., intoxication). Incarcerated youth (3.2%), unstable trauma patients were recruited if they stabilized within 72 hours |
| Prevalence and Predictors of Driving after Prescription Opioid Use in an Adult ED Sample | Aaron D. Dora-Laskey, MD, MS Jason E. Goldstick, PhD Brooke J. Arterberry, PhD Suni Jo Roberts, BS Rebecca L. Haffajee, JD, PhD, MPH Amy S.B. Bohnert, PhD, MHP Rebecca M. Cunningham, MD Patrick M. Carter, MD | 2020 | Western Journal of Emergency Medicine | Manuscript | Examined the prevalence and correlates of driving after taking prescription opioids among adults seeking ED treatment | Other clinical study | Primary enrollment | No, only ED | Cross sectional study | No | English | No | ages 25-60 | cognitively impaired by intoxication, illness, or injury; lacked adequate command of English; were in police or corrections custody; were presenting for evaluation and treatment of sexual assault or suicidal ideation; were classified by ED staff as a Level I trauma; or required special precautions due to the risk of infectious disease exposure |
| Shared Decision-making for Syncope in the Emergency Department: A Randomized Controlled Feasibility Trial | Marc A Probst, MD, MS, Michelle P. Lin, MD, MS, Jeremy Sze, BS, Erik P. Hess, MD MPH, Maggie Breslin, MDesc, Dominick L Frosch, PhD, Benjamin C. Sun, MD, MPP,  Marie-Noelle Langan, MD, Venkatesh Thiruganasambandamoorthy, MBBS, Lynne D. Richardson, MD | 2020 | Academic Emergency Medicine | Manuscript | To evaluate the feasibility of performing a randomized controlled trial of a shared decision-making tool for low-to-intermediate risk syncope patients presenting  to the ED | Other clinical study | Primary enrollment | No, only ED | RCT | Yes | English | No | >30 years who presented with syncope and were deemed appropriate for shared decision making by the treating attending physician | major communication barrier (e.g. significant visual or auditory impairment, altered mental status), lack of fixed address and telephone number, and a serious diagnosis identified in the ED, as determined by the treating clinician |
| Emergency department-based, nurse-initiated, serious illness conversation intervention for older adults: a protocol for a randomized controlled trial | Thidathit Prachanukool, Susan D. Block, Donna Berry, Rachel S. Lee, Sarah Rossmassler, Mohammad A. Hasdianda, Wei Wang, Rebecca Sudor, Mara A. Schonberg, James A. Tulsky, and Kei Ouchi | 2022 | Trials | Manuscript | To test the impact of ED GOAL administered by trained nurses on self-reported, advance care planning engagement after leaving the ED and to evaluate whether ED GOAL increases self-reported completion of serious illness conversation and other patient-centered outcomes | Other clinical study | Primary enrollment | No, only ED | RCT; Protocol | Yes | English | No | adults 50 years and older with serious life-limiting illness | clearly documented goals for medical care, including a serious illness conversation in the recent 3 months or a presenting medical order for life-sustaining treatment |
| Fidelity and feasibility of a brief emergency department intervention to empower adults with serious illness to initiate advance care planning conversations | Richard E. Leiter, MD, MA, Miryam Yusufov, PhD, Mohammad Adrian Hasdianda, MD, MSc, MMSc, Lauren A. Fellion, PA-C, Audrey C. Reust, PA-C, Susan D. Block, MD, James A. Tulsky, MD, and Kei Ouchi, MD, MPH | 2018 | Journal of Pain and Symptom Management | Manuscript | To assess the fidelity and feasibility of empowering patients to discuss advance care planning with their outpatient clinicians, intervention in a high-volume ED | Other clinical study | Primary enrollment | No, only ED | Cross sectional study; Qualitative | Yes | English | No | >65 years old with serious, life-limiting illness (metastatic cancer, oxygen-dependent chronic obstructive lung disease, chronic kidney disease on dialysis, New York Heart Association Stage 3 or 4 heart failure) | a medical order for life-sustaining treatment in the electronic health record, were determined by the treating ED clinicians to be inappropriate for this study (e.g., in acute physical or emotional distress), or could not provide informed consent |
| Depression in Emergency Department Patients and Association with Healthcare Utilization | David G. Beiser, MD, Charlotte E. Ward, MPH, Milkie Vu, MA, Neda Laiteerapong, MD, and Robert D. Gibbons, PhD | 2019 | Society for Academic Emergency Medicine | Manuscript | Estimate the rate and severity of major depressive disorder in a nonpsychiatric ED population and its association with subsequent ED visits and hospitalizations | Other clinical study | Primary enrollment | No, only ED | Cohort study | No | English | No | patients over the age of 18 years old presenting with nonpsychiatric chief complaints to the ED | patients with acute psychiatric complaints, patients triaged with ESI 1-2 |
| Impact of risk-based sexually transmitted infection screening in the emergency department | Fahd A. Ahmad, Kayleigh Fischer, Hongjie Gu, Thomas C. Bailey, Donna B. Jeffe, Christopher R. Carpenter, Philip R.O. Payne | 2022 | Academic Emergency Medicine | Manuscript | Determine whether an electronic, risk-based sexually transmitted infection screening program in our ED was associated with an increase in STI testing among  at-risk adolescents. | Health equity/social EM | Primary enrollment | No, only ED | Cohort study | Yes | English | Yes | All patients 15-21 years of age | disabled and could not independently use a tablet computer, severely ill |
| An Assessment of the Social Determinants of Health in an Urban Emergency Department | Edgardo Ordonez, MD, MPH Katherine Dowdell, MD Natasha M. Navejar, BS Deepa Dongarwar, MS Aya Itani, MD, MPH Hamisu M. Salihu, MD, PhD | 2021 | Western Journal of Emergency Medicine | Manuscript | Administered a social needs screening tool and needs  assessment survey to assess SDOH and evaluate for trends in the population of patients visiting our ED | Health equity/social EM | Primary enrollment | No, only ED | Cross sectional study | No | English; Spanish; Other: translated survey and used phone interpreters | No | Patients recruited for the survey were registered in the ED of a large, urban safety-net hospital located in Houston, Texas | under the age of 18, with 1:1 sitters, and incarcerated individuals, pregnant patients > 20 weeks |
| Comparison of the Safety Planning Intervention With Follow-up vs Usual Care of Suicidal Patients Treated in the Emergency Department | Barbara Stanley, Gregory K Brown, Lisa A Brenner, Hanga C Galfalvy, Glenn W Currier, Kerry L Knox, Sadia R Chaudhury, Ashley L Bush, and Kelly L Green | 2018 | JAMA Psychiatry | Manuscript | Can a brief suicide prevention intervention reduce suicidal behaviors and improve treatment engagement among patients who present to the emergency department for suicide-related concerns? | Other clinical study | Primary enrollment | No, only ED | Cohort study | Yes | English | No | 18 years or older; ED visit for a suicide-related concern | not getting admitted |
| Drinking and Intimate Partner Violence Severity Levels Among U.S. Ethnic Groups in an Urban Emergency Department | Raul Caetano, MD, PhD, Carol B. Cunradi, MPH, PhD, Harrison J. Alter, MD, MS, Christina Mair, PhD, and Rebecca K. Yau, PhD | 2019 | Society for Academic Emergency Medicine | Manuscript | Ethnic-specific 12-month rates of physical IPV by severity and their association with drinking and other sociodemographic and personality correlates in an urban ED sample | Health equity/social EM | Primary enrollment | No, only ED | Cross sectional study | No | English; Spanish | No | 18 to 50 years old; residence in the county where the study was conducted; and married, cohabiting, or in a romantic (dating) relationship for the past 12 months | intoxicated, experiencing acute psychosis or suicidal or homicidal ideation, were cognitively and/or psychologically impaired and unable to provide informed consent, in custody by law enforcement, or in need of immediate medical attention |
| Cross-sectional Analysis of Food Insecurity and Frequent Emergency Department Use | Alex Estrella, MD Joy Scheidell, PhD Maria Khan, PhD Donna Castelblanco, MBE Tod Mijanovich, PhD David C. Lee, MD, MS Lillian Gelberg, MD, MSPH Kelly M. Doran, MD, MS | 2021 | Western Journal of Emergency Medicine | Manuscript | Association between multiple measures of food insecurity and frequent ED use in a random sample of ED patients | Health equity/social EM | Secondary analysis | No, only ED | Cross sectional study | No | English; Spanish | No | >18 years old, medically/psychiatrically stable, not in prison/police custody | Most common reasons for ineligibility were being medically unstable, intoxicated, not speaking English/Spanish, or in police/prison custody |
| Social Disconnection Among Older Adults Receiving Care in the Emergency Department | Deepika Kandasamy, MPH Timothy F. Platts-Mills, MD, MSc Manish N. Shah, MD, MPH Kim A. Van Orden, PhD Marian E. Betz, MD, MPH | 2018 | Western Journal of Emergency Medicine | Manuscript | Describes the prevalence of social disconnection and patient interest in social resources to address social disconnection among older adults receiving emergency department care | Health equity/social EM | Primary enrollment | No, only ED | Cross sectional study | No | English | No | >65 years on the ED tracking board and asked treating providers to confirm eligibility (medically able to participate | institutionalized [e.g., prisoners, nursing home residents]) |
| Vitamin D Deficiency and Long-Term Cognitive Impairment Among Older Adult Emergency Department Patients | Christopher S. Evans, MD, MPH Wesley Self, MD, MPH Adit A. Ginde, MD, MPH Rameela Chandrasekhar, PhD E. Wesley Ely, MD, MPH Jin H. Han, MD, MSc | 2019 | Western Journal of Emergency Medicine | Manuscript | Vitamin D deficiency at ED presentation was associated with long-term cognitive impairment in older adults | Other clinical study | Secondary analysis | No, only ED | Cohort study | No | English | No | 65 years or older and in the ED for less than four hours at the time of enrollment | previously enrolled; deaf, comatose, non-verbal or unable to follow simple commands prior to their current illness; were considered unsuitable for enrollment by the treating physician or nurse; were unavailable for enrollment within the four-hour time limit secondary to clinical care (eg, procedures, radiologic testing, etc,); or were discharged home from the ED |
| Obtaining history with a language barrier in the emergency department: Perhaps not a barrier after all | Megan Litzau, MD Joseph Turner, MD Katie Pettit, MD Zachary Morgan, PhD Dylan Cooper, MD | 2018 | Western Journal of Emergency Medicine | Manuscript | Compared the medical histories obtained by physicians in the emergency department (ED) based on whether the patients primarily spoke English or Spanis | Health equity/social EM | Primary enrollment | No, only ED | Cross sectional study; Other: prospective, observational study | No | English; Spanish | Does not say | English or Spanish speaking, >18yo | unable to provide consent |
| Food Insecurity and Insulin Use in Hyperglycemic Patients Presenting to the Emergency Department | Heng Ky Nhoung, BA Munish Goyal, MD Maria Cacciapuoti, BA Hannah Day, BS Taymour Hashemzadeh, BS Michelle Magee, MD Yumi S. Jarris, MD | 2020 | Western Journal of Emergency Medicine | Manuscript | Examined the prevalence of food insecurity and subtherapeutic insulin use among patients who presented to the ED with a blood glucose level of greater than 250 milligrams per deciliter | Health equity/social EM | Primary enrollment | No, only ED | Cross sectional study | No | English | No | blood glucose > 250 milligrams per deciliter (mg/dL), medical stability | Non-English speakers |
| Patient and Community Organization Perspectives on Accessing Social Resources from the Emergency Department: A Qualitative Study | Margaret E. Samuels-Kalow, MD, MSHP Melanie F. Molina, MD Gia E. Ciccolo, MPH Alexa Curt, BA Emily C. Cleveland Manchanda, MD, MPH Nicole C. de Paz, MD Carlos A. Camargo Jr., MD, DrPH | 2020 | Western Journal of Emergency Medicine | Manuscript | Examined the perspectives of patients and community organizations regarding social risk screening and linkage from the ED | Health equity/social EM | Primary enrollment | No, only ED | Qualitative | No | English; Spanish | Yes | adults or parents/guardians of pediatric patients, were expected by the clinical team to be discharged at the conclusion of their ED visit | involuntary mental health hold or with active intoxication |
| "It wasn't just one thing": A Qualitative Study of Newly Homeless Emergency Department Patients | Kelly M. Doran, MD, MHS, Ziwei Ran, MSW, Donna Castelblanco, MBE, Donna Shelley, MD, MPH, and Deborah K. Padgett, MA, PhD, MPH | 2019 | Society for Academic Emergency Medicine | Manuscript | To provide interventions in the ED for homelessness that attempt to prevent it before it occurs | Health equity/social EM | Primary enrollment | No, only ED | Cross sectional study; Qualitative | No | English | No | 18 years or older with a new-onset homelessness (defined as living in a shelter or on the streets) episode in the past 6 months | too intoxicated to provide consent, otherwise medically unfit (e.g., critically ill), psychologically distressed, in police or prison custody, or could not understand study consent |
| Family Identification of Delirium in the Emergency Department in patients with and without Dementia: Validity of the FAM-CAM | Tanya Mailhot, PhD, Chad Darling, MD MSc, Jillian Ela, BS, Yelena Malyuta, BS, Sharon K Inouye, MD MPH, Jane Saczynski, PhD | 2020 | Journal of the American Geriatric Society | Manuscript | To examine the ability of the family-rated Family-Confusion Assessment Method to identify delirium in the Emergency Department among patients with and without dementia, as compared to the reference-standard Confusion Assessment Method | Other clinical study | Primary enrollment | No, only ED | Cross sectional study | No | English | No | receiving care in the ED, be 70 years or older, have a family caregiver present in the ED | presented to the ED for head trauma because altered mental status due to head trauma confounds the typical diagnosis of delirium and has a different trajectory and treatment approach |
| PTSD Symptoms and Acute Pain in the Emergency Department: The Roles of Vulnerability and Resilience Factors among Low-Income, Inner-City Women | Teresa A. Lillis, PhD, John Burns, PhD, Frances Aranda, PhD, Yanina A. Purim-Shem-Tov, MD, Stephen Bruehl, PhD, Jean C. Beckham, PhD, and Stevan E. Hobfoll, PhD | 2018 | The Clinical Journal of Pain | Manuscript | Investigated direct and indirect relationships of PTSD symptoms, vulnerability factors (ie, resource loss, depressive symptoms, and social undermining), and resilience factors (ie, optimism, engagement, and social support) to acute pain reports in a sample of low-income, inner-city women | Health equity/social EM | Primary enrollment | No, only ED | Cohort study; Qualitative | No | Did not specify languages recruited | No | (1) female; (2) 18 to 40 years old; (3) premenopausal; (4) able to read and write English sufficiently to provide informed consent, and (5) presented to our institution's ED reporting acute pain of the chest, abdomen/pelvis, neck/shoulder, or back (ie, not extremity or head pain) | (1) pain intensity or any injury or illness great enough to impair concentration or capacity to understand study instructions or the nature of being in the study; (2) current chronic illness that involved constant or frequent pain; (3) history of chronic pain on presentation in ED or documented in the electronic medical record (EMR); (4) appearing intoxicated or under the influence of drugs at the ED visit; (5) self-reported or EMR-documented daily opiate use over the previous 3 months; or (6) the presenting ED pain was due to a traumatic circumstance (eg, a motor vehicle accident [MVA]), physical assault, sexual assault) |
| An Automated Tobacco Cessation Intervention for Emergency Department Discharged Patients | David T. Chiu, MD MPH Ronald Lavoie, MD Larry A. Nathanson, MD Leon D. Sanchez, MD MPH | 2021 | Western Journal of Emergency Medicine | Manuscript | Evaluate the effectiveness of an automated discharge instruction system in increasing the frequency of discharging current tobacco users with instructions for tobacco cessation | Other clinical study | Primary enrollment | No, only ED | Non randomized experimental study | Yes | Did not specify languages recruited; Other: they divided results into English/non-English so possible they included non-English speaking participants | Yes | All patients who were discharged from the ED during the study period were enrolled | not properly discharged were excluded including admitted, left against medical advice, eloped, expired, transferred, or left without being seen |
| Insurance reimbursement of emergency department dispensed naloxone kits | Sara Lookabill, Sebastian Hamilton, Natalija Farrell | 2019 | CLINICAL TOXICOLOGY | Abstract | Studying a novel pathway to dispense the naloxone from the ED with retrospective insurance reimbursement submission | Health equity/social EM | Primary enrollment | No, only ED | Cross sectional study | Yes | Did not specify languages recruited | Does not say | opioid use | unable to provide consent |
| Attitudes toward fentanyl test strips as a harm reduction strategy among emergency department patients who have used opioids in the past year | Sarah Putnam, Dominique Spence, Minh Le,  Alexandra Goslow, Lynne Fullerton, Silas Bussmanna, Brandon J Warrick | 2019 | Clinical Toxicology | Abstract | Describe a population of non-prescription opioid users; measure attitudes about potential fentanyl exposure; and assess openness to acquiring fentanyl test strips from emergency departments | Health equity/social EM | Primary enrollment | No, only ED | Cross sectional study | No | English | No | convenience sample of patients age 18 years and older, self-reported using non-prescription opioids in the past year | incarcerated patients |
| Brain-Based Biotypes of Psychiatric Vulnerability in the Acute Aftermath of Trauma | Jennifer S. Stevens, Ph.D., et al | 2021 | The American Journal of Psychiatry | Manuscript | Multisite transdiagnostic longitudinal study of  trauma exposure and related mental health outcomes to identify neurobiological predictors of risk, resilience, and different symptom presentations | Other clinical study | Primary enrollment | No, only ED | Cohort study | Yes | English | No | patients within 72 hours of trauma exposure, ages 18-75, able to speak and read English, oriented to time and place, and physically able to use a smartphone, and they had possessed a smartphone for more than 1 year | a solid organ injury greater than grade 1 or a significant hemorrhage, required a chest tube or general anesthesia, or were likely to be admitted for >72 hours |
| Bundled HIV and Hepatitis C Testing in the Emergency Department: A Randomized Controlled Trial | Ethan Cowan, MD, MS Heather Herman, BS Sara Rahman, BA Jennifer Zahn, BS Jason Leider, MD, PhD Yvette Calderon, MD, MS | 2018 | Western Journal of Emergency Medicine | Manuscript | Measure the impact of bundled HIV and HCV testing vs. HIV testing alone on test acceptance and identification of HCV and HIV | Health equity/social EM | Primary enrollment | No, only ED | RCT | Yes | English; Spanish | No | 18 years of age or older | medically unstable as determined by their ED provider, unable to consent, did not speak Spanish or English, were known to be HIV and/or HCV positive, or had been tested for HIV/HCV in the prior six months |
| Patient versus Physician Perceptions of Frailty: A Comparison of Clinical Frailty Scale Scores of Older Adults in the Emergency Department | Scott M. Dresden, MD, MS, Timothy F. Platts-Mills, MD, MSc, Deepika Kandasamy, Lauren Walden, and Marian E. Betz, MD, MPH | 2019 | Society for Academic Emergency Medicine | Manuscript | If emergency physicians and older adults differ in their perceptions of frailty and if those perceptions of frailty were related to disposition from the ED | Other clinical study | Secondary analysis | No, only ED | Cross sectional study | No | English | No | community dwelling older adults 65 years old and older | medically and cognitively able to participate and had not previously participated in this study |
| Development and Validation of a Measure to Assess Patients' Threat Perceptions in the Emergency Department | Talea Cornelius, PhD, MSW, Sachin Agarwal, MD, MPH, Othanya Garcia, William Chaplin, PhD, Donald Edmondson, PhD, MPH, and Bernard P. Chang, MD, PhD | 2018 | Society for Academic Emergency Medicine | Manuscript | Reports the development and validation of a seven-item measure of ED threat perceptions in patients evaluated for ACS | Other clinical study | Primary enrollment | No, only ED | Cohort study | No | English; Spanish | No | 18+ years of age who presented to the NYP ED with an admitting diagnoses of NSTEMI or UA | 1) terminal noncardiovascular illness with life expectancy < 1 year, 2) severe mental illness requiring urgent psychiatric hospitalization or intervention, 3) significant cognitive impairment, 4) known alcohol or substance abuse that would impede ability to complete study protocol, and 5) unavailable for follow-up |
| Patient Preferences regarding Shared Decision-making in the Emergency Department: Findings from a multi-site survey | Elizabeth M. Schoenfeld, MD, MS; Hemal K. Kanzaria, MD, MS; Denise D. Quigley, PhD; Peter St Marie, BS; Nikita Nayyar, DO; Sarah H. Sabbagh, MPH; Kyle L. Gress, BS, and Marc A. Probst, MD, MS | 2018 | Academic Emergency Medicine | Manuscript | To determine patients' desired level of involvement in medical decisions and their perceptions of potential  barriers and facilitators to shared decision making in the ED | Other clinical study | Primary enrollment | No, only ED | Cross sectional study | No | English | No | adult ED patients | altered mental status (e.g. intoxication or delirium), hemodynamic instability, acute psychiatric condition, dementia, hearing impairment, or other cognitive impairment |
| Counseling on Access to Lethal Means-Emergency Department (CALM-ED): A Quality Improvement Program for Firearm Injury Prevention | Kristen L. Mueller, MD Sonya Naganathan, MD, MSBS Richard T. Griffey, MD, MPH | 2020 | Western Journal of Emergency Medicine | Manuscript | To evaluate the feasibility of the Counseling on Access to Lethal Means intervention in the Emergency Department (CALM-ED) by non-physician personnel | Other clinical study | Primary enrollment | No, only ED | Cohort study; Other: prospective QI study | No | English | No | 18 years or older; had nursing-assigned triage chief complaints of suicidal ideation, suicidal attempt, or depression; had been placed on suicidal elopement precautions; had access to a telephone; and were ultimately discharged from the ED | admitted to the hospital, actively psychotic, or refused the intervention |
| Improving Understanding of Screening Questions for Social Risk and Social Need Among Emergency Department Patients | Gia Ciccolo, MPH, Alexa Curt, BA, Carlos A. Camargo Jr, MD, DrPH, Margaret Samuels-Kalow, MD, MPhil, MSHP | 2020 | Western Journal of Emergency Medicine | Manuscript | Create a brief social screening tool for social risks to use in EDs | Health equity/social EM | Primary enrollment | Other: interviews with community health workers | Cross sectional study; Qualitative | Yes | English; Spanish | Yes | adult patients or parent/guardians of pediatric patients, fluency in either English or Spanish, provider approval for approach, and plans for discharge home | involuntary mental health hold or with active intoxication |
| Tracking Assault-Injured, Drug-Using Youth in Longitudinal Research: Follow-Up Methods | Jessica S. Roche, MPH, Michael J. Clery, MD, MPP, Patrick M. Carter, MD, Aaron Dora-Laskey, MD, MS, Maureen A. Walton, MPH, PhD, Quyen M. Ngo, PhD,  Rebecca M. Cunningham, MD | 2018 | Academic Emergency Medicine | Manuscript | To describe the methods for retention in youth violence studies and the characteristics of hard-to-reach  participants | Health equity/social EM | Secondary analysis | No, only ED | Cohort study | No | English | Yes | 14-24 years-old seeking care for an assault injury | not able to provide informed consent (e.g., altered mental status, psychosis), presented for child abuse, acute sexual assault, or suicidal ideation/attempt) |
| Increased Sensitivity of Focused Cardiac Ultrasound for Pulmonary Embolism in Emergency Department Patients With Abnormal Vital Signs | James I. Daley, MD, MS, MPH, Kristin H. Dwyer, MD, Zachary Grunwald, MD, Daniel L. Shaw, MD, Michael B. Stone, MD, Alexandra Schick, MD, Michael Vrablik, DO, M. Kennedy Hall, MD, MS, Jane Hall, PhD, Andrew S. Liteplo, MD, Rachel M. Haney, MD, Nancy Hun, MD, Rachel Liu, MD, and Chris L. Moore, MD | 2019 | SOCIETY FOR ACADEMIC EMERGENCY MEDICINE | Manuscript | In patients with tachycardia or hypotension, the sensitivity of FOCUS for PE would increase substantially | Other clinical study | Primary enrollment | No, only ED | Cohort study | No | English | No | adults (18 years of age or older) with tachycardia and/or hypotension undergoing CTA for evaluation of possible PE in the ED | prisoners, wards of the state, and those where investigators could not obtain any echocardiographic data due to technical challenges |
| Implementation and Preliminary Results of an Emergency Department Nontargeted, Opt-Out Hepatitis C Virus Screening Program | Elissa M Schechter-Perkins, MD, MPH, Nancy S Miller, MD, Jon Hall, MPH, Joshua J Hartman, MPH, David Dorfman, MD, Chris Andry, PhD, Benjamin Linas, MD | 2018 | Academic Emergency Medicine | Manuscript | A novel nontargeted, opt-out HCV screening and linkage-to-care program implemented in an urban ED | Health equity/social EM | Primary enrollment | No, only ED | Cohort study; Other: descriptive analysis | Yes | English; Spanish; Portuguese ; French; Creole; Chinese (including Mandarin); Arabic; Vietnamese ; Korean; Russian | Yes | opt-out HCV screening to all ED patients over 13 years of age who are undergoing phlebotomy for any clinical purpose | 1) patients that receive primary care at an outside medical system that is known to treat HCV, 2) incarcerated persons, 3) pregnant women (until after pregnancy has ended), 4) individuals with viral load<600 IU/mL, 5) those with EMR notes documenting physician opted not to treat at this time, and 6) no contact information in the EMR |
| Feasibility of Health Literacy Tools for Older Patients in the Emergency Department | Matthew J. McGuinness, BS Joshua Bucher, MD James Karz, DO Carla Pardee, PA-C Laryssa Patti, MD Pamela Ohman-Strickland, PhD Jonathan V. McCoy, MD | 2020 | Western Journal of Emergency Medicine | Manuscript | Evaluates the feasibility of using a volunteer research associate to administer two separate health literacy assessment tools in the emergency department,  specifically in an older population of patients | Health equity/social EM | Primary enrollment | No, only ED | Cross sectional study | No | English; Spanish | No | 55 years of age or older | patients 90 years of age or older; any patient deemed to be under significant distress by their attending physician; prisoners; and patients who had an altered mental status for any reason |
| Two-Item Fall Screening Tool Identifies Older Adults at Increased Risk of Falling after Emergency Department Visit | Christopher J. Solie, DO Morgan B. Swanson, BS Kari Harland, MPH, PhD Christopher Blum, BS Kevin Kin, BS Nicholas Mohr, MD, MS | 2020 | Western Journal of Emergency Medicine | Manuscript | To externally validate Tiedemann et al's two-item, ED-specific fall screening tool and test handgrip strength to determine their ability to predict future falls. | Other clinical study | Primary enrollment | No, only ED | Cohort study | Yes | English | No | 65 years of age or older and were treated in the ED between 9 am and 11:59pm on weekdays and 2pm and 10pm on weekends | patients currently living in a nursing home, prisoners, patients with limited English-language skills, and those without the capacity to provide informed consent |
| Patient Experiences With Miscarriage Management in the Emergency and Ambulatory Settings | Carolyn A. Miller, MD, Andrea H. Roe, MD, MPH, Arden McAllister, MPH, Zachary F. Meisel, MD, MSHP, Nathanael Koelper, MPH, and Courtney A. Schreiber, MD, MPH | 2019 | OBSTETRICS & GYNECOLOG | Manuscript | To quantitatively and qualitatively describe the patient experience for clinically stable patients presenting with miscarriage to the emergency department or ambulatory clinics | Other clinical study | Secondary analysis | Other: ambulatory clinics | Cohort study | No | English | No | at least 18 year old, had ultrasound diagnosis of an anembryonic gestation or embryonic or fetal demise in the first trimester (5-12 completed weeks of gestation) confirmed by two clinicians, and closed cervical os | clinically unstable |
| A multi-faceted intervention to improve patient knowledge and safe use of opioids: Results of the ED EMC2 randomized controlled trial | Danielle M. McCarthy, MD, MS, Laura M. Curtis, MS, D. Mark Courtney, MD, MS, Kenzie A. Cameron, PhD, MPH, Patrick M. Lank, MD, MS, Howard S. Kim, MD, MS, Lauren A. Opsasnick, MS, Abbie E. Lyden, PharmD, Stephanie J. Gravenor, MBA, Andrea M. Russell, MS, Morgan R. Eifler, BS, Scott I. Hur, MPH, Megan E. Rowland, MPH, Surrey M. Walton, PhD, Enid Montague, PhD, Kwang-Youn A. Kim, PhD, and Michael S. Wolf, PhD, MPH | 2019 | Society for Academic Emergency Medicine | Manuscript | To evaluate the effect of an Electronic Medication Complete Communication (EMC2) Opioid Strategy on patients' safe use of opioids and knowledge about opioids | Health equity/social EM | Primary enrollment | No, only ED | RCT | Yes | English | No | 18 years of age or older, prescribed a tablet form of hydrocodone-acetaminophen (nonliquid formulation), responsible for self-administering their own medication, and were discharged by a provider who consented to the study | clinically unstable, psychologically impaired or intoxicated as judged by the research staff or ED provider, chronically taking opioids (as defined by self-report of "Daily or near daily" use of opioids for the past 90 days), being admitted to the hospital, unable to complete study follow-up, or pregnant |
| Advance Care Planning for Emergency Department Patients With COVID-19 Infection: An Assessment of a Physician Training Program | Martin F. Casey, MD, MPH, Laiken Price, MS, Daniel Markwalter, MD, Tommy Bohrmann, PhD, Tamy Moraes Tsujimoto, MS, Kyle Lavin, MD, MPH, Laura C. Hanson, MD, MPH, Feng-Chang Lin, PhD and Timothy F. Platts-Mills, MD, MSc | 2022 | American Journal of Hospice & Palliative Medicine | Manuscript | Determine the effect of an educational program for emergency physicians on advanced care planning conversations in the ED during the COVID-19 pandemic | Other clinical study | Primary enrollment | No, only ED | Non randomized experimental study | Yes | English | No | all adult patients (>18 years old) with a confirmed diagnosis of COVID-19 based on a positive polymerase chain reaction test in the 2 weeks up to and including the ED encounter of interest | unable to provide consent |
| Does Shared Decision Making Actually Occur in the Emergency Department? Looking at It from the Patients' Perspective | Elizabeth M. Schoenfeld, MD, MS, Marc A. Probst, MD, MS, Denise D. Quigley, PhD, Peter St. Marie, Nikita Nayyar, DO, Sarah H. Sabbagh, MPH, Tanesha Beckford, Hemal, K. Kanzaria, MD, MS | 2019 | Academic Emergency Medicine | Manuscript | To assess the frequency, content, and quality of shared decision making in the ED from patients‚Äô perspectives | Other clinical study | Primary enrollment | No, only ED | Cross sectional study | No | English | No | age 18 and older, alert, oriented, and hemodynamically stable | altered mental status; intoxication; presenting for hallucinations or suicidal or homicidal ideation; or other cognitive, hearing |
| Engaging hospital patients in the medication reconciliation process using tablet computers | Jennifer E. Prey, Fernanda Polubriaginof, Lisa V. Grossman, Ruth Masterson Creber, Demetra Tsapepas, Rimma Perotte, Min Qian, Susan Restaino, Suzanne Bakken, George Hripcsak, Leigh Efird, Joseph Underwood, and David K. Vawdrey | 2018 | Journal of the American Medical Informatics Association | Manuscript | A pilot study to determine whether patients‚Äô use of an electronic home medication review tool could improve medication safety during hospitalization | Other clinical study | Primary enrollment | No, only ED | RCT | Yes | English | No | We included English-speaking adult patients aged 18 years or older | history of cognitive impairment and acutely ill patients unable to participate in the study |
| Validity of the self-reported domains of subjective extent of nonadherence (Dose-nonadherence) scale in comparisonwith electronically monitored adherence to cardiovascular medications | Talea Cornelius, Corrine I Voils, Redeana C Umland, Ian M Kronish | 2019 | Patient Preference and Adherence | Manuscript | Assess the convergent validity of the self-reported Domains of Subjective Extent of Nonadherence (DOSE-Nonadherence) scale with electronically measured adherence to a single cardiovascular medication | Other clinical study | Secondary analysis | No, only ED | Cohort study | No | English; Spanish | No | presentation to the ED of a tertiary care hospital with a suspected ACS | any cognitive or psychiatric impairment that would impede study participation |
| Health-Related Social Needs Among Emergency Department Patients With HIV | Evan Gerber, Lillian Gelberg, Ethan Cowan, Tod Mijanovich, Donna Shelley, Rajneesh Gulati, Ian Wittman, Kelly M. Doran | 2020 | AIDS and Behavior | Manuscript | Examined the health-related social needs of ED patients who have HIV | Health equity/social EM | Primary enrollment | No, only ED | Cross sectional study | No | English; Spanish | No | >18 years old, could provide informed consent, were not in prison/police custody, and were medically and psychiatrically stable | unable to provide consent |
| Disparities in Acute Pain Treatment by Cognitive Status in Older Adults With Hip Fracture | Andrew K. Chang, MD MS, Robert R. Edwards, PhD, R. Sean Morrison, MD, Charles Argoff, MD, Ashar Ata, PhD, Christian Holt, MS, JD, and Polly E. Bijur, PhD | 2020 | The Gerontological Society of America | Manuscript | Examined the disparities in emergency department (ED) pain treatment based on cognitive status in older adults with an acute hip fracture | Other clinical study | Primary enrollment | No, only ED | Cohort study; Cross sectional study | No | English; Spanish | No | aged 65 years and older and presented to the ED for hip fracture | multiple trauma, bilateral hip fracture, or were transferred from another institution for management of the hip fracture |
| Assessment of Interpretation Modality and Patient Comprehension in Spanish-speaking Limited English Proficiency (LEP) Patients Presenting to the Emergency Department | Cassidy Muir, Amaury Michel, Cara Joyce, Theresa Nguyen | 2021 | Journal of Health Care for the Poor and Underserved | Manuscript | To assess interpretation modalities used in the emergency department in terms of patient satisfaction and understanding of discharge diagnosis | Health equity/social EM | Primary enrollment | No, only ED | Cross sectional study | No | Spanish | No | adult Spanish-speaking LEP patient who presented to the ED between 8 a.m. and 5 p.m. on Monday through Friday | patients whose chief complaint involved a psychiatric condition, those who were unable to give consent (i.e., intoxicated or comatose patients), those previously approached for the study, patients under the age of 18, or those presenting as a trauma activation |
| Effect of Two Tourniquet Techniques on Peripheral Intravenous Cannulation Success: A Randomized Controlled Trial | Theresa Tran, Sarah R. Lund, Micah D. Nichols, Tobias Kummer | 2019 | American Journal of Emergency Medicine | Manuscript | Tto determine whether elastic tourniquets or blood pressure cuffs are superior for success on the first attempt | Other clinical study | Primary enrollment | No, only ED | RCT | Yes | Did not specify languages recruited | No | convenience sample of adult patients who underwent PIV cannulation as part of their treatment in the ED | younger than 18 years, prison inmates, pregnant, unable to give informed consent, or critically ill and needed emergent IV access as determined by the ED clinician |
| A randomized, double-dummy, emergency department-based study of greater occipital nerve block with bupivacaine versus intravenous metoclopramide for treatment of migraine | Benjamin W. Friedman, MD, Eddie Irizarry, MD, Andrew Williams, MD, Clemencia Solorzano, PharmD, Eleftheria Zias, RPh, Matthew S. Robbins, MD, Melissa A. Harrilal, PA-C, Michael Del Valle, MD, Polly E. Bijur, PhD, E. John Gallagher | 2020 | American Headache Society | Manuscript | To determine whether greater occipital nerve blocks was as effective as intravenous metoclopramide for migraine | Other clinical study | Primary enrollment | No, only ED | RCT | Yes | English; Spanish | No | adults age >18 years who presented to the ED with an acute headache of moderate or severe intensity that met International Classification of Headache Disorders, 3rd edition migraine criteria | if informed consent could not be obtained, if there was concern for a secondary cause of headache, for contra-indications to GONB, prior treatment with GONB at any time previously, or allergy or contra-indication to the investigational medications, pregnant and lactating patients |
| Supratherapeutic Psychotropic Drug Levels in the Emergency Department and Their Association with Delirium Duration: A Preliminary Study | Jin H. Han, MD, MSc, Alex Chen, MD, Eduard E. Vasilevskis, MD, MPH, John F. Schnelle, PhD, E. Wesley Ely, MD, MPH, Rameela Chandrasekhar, PhD, Ryan D. Morrison, BA, Timothy P. Ryan, PhD, J. Scott Daniels, PhD, Jeff J. Sutherland, PhD, and Sandra F. Simmons, PhD | 2019 | Journal of the American Geriatrics Society | Manuscript | To determine the frequency of supratherapeutic psychotropic drug levels in older hospitalized patients and if it is associated with the duration of emergency department delirium | Other clinical study | Secondary analysis | No, only ED | Cohort study | No | English | No | 65 years or older, in the ED for less than 4 hours at the time of enrollment, and unlikely to be discharged home | previously enrolled, deaf, comatose, nonverbal, or unable to follow simple commands before their current illness or were considered unsuitable for enrollment by the treating physician or nurse |
| Asymptomatic bacteriuria vs symptom underreporting in older Emergency Department patients with suspected urinary tract infection | Jeffrey M Caterino, Julie A Stephens, Carlos A Camargo Jr, Randell Wexler, Courtney Hebert, Lauren T Southerland, Katherine M Hunold, David S Hains, Jason J Bischof, Lai Wei, Alan J Wolfe, Andrew Schwaderer | 2020 | Journal of American Geriatric Society | Manuscript | To investigate the accuracy of chart abstraction versus direct ED patient interview for the presence of GU symptoms | Other clinical study | Secondary analysis | No, only ED | Cross sectional study | No | English | No | ED adults aged 65 and older who had a urinalysis ordered for clinical care | chronic or intermittent catheterization, recent UTI or positive urine culture (prior 30 days), GU procedure (prior 30 days), antibiotic use (prior 14 days), hemodialysis, immunosuppression (active cancer or taking immunosuppressants or steroids in the prior 30 days), homelessness, previous enrollment, current incarceration, non-English speaking, trauma team activation, and lack of patient or proxy ability to give consent or respond to the survey |
| Association Between Social Isolation and Outpatient Follow-Up in Older Adults Following Emergency Department Discharge | Nia A. Cayenne, Gwen Costa Jacobsohn, PhD MA, Courtney M.C. Jones, PhD MPH, Eva H. DuGoff, PhD MPP, Amy L. Cochran, PhD, Thomas V. Caprio, MD MPH MS, Jeremy T. Cushman, MD MS, Rebecca K. Green, MPH, Amy J.H. Kind, MD PhDh, Michael Lohmeier, MD, Ranran Mi, MA, Manish N. Shah, MD MPH | 2021 | Archives of Gerontology and Geriatrics | Manuscript | Better understand the relationship between social  isolation and post-ED outpatient follow-up for older adults | Health equity/social EM | Primary enrollment | No, only ED | RCT; Cohort study | Yes | English | No | at least 60 years of age, residing in Dane County, WI or Monroe County, NY, community dwelling, had a primary care provider (PCP) affiliated with either health system, had a working telephone, and were discharged from the ED or ED observation unit to a community residence within 24 h of arrival | were previous participants, were actively enrolled in either hospice, a transitions program, or a care management program, did not have a permanent residence, presented with a primary behavioral or psychiatric health problem, were visually or hearing impaired, or had an ESI category of 1 |
| Trial Study Design to Test a Bilingual Digital Health Tool for Alcohol Use Disorders Among Latino Emergency Department Patients | Federico E. Vaca, James Dziura, Fuad Abujarad, Michael V. Pantalona, Allen Hsiao, Craig A. Field, Gail D'Onofrio | 2020 | Contemporary Clinical Trials | Manuscript | 1) To compare the efficacy of AB-CASI to Standard Care (SC) in the reduction of alcohol consumption in unhealthy (i.e., high-risk) drinkers [44]; 2) To compare the efficacy of ABCASI to SC in the reduction of alcohol-related negative health behaviors and consequences; 3) To compare the efficacy of AB-CASI to SC in 30-day treatment engagement | Health equity/social EM | Primary enrollment | No, only ED | RCT; Protocol | Yes | English; Spanish | No | adult ED patients | Current enrollment in alcohol or substance abuse treatment program, Current ED visit for acute psychosis (i.e. suicidal or homicidal ideation), Condition that precludes interview or AB-CASI use i.e., life threatening injury/illness including sexual assault, poor decisional capacity due to cognitive impairment, Police custody Inability to provide two contact numbers for follow-up |
| Post-Sexual Assault Cigarette Smoking: Findings from a Randomized Clinical Trial of a Video-Based Intervention | Kate Walsh, Amanda K. Gilmore, Julie A. Schumacher, Scott F. Coffey, Patricia A. Frazier, Linda Ledray, Ron Aciernoi, Kenneth J. Ruggiero, Dean G. Kilpatrick, Heidi S. Resnick | 2020 | Addictive Behaviors | Manuscript | Tested whether a brief video intervention delivered in the emergency department was effective at reducing smoking following sexual assault | Health equity/social EM | Primary enrollment | No, only ED | RCT | Yes | English | Yes | Girls and women age 15 years or older (N = 711) who were recent victims of SA (rape, suspected rape or attempted rape) and who participated in a SAMFE within 7 days of assault | Non-English speaking or presenting with serious injuries, psychological distress, acute intoxication |
| Targeting of UnControlled Hypertension in the Emergency Department (TOUCHED): Design of a randomized controlled trial | Heather M Prendergast, MD, MPH, MS, Renee Petzel-Gimbar, PharmD, Spyros Kitsiou, PhD, Marina Del Rios, MD, MSc, Brenda Lara, MA, Maya Jackson, MA, Sara Heinert, PhD, MPH, Barry L Carter, PharmD, Ramon A. Durazo-Arvizu, PhD, Martha Daviglus, MD | 2021 | Contemporary Clinical Trials | Manuscript | To assess if we can decrease the disparities in HTN control by providing a streamlined intervention to high  risk populations that may use the ED as their primary care | Health equity/social EM | Primary enrollment | No, only ED | RCT; Protocol | Yes | English; Spanish | No | at least two recorded BP readings between BP > 140/90 mm Hg and >180/110 mm Hg from the current ED visit prior to discharge, age 18-75 year | Unable to verbalize comprehension of study or impaired decision making, Lives outside the Chicago area or plans to move in the next year, Currently pregnant or expecting to be pregnant in the next year |
| The Design and Conduct of a Randomized Clinical Trial Comparing Emergency Department Initiation of Sublingual versus a 7-day Extended-Release Injection Formulation of Buprenorphine for Opioid Use Disorder: Project ED INNOVATION | Gail D‚ÄôOnofrio, Kathryn F. Hawk, Andrew A. Herring, Jeanmarie Perrone, Ethan Cowan, Ryan P. McCormack, James Dziura, R. Andrew Taylor, Edouard Coupet, E. Jennifer Edelman, Michael V. Pantalon, Patricia H. Owens, Shara H. Martel,  Patrick G. O'Connor, Paul Van Veldhuisen, Nicholas DeVogel, Kristen Huntley, Sean M. Murphy, Michelle R. Lofwall, Sharon L. Walsh, David A. Fiellin | 2021 | Contemporary Clinical Trials | Manuscript | Describes the protocol for a Type 1 Hybrid effec¬≠tiveness- implementation study [11] testing SL-BUP versus XR-BUP on the primary outcome of patient engagement in formal addiction treat¬≠ment at 7 days. | Other clinical study | Primary enrollment | No, only ED | RCT; Clinical trial; Protocol | Yes | English | No | 18 years or older, treated in the ED during study screening hours, DSM-5 criteria for moderate to severe OUD, have a COWS score of >8 and a point of care urine toxicology test positive for opioids | urine positive for methadone, a medical or psychiatric condition that requires hospitalization, are pregnant, actively suicidal or severely cognitively impaired, present from an extended care facility, require opioids for a pain condition without evidence of misuse, are a prisoner, have had an opioid (excluding BUP) administered in the ED prior to enrollment and COWS remain <8 during their stay; or enrolled in MOUD treatment within past 14 days |
| Optimizing Scalable, Technology-Supported Behavioral Interventions to Prevent Opioid Misuse Among Adolescents and Young Adults in the Emergency Department: A Randomized Controlled Trial Protocol | Erin E. Bonar, Kelley M. Kidwell, Amy S.B. Bohnert, Carrie A. Bourque, Patrick M. Carter, Sarah J. Clark, Meyer D. Glantz, Cheryl A. King, Eve D. Losman,  Sean Esteban McCabe, Meredith L. Philyaw-Kotov, Lisa A. Prosseri, Terri Voepel-Lewisf, Kai Zheng, Maureen A. Walton | 2021 | Contemporary Clinical Trials | Manuscript | Testing efficacy of early interventions to reduce escalation of opioid (prescription or illicit) misuse among at-risk adolescents and young adults | Health equity/social EM | Primary enrollment | No, only ED | RCT | Yes | English | Yes | age 16-30, English-speaking, 2- month prescription opioid use plus at least 1 other risk factor (defined as recent misuse of cannabis or illicit drugs, other prescription drug misuse, binge drinking, depression or suicidality), or 12-month opioid misuse (prescription or illicit [e.g., heroin, fentanyl]) | medically and cognitively able (e.g., conscious, not intubated) to pro¬≠vide consent/assent, chief complaint of acute sexual assault or acute suicidality, a current cancer diagnosis or currently receiving cancer treatment, pregnant women |
| Emergency department patients with untreated opioid use disorder: A comparison of those seeking versus not seeking referral to substance use treatment | Edouard Coupet Jr, Gail D'Onofrio, Marek Chawarski, E.J. Edelman, Patrick G. O'Connor, Patricia Owens, Shara Martel, David A. Fiellin, Ethan Cowan, Lynne Richardson, Kristen Huntley, Lauren K. Whiteside, Michael S. Lyons, Richard E. Rothman, Michael Pantalon, Kathryn Hawk | 2021 | Drug and Alcohol Dependence | Manuscript | Investigate the sociodemographic and clinical characteristics of ED patients with untreated opioid use disorder and the relationship of those characteristics with whether they were seeking a referral to substance use treatment at the time of their ED visit | Health equity/social EM | Secondary analysis | No, only ED | Cross sectional study | No | English | No | 1) were 18 years of age or older, 2) met the DSM-5 criteria for current moderate/severe OUD, 3) were to be discharged, 4) were not on probation or parole, 5) were not under arrest/in police custody/going to be incarcerated, 6) had a phone, 7) had a positive urine toxicology test for opioids (including methadone and buprenorphine), 8) understood English, and 9) consented to study procedures | 1) tested positive solely for fentanyl since there was no FDA-approved fentanyl point of care testing for clinical care at the time of this study, 2) had a medical or psychiatric condition requiring hospi¬≠talization during their index ED visit, 3) were cognitively impaired, 4) were actively suicidal, 5) required opioids for pain management, 6) were sent to the ED for medical evaluation from an extended care facility, or 7) were enrolled in formal OUD treatment within the past 30 days |
| Piloting a Brief Intervention Plus Mobile Boosters for Drug Use among Emerging Adults Receiving Emergency Department Care | Erin E. Bonar, Rebecca M. Cunningham, Emily C. Sweezea, Frederic C. Blow, Laura E. Drislane, Maureen A. Walton | 2021 | Drug and Alcohol Dependence | Manuscript | Developed and evaluated the feasibility and acceptability of an ED-initiated brief intervention combined with booster messaging as a clinician-  extender primarily focusing on drug use, with a secondary focus on condomless sex | Health equity/social EM | Primary enrollment | No, only ED | RCT | Yes | English | No | emerging adults (ages 18-25) with recent drug use and sexual risk behavior | hearing/visual impairment, ED presentation for acute sexual assault or suicidality, and medical conditions precluding informed consent (e.g., acute psychosis, unconsciousnes |
| Effect of cohabitating partners on the development of posttraumatic stress symptoms after emergency department visits for stroke and transient ischemic attack | Talea Cornelius, Jeffrey L. Birk, Lilly Derby, Julia Ellis, Donald Edmondson | 2021 | Social Science & Medicine | Manuscript | Whether having a cohabiting partner modified the association between patients' early perceptions of  threat (e.g., feeling vulnerable, helpless) and longer-term posttraumatic stress symptoms | Health equity/social EM | Secondary analysis | No, only ED | Cohort study | No | English; Spanish | No | at least 18 years of age, were evaluated for stroke/TIA in the ED | terminal non-cardiovascular illness (life expectancy of less than one year), severe mental illness requiring urgent psychiatric hospitalization or intervention, alcohol or substance abuse that would prevent the ability to complete the study protocol, severe stroke, or lack of availability for follow-up |
| Discordance Between Satisfaction and Health Literacy Among Spanish-Speaking Patients with Limited English-Proficiency Seeking Emergency Department Care | Seiichi Villalona, Heide Castaeda, Jason W. Wilson, Nancy Romero-Daza, Mery Yanez Yuncosa and Christian Jeannot | 2023 | Hispanic health care international | Manuscript | Understand satisfaction with care, perceptions of medical staff concern, awareness, and comprehension of medical care among Spanish-speaking patients with limited English-language proficiency | Health equity/social EM | Primary enrollment | No, only ED | Cross sectional study; Qualitative | No | Spanish | No | 18 years of age or older, indicate Spanish as their preferred language of communication, and did not have a triage ESI of 1 or 2 | any past psychiatric history or mental health conditions that would impede obtaining informed consent, were under the custody of law enforcement officials, or were presenting to the ED for evaluation of substance or alcohol use |
| Insights from the shadows: exploring deservingness of care in the emergency department and language as a social determinant of health | Seiichi Villalona | 2021 | BMJ | Manuscript | Addresses the existing gap in literature that ethnographically examines the experiences of Spanish- speaking patients with limited English proficiency in  clinical space | Health equity/social EM | Primary enrollment | No, only ED | Cross sectional study; Qualitative | No | Spanish | No | at least 18 years of age, identified Spanish as their preferred language of communication and were triaged as a non-urgent ED visit | presented to the ED under circumstances that would impede obtaining informed consent, such as being under the influence of alcohol or substances (pharmacological or illicit), having medical histories of psychiatric or mental health conditions, or if the patient was under the custody of law enforcement officials |
| Predicting Language Performance in Adults wth Mild Traumatic Brain Injury and Orthopedic Controls | Rocio Norman, Sandeep Subramanian, Meghan McDonald, Mackenzie Cross, Manish Shah, Lyn Turkstra | 2019 | University of Texas Health Late Breaking Research Posters | Abstract | To determine whether speech rate, cognitive performance and neurobehavioral symptoms can be utilized to predict discourse skills in adults with mild traumatic brain injury | Other clinical study | Primary enrollment | No, only ED | Cohort study | No | English | No | over 18 years old, diagnosed with an mild TBI or orthopedic injury by a medical provider in the ED | history of learning, speech-language and neurological disorder |
| Adolescent Attitudes Towards Sexually Transmitted Infection Screening in the Emergency Department | Addison S. Gearhart, MD, Gia M. Badolato, MPH, and Monika K. Goyal, MD, MSCE | 2020 | Pediatric Emergency Care | Manuscript | To assess adolescent attitudes toward ED-based STI screening | Health equity/social EM | Secondary analysis | No, only ED | Cross sectional study | No | English | Yes | ages of 14 and 21 years presenting to the ED with non STI-related complaints | critically ill, developmentally delayed, presented with altered mental status/psychiatric emergency, a victim of assault, or in police custody |
| Randomized Study of Metoclopramide Plus Diphenhydramine for Acute Posttraumatic Headache | Benjamin W. Friedman, MD, Eddie Irizarry, MD, Darnell Cain, MD, Arianna Caradonna, BA, Mia T. Minen, MD, Clemencia Solorzano, PharmD, Eleftheria Zias, RPH, David Zybert, FNP, Michael McGregor, MD, Polly E. Bijur, PhD, and E. John Gallagher, MD | 2021 | Neurology | Manuscript | To determine whether IV metoclopramide 20 mg + diphenhydramine 25 mg (M + D) was more efficacious than IV placebo for acute moderate or severe posttraumatic headache in the emergency room | Other clinical study | Primary enrollment | No, only ED | RCT | Yes | English; Spanish | No | adults (>18 years of age) who met International Classification of Headache Disorders criteria for acute posttraumatic headache | >10 days had elapsed since the head trauma, if the headache had already been treated with an antidopaminergic medication, or for investigational medication contraindications, known investigational medication allergies, or pregnancy |
| Stand Up and Fight Falls: Can a Video Intervention Help Reduce Falls in the Geriatric Population? | Margot Samson, Kathleen Davenport, Caroline Rizzo, Shan W. Liu | 2020 | Cureus | Manuscript | Examines if a video intervention presented in the ED to patients who have fallen could improve fall education and reduce future falls. | Other clinical study | Primary enrollment | No, only ED | Cohort study | Yes | English | No | fallen in the past two weeks (as noted in the chief complaint or ED triage note) and if they were 65 years or older | condition was too acute or unstable, who declined to consent, or who were cognitively impaired (as determined by the provider for that patient) |
| A pilot mixed-methods randomized controlled trial of verbal versus electronic screening for adverse social determinants of health | Wendy Macias-Konstantopoulos MD, MPH, Gia Ciccolo MPH, Alona Muzikansky MA, Margaret Samuels-Kalow MD, Mphil | 2022 | Journal of the American College of Emergency Physicians Open | Manuscript | Examining the optimal modality for screening for social risk in the emergency department | Health equity/social EM | Primary enrollment | No, only ED | RCT; Qualitative | Yes | English; Spanish | Yes | patients (or parents/legal guardians of pediatric-age patients) >18 years old and able to consent | patients unwilling to have an audio-recorded interview, medically and psychiatrically unstable patients, and emotionally distressed patients such as those presenting for sexual assault |
| Predicting Cardiovascular Risk Using Social Media Data: Performance Evaluation of Machine-Learning Models | Anietie U Andy PhD; Sharath C Guntuku, PhD; Srinath Adusumalli, MD, MSc; David A Asch, MD, MBA; Peter W Groeneveld, MD, MS; Lyle H Ungar, PhD; Raina M Merchant, MD, MSHP | 2021 | JMIR Cardio | Manuscript | Looked at patients' Facebook posts to provide insights about an individual‚Äôs ASCVD risk and inform approaches to risk modification | Other clinical study | Primary enrollment | Other: inpatient wards, outpatient clinics | Cross sectional study | No | Did not specify languages recruited | No | patients without a prior history of coronary heart disease, an ASCVD score in their EMR, and more than 200 words in their Facebook posts | unable to provide consent |
| Point-of-Care Ultrasonography in the Diagnosis of Retinal Detachment, Vitreous Hemorrhage, and Vitreous Detachment in the Emergency Department | Shadi Lahham, MD, MS; Inna Shniter, MD; Maxwell Thompson, MD; Dana Le, BS; Tushank Chadha, BS; Thomas Mailhot, MD; Tarina Lee Kang, MD; Alan Chiem, MD, MPH; Stephanie Tseeng, MD; John C. Fox, MD | 2019 | JAMA Network Open | Manuscript | To perform a large-scale, multicenter study to determine the utility of POCUS for diagnosing retinal detachment, vitreous hemorrhage, and vitreous detachment in the ED | Other clinical study | Primary enrollment | No, only ED | Cohort study | No | English; Spanish | No | presented to the ED with ocular symptoms; with a concern for RD, VH, or VD; and undergoing an ED ophthalmologic consultation | younger than 18 years, those who declined to be enrolled in the study, and those with ocular trauma or suspicion for globe rupture |
| Assessment of the Acceptability and Feasibility of Using Mobile Robotic Systems for Patient Evaluation | Peter R. Chai, MD, MMS; Farah Z. Dadabhoy, MD; Hen-Wei Huang, PhD; Jacqueline N. Chu, MD; Annie Feng, BS; Hien M. Le, BS; Joy Collins, MPH; Marco da Silva, PhD; Marc Raibert, PhD; Chin Hur, MD; Edward W. Boyer, MD, PhD; Giovanni Traverso, MB, BChir, PhD | 2021 | JAMA Network Open | Manuscript | Understand attitudes toward robotic system‚Äìfacilitated health care tasks, such as the facilitation of telehealth interviews and the acquisition of contactless vital signs and nasal and oral swabs, among a national sample of individuals in the US | Other clinical study | Primary enrollment | Other: national survey | Cohort study; Cross sectional study | No | English; Spanish; Portuguese ; French; Creole; Chinese (including Mandarin); Arabic; Vietnamese ; Korean; Russian; Other: For those who did not speak English, a certified hospital interpreter explained the study procedures and obtained informed consent | No | patients triaged in the novel tent space or the standard ED waiting room or who directly received a room in the ED | medically unstable and unable to participate in an interview |
| Patient Perceptions About Opioid Risk Communications Within the Context of a Randomized Clinical Trial | Abby R. Dolan, MPH; Erica B. Goldberg, MSW; Carolyn C. Cannuscio, ScD; Matthew P. Abrams, MA; Rachel Feuerstein-Simon, MPH, MPA; Xochitl Luna Marti, MPH; Jason Mazique, BA; Marilyn M. Schapira, MD, MPH; Zachary F. Meisel, MD, MPH, MSHP | 2022 | JAMA Network Open \| Emergency Medicine | Manuscript | Explore patient experiences related to decisions regarding analgesia after an emergency department visit | Other clinical study | Primary enrollment | No, only ED | RCT; Clinical trial | Yes | English | No | aged 18 to 70 years; capable of providing informed consent; having a chief complaint indicating acute neck or back pain, flank pain, or both; eligible for ED discharge within 24 hours of enrollment | no email or smartphone access |
| Intimate Partner Violence and Controlling Behaviors Experienced by Emergency Department Patients: Differences by Sexual Orientation and Gender Identification | Karisa K. Harland, MPH, PhD, Corinne Peek-Asa, MPH, PhD and Audrey F. Saftlas, MPH, PhD | 2021 | Journal of Interpersonal Violence | Manuscript | Estimate the prevalence of intimate partner violence in an emergency department by sexual orientation and gender identification | Health equity/social EM | Primary enrollment | No, only ED | Cross sectional study | No | English | No | 18 to 64 years of age and presented to the ED without visitors | unable to consent due to alcohol intoxication, had a developmental disability or a durable power of attorney |
| FEASIBILITY OF HOSPITAL-LEVEL CARE AT HOME FOR NON-SEVERE ACUTE PANCREATITIS | David X. Jin, Henry M. Mitchell, Nicole A. Rosario Nieves, Julia McNabb-Baltar, Peter A. Banks, David M. Levine | 2022 | American Gastroenterological Association Abstracts | Abstract | To evaluate the feasibility of home hospital care for patients with non-severe acute pancreatitis | Other clinical study | Primary enrollment | No, only ED | Cohort study | Yes | English; Spanish | No | All adult patients presenting directly to the ED with acute pancreatitis and who lived within a 7.5-mile radius | severe AP at presentation, cholangitis, imaging revealing new pancreatic necrosis/hypo-perfusion (if performed), and certain comorbidities |
| The feasibility of the vot-er voter registration model in a public hospital emergency department | Jennifer Lee, MD; Larissa Unruh, MD; Ameera Haamid, MD; Ashlea Winfield, MD; Errick Christian, MA; Rashid Kysia, MD, MPH; Pilar Guerrero, MD | 2022 | Western Journal of Emergency Medicine | Abstract | Evaluate the feasibility of a voter registration  system in a public hospital emergency department | Health equity/social EM | Primary enrollment | No, only ED | Cross sectional study | No | English; Spanish | No | >18 years old | English or spanish speaking |
| Part II: A Qualitative Study of Social Risk Screening Acceptability in Patients and Caregivers | Elena Byhoff, MD, MSc, Emilia H. De Marchis, MD, MAS, Danielle Hessler, PhD, Caroline Fichtenberg, PhD, Nancy Adler, PhD, Alicia J. Cohen, MD, MSc, Kelly M. Doran, MD, MHS, Stephanie Ettinger de Cuba, MPH, Eric W. Fleegler, MD, MPH, Nicholas Gavin, MD, Amy G. Huebschmann, MD, MSc, Stacy Tessler Lindau, MD, MAPP, Elizabeth L. Tung, MD, MS, Maria Raven, MD, MPH, MSc, Susan Jepson, MPH, BSN, Wendy Johnson, MD, MPH, Ardis L. Olson, MD, Megan Sandel, MD, MPH, Richard S. Sheward, MPP, Laura M. Gottlieb, MD, MPH | 2019 | American Journal of Preventative Medicine | Manuscript | Better understand patient and caregiver perspectives on social etter understand patient and caregiver perspectives on social risk screening across different healthcare settings. | Health equity/social EM | Primary enrollment | Other: PCP practices and pediatric EDs | Cross sectional study | No | English; Spanish | No | >18 (caregiver or patient) | not English or Spanish speaking |
| 95 Assessment of Social Determinants of Health and Linkage to Care Within the UMassMemorial Medical Center Emergency Department | Harward S, Rahman S, Taweh O, Avalone V, McMahon C, Modi P | 2021 | Annals of Emergency Medicine | Abstract | To characterize the prevalence of common social determinants of health among adult patients presenting to an ED | Health equity/social EM | Primary enrollment | No, only ED | Cross sectional study | No | English; Spanish; Portuguese ; French; Creole; Chinese (including Mandarin); Arabic; Vietnamese ; Korean; Russian; Other: verbally consented in their preferred language | No | patients with age under 18 yr, medical or cognitive inability to participate, or currently in state or federal custody | patients with age under 18 yr, medical or cognitive inability to participate, or currently in state or federal custody |
| Hand grip strength is not predictive of future fall risk | Christopher Solie, Morgan R. Bobb, Karisa K. Harland, Catherine Fairfield, and Nicholas M. | 2018 | Society for Academic Emergency Medicine | Abstract | To test the hypothesis that decreased hand grip strength is predictive of a future fall in older adults presenting to the emergency department | Other clinical study | Primary enrollment | No, only ED | Cohort study | No | English | No | 65 years and greater | current incarceration and living in a care facility, patients had hand grip strength measured in both hands while in the ED |
| Validation of Australian fall risk tool in American emergency department | Christopher Solie, Morgan R. Bobb, Karisa K. Harland, Catherine Fairfield, and Nicholas M. | 2018 | Society for Academic Emergency Medicine | Abstract | To perform a validation study of Tiedemann's screening tool in the United States | Other clinical study | Primary enrollment | No, only ED | Cohort study | No | English | No | 65 years and older | current incarceration and living in a care facility |
| Utility of focused cardiac ultrasound for pulmonary embolism in emergency department patients with abnormal vitals | James Daley, Kristin Dwyer, Zachary Grunwald, Daniel Shaw, Michael Vrablik, Kennedy Hall, Alexandra Schick, Andrew S.Liteplo, Nancy Hun, Rachel Liu, and Christopher Moore | 2019 | Society for Academic Emergency Medicine | Abstract | If in patients with tachycardia or hypotension FOCUS would be sensitive for excluding pulmonary embolism | Other clinical study | Primary enrollment | No, only ED | Cohort study | Yes | English | No | >18 years | poor FOCUS windows, non-English speaking |
| Patient Performed Thoracic Lung Ultrasound | McFadden K, Krauss B, Ma I, Vivian R, Gullikson J, Al Saud A, Huang C, Liteplo A, Shokoohi H | 2021 | Annals of Emergency Medicine | Abstract | Evaluate the ability of patients to self-perform lung ultrasound (LUS) with minimal guidance | Other clinical study | Primary enrollment | No, only ED | Non randomized experimental study | Yes | English | No | > 18 years old | unable to provide consent |
| Improving emergency department patient experience through implementation of an informational brochure | Rohit B. Sangal, Clinton Orloski, Frances S. Shofer, and Angela | 2018 | Society for Academic Emergency Medicine | Abstract | Evaluate the effect of an informational paper brochure explaining ED operations as a cost-effective tool to improve patient experience | Other clinical study | Primary enrollment | No, only ED | Cross sectional study; Other: interventional trial | Yes | English | Does not say | English speaking adults presenting to an urban, academic ED | unable to provide consent |
| Emergency department clinician and patient communications vary by race and gender | Chandni Pawar | 2020 | Society for Academic Emergency Medicine | Abstract | To see if ED clinicians are more likely to interrupt patients based on demographics. | Health equity/social EM | Primary enrollment | No, only ED | Cross sectional study | No | English | Does not say | English language encounter | unable to provide consent |
| Prehospital use of acute coronary syndrome and pulmonary embolism decision AIDS: Results from rescue study | Anna C. Snavely, Simon A. Mahler, Brian Hehl, Jordan Vorrie, Matthew Wells, R. Darrell Nelson, Nicklaus P. Ashburn, NellaW. Hendley, Chadwick D. Miller, and Jason P. | 2020 | Society for Academic Emergency Medicine | Abstract | To test the prehospital completion and inter-rater reliability of chest pain risk scores | Other clinical study | Primary enrollment | Other: prehospital | Cohort study | No | Did not specify languages recruited | No | >21 years with acute non-traumatic chest pain without ST elevation myocardial infarction or unstable vital signs | interfacility transports |
| Extended-Release Injectable Buprenorphine for Emergency Department Patients with Opioid Use Disorder in Minimal Withdrawal | D‚Onofrio G, Herring A, Perrone J, Hawk K, Samuels E, Cowan E, McCormack R, DeVogel N, Dziura J, Fiellin | 2021 | Annals of Emergency Medicine | Abstract | To assess the safety of a 7-day extended-release injectable buprenorphine formulation | Health equity/social EM | Primary enrollment | No, only ED | Case series | Yes | English | No | age 18 or older, point-of-care urine testing positive for opioids, not requiring hospitalization or use of opioids for pain | urine positive for methadone, active suicidality, or cognitive impairment with inability to consent |
| Temporal characteristics of homelessness and housing instability in an urban emergency department | Leah Fraimow-Wong, Jennifer Sun, Daniel Haro, Partow Imani, and Harrison J. | 2019 | Society for Academic Emergency Medicine | Abstract | The presence of housing specialists in the ED may significantly improve outcomes for homeless and unstably housed patients. Little is known about the true prevalence of housing instability in the ED and whether a greater number of such patients present to the ED at certain times of the day | Health equity/social EM | Primary enrollment | No, only ED | Cross sectional study | No | English; Spanish | No | Any patient 18 or older who completed an ED visit during the study time period | unable to provide consent |
| Correlating demographics, screening modalities, and health literacy in an urban emergency department | Sonali Gandhi, Jason Murphy, Katrece Outlaw, Toni Riveros, Errick Christian, Rosaura  Fernandez, and Lum | 2018 | Society for Academic Emergency Medicine | Abstract | Identify alternative screening questions from past studies to replace the lengthier ShortTest of Functional Health Literacy in Adults | Health equity/social EM | Primary enrollment | No, only ED | Cross sectional study | No | English; Spanish | Does not say | adult ED patients | Medically cleared patients were ineligible ifaltered for any reason, visually impaired, imprisoned, had major trauma, or admitted to an ICU |
| The impact of anti-immigrant presidential rhetoric on legal latinos presenting to the emergency department | Jesus R. Torres, Mayra Cruz, Breena R. Taira, Carolina Ornelas, Luis Lovato, and Robert | 2018 | Society for Academic Emergency Medicine | Abstract | To assess the impact of anti-immigrant presidential rhetoric on Latino Legal US Residents | Health equity/social EM | Primary enrollment | No, only ED | Cross sectional study | No | English; Spanish | No | convenience sample of adult patients | age<18 years, arrival by ambulance, incarceration, intoxication, altered mental status and psychiatric condition |
| The effects of presidential rhetoric on undocumented latinos presenting to the emergency department | Jesus R. Torres, Carolina Ornelas, Luis Lovato, Mayra Cruz, Breena R. Taira, and Robert | 2018 | Society for Academic Emergency Medicine | Abstract | To assess the effects of recent anti-immigrant presidential rhetoric on fear of deportation | Health equity/social EM | Primary enrollment | No, only ED | Cross sectional study | No | English; Spanish | No | convenience sample of adult patients | age<18 years, arrival by ambulance, incarceration, intoxication, altered mental status and psychiatric condition |
| Brief screening to assess food insecurity in the emergency department | Brock Chimileski, Zachary Boivin, Judith Oriental-Pierre, Sharon R. Smith | 2021 | Society for Academic Emergency Medicine | Abstract | Is food insecurity screening feasible in the ED | Health equity/social EM | Primary enrollment | No, only ED | Cross sectional study | No | English | Yes | non critically ill | non english speaking |
| Emergency department patient perceptions of quality improvement and role of patient activation and health literacy | Emily Zhao and Keith E. | 2019 | Society for Academic Emergency Medicine | Abstract | Assess the baseline knowledge and atitudes of emergency department (ED) patients related to  QI and test whether these were modified by underlying levels of health literacy and activation | Health equity/social EM | Primary enrollment | No, only ED | Cross sectional study | No | English | No | convenience sample of adult (>18 years old) patients | high acuity (triage score<2) |
| Perceived Barriers to Universal HIV Screening Among Emergency Department Patients: Identifying the Population and Targeting Areas for Improvement | John Coyne CJ, Simonsen E, Brennan J, Castillo EM, Vilke GM | 2019 | Annals of Emergency Medicine | Abstract | Define the characteristics of our ED population and to identify any perceived barriers to HIV testing among ED patients | Health equity/social EM | Primary enrollment | No, only ED | Cross sectional study | No | English | No | English-speaking ED patients 18 years of age or older | excluded patients who were in the custody of law enforcement, were critically ill, or were unable to consent for any other reason |
| Does a solely spanish-speaking patient population have discrepancies in the emergency department waiting times? | Ambar R. Martin Lopez, Katia Lugo-Enriquez, Leslie Camacho-Acevedo, Ademola Adewale, and Clara Mora-Montero | 2020 | Society for Academic Emergency Medicine | Abstract | To evaluate the existence of discrepancies in the throughput time of patients requiring English to Spanish  interpretation as compared to bi/multilingual patients with both English and Spanish proficiency | Health equity/social EM | Primary enrollment | No, only ED | Cross sectional study | No | English; Spanish | Does not say | discharged from the ED |  |
| Acceptability of a brief motivational interview-based intervention to empower older adults with serious illness to formulate their goals for future medical care in the emergency department | K. Ouchi, S. Block, N. George, L. Fellion, A. Reust, M. Hasdianda, S. Pajka, M. A. Schonberg, E. Bernstein, J. Tulsky | 2019 | Journal of American Geriatric Society 2018 Annual Meeting | Abstract | To understand the perspectives of older adults with serious illness who underwent a brief motivational interview-based intervention to empower older adults with serious illness to formulate their goals for medical care in the ED | Other clinical study | Primary enrollment | No, only ED | Qualitative | No | English | No | >65 years old with serious illness or who treating ED clinician "would not be surprised if died in the next 12 months" and has the capacity to consent | determined by the treating ED clinicians to be inappropriate for this study |
| Utilizing Existing Infrastructure to Rapidly Create a Cost-effective BioBank During the COVID-19 Pandemic in a Southern Community Hospital | Declan A, Moschella P | 2022 | Annals of Emergency Medicine | Abstract | Highlight the utilization of existing infrastructure through a Department of Oncology to facilitate the rapid and cost-effective creation of a COVID-19 Biobank | Other clinical study | Primary enrollment | No, only ED | Cohort study | Yes | English | No | patients who visited the PRISMA Health Eds |  |
| Intimate partner violence enacted via technology is higher among lesbian, gay, bisexual, transgender, queer emergency department population | Brooke A. Dugdale, and Karisa K. Harland | 2020 | Society for Academic Emergency Medicine | Abstract | Studied the prevalence and differences in IPV enacted via technology by sexual orientation | Health equity/social EM | Primary enrollment | No, only ED | Cross sectional study | No | English | No | 18-64 years old, and presenting to the ED without visitors (a human subjects' safety requirement) | unable to provide consent |
| Language access in the emergency department: The patient's perspective | Hemang A. Acharya, Alexis Aleman, Dannae Martin, Fernando Avila-Garibay, Emmanuel Cordova, Aristides Orue, Luis Lovato, and Breena R. Tair | 2020 | Society for Academic Emergency Medicine | Abstract | To assess ED patient knowledge of language access laws in a setting where most patients have limited English proficiency | Health equity/social EM | Primary enrollment | No, only ED | Cross sectional study | No | English; Spanish; Portuguese ; French; Creole; Chinese (including Mandarin); Arabic; Vietnamese ; Korean; Russian | Does not say |  |  |
| A Survey of Exposure to Community Violence and Adverse Childhood Experiences in Emergency Department Patients | Ansari S, Cachola L, Guevara Y | 2022 | Annals of Emergency Medicine | Abstract | 1) identify the prevalence of ECV and ACEs in an urban setting, 2) identify an association between geographic residence and ECV and ACEs, and 3) determine perceived social service needs for patients who endorse ACEs | Health equity/social EM | Primary enrollment | No, only ED | Cross sectional study | No | English | No | in the ED, 18 years and older, could read English, and consented to the study | they declined, were critically ill, could not read English, or were under 18 years old |
| Delirium keyword association with delirium status in older emergency department patients as measured by the confusion assessment method | L. Desrochers | 2019 | American Geriatrics Society Annual Meeting | Abstract | If ED medical record documentation of specific keywords as indicators of delirium by ED physicians was associated with delirium as measured by a gold standard | Other clinical study | Primary enrollment | No, only ED | Cross sectional study | No | English | No | >65 years | patient is in clinical decision making unit, severely hearing impaired, had a current stroke, TIA, delirium tremens, or head trauma diagnosis |
| Professional interpreter use among limited english proficiency patients in the emergency department | R Bitrus  K Rose A Robichaud  A Cruz | 2018 | Academic Emergency Medicine | Abstract | Assess the communication preferences of patients with limited English proficiency | Health equity/social EM | Primary enrollment | No, only ED | Cross sectional study | No | Spanish; Arabic | No | 18 years of age and older | LEP |
| Language discordance is associated with suboptimal patient-provider communication in the emergency department | David S. Edelman; Dana Palmer; Emily Romero; Bernard Chang; Ian Kronish | 2020 | Society of General Internal Medicine | Abstract | Language discordance would be most strongly associated with patient perceptions of poor patient-provider communication | Health equity/social EM | Secondary analysis | No, only ED | Cohort study | No | English; Spanish | No |  |  |
| Substance use and social determinants of health among emergency department patients | Evan Gerber, Donna Castelblanco, Neloufar Rahai, Ryan McCormack, Ian Wittman,  Donna Shelly, John Rotrosen, Lillian Gelberg, and Kelly | 2018 | Society for Academic Emergency Medicine | Abstract | Compared the prevalence of several SDOH among ED patients who did and did not  screen positive for unhealthy alcohol and drug use | Health equity/social EM | Primary enrollment | No, only ED | Cross sectional study | No | English; Spanish | No | >18 years old | medically/ psychiatrically unstable, in prison/police custody |
| Emergency department longitudinal integrated care: a pilot randomized clinical trial of a multi-component intervention for patients with opioid use | Lauren Whiteside, Ly Huynh, Sophie C. Morse, Caleb Banta-Green, William J. Meurer, Rebecca Cunningham, and Douglas | 2020 | Society for Academic Emergency Medicine | Abstract | To determine the feasibility and acceptability of "Emergency Department Longitudinal  Integrated Care with exploratory data on the main outcome of opioid use | Health equity/social EM | Primary enrollment | No, only ED | RCT | Yes | English | Does not say | at-risk for OUD based on NM-ASSIST >4 for illicit opioids or prescription opioid misuse, had a phone | suicidal, required resuscitation or on chronic opioids |
| Do emergency department patients with musculoskeletal pain prefer opioids for pain management? | Cooper R. Swenson, Henry C. Thode, and Adam J. | 2018 | Society for Academic Emergency Medicine | Abstract | Determined patient preferences for analgesic in the ED before and after a brief educational intervention informing them of the risks and benefits of opioids  versus non-steroidal anti-inflammatory drugs | Other clinical study | Primary enrollment | No, only ED | Cross sectional study | Yes | English | No | ages 18-65 years with acute (less than one week) musculoskeletal pain | lacking medical capacity; those with allergies to NSAID; pregnant patients; those with current opioid use |
| Vaginal self-sampling is not inferior to provider endocervical sampling for gonorrhea and chlamydia diagnosis | Brian Chinnock MD, Mackensie Yore MD, Jessica Mason MD, Mallory Kremer MD, Leyla Farshidpour, Diana Lopez, Jannet Castaneda | 2020 | Society for Academic Emergency Medicine | Manuscript | Assess if self-obtained vaginal swabs were noninferior to provider-performed endocervical sampling in the ED diagnosis of NG/ CT using a rapid NAAT | Other clinical study | Primary enrollment | No, only ED | Cohort study | Yes | English; Spanish | No | female patients >18 years old who were judged by the treating practitioner to need NG/CT testing | incarcerated, if an acute psychiatric condition precluded understanding instructions or giving informed consent, or treated for NG/CT within the previous 4 weeks |
| Knowledge of and barriers to learning cardiopulmonary resuscitation differ between English and Spanish speakers | Nanse Mendoza, Caleb Canders, Eva Gonzalez, and Breena R. | 2018 | Society for Academic Emergency Medicine | Abstract | To further define the role of language in the CPR utilization by comparing knowledge of and barriers to  learning CPR between English and Spanish speakers | Health equity/social EM | Primary enrollment | No, only ED | Cross sectional study | No | English; Spanish | No | adult patients | do not speak English or Spanish |
| Stand up, fight falls: Can a video intervention help reduce falls in the geriatric population? | Margot Samson, Katie Davenport, Caroline Rizzo, and Shan W. | 2020 | Society for Academic Emergency Medicine | Abstract | If a video intervention presented in the Emergency Department (ED) to patients who have fallen could improve fall education and reduce future falls | Other clinical study | Primary enrollment | No, only ED | Cohort study | Yes | English | No | 65 years and older who presented to a large academic ED for a fall | cognitively impaired, or whose condition was too acute (determined by providing physician) |
| The impact of presidential anti-immigrant rhetoric on latino patients presenting to the emergency department | Jesus R. Torres , Robert Rodriguez , Breena R. Taira, Luis M. Lovato , Carolina Ornelas, Mayra Cruz, Angela Wong, Jennifer Sun , and Harrison | 2019 | Society for Academic Emergency Medicine | Abstract | Conducted a follow-up study in the new anti-immigrant political climate on perceived threat of being identified as undocumented is an influencing factor for undocumented Latino immigrants | Health equity/social EM | Primary enrollment | No, only ED | Cross sectional study | No | English; Spanish | No | a convenience sample of adult subjects | minors, critically ill, incarcerated, intoxicated, altered mental status, psychiatric holds and those arriving by ambulance |
| Identifying eligibility for community-based services among older emergency department patients using malnutrition and food insecurity screeners: A prospective study | S. Bodepudi, A. Morris, S. Bowen J. Engelberg, B. Schmitthenner, T. F. Platts-Mills | 2019 | Journal of the American Geriatrics Society | Abstract | Assessed the ability of an ED-based screener that combines the Malnutrition Screening Tool + Hunger Vital Signs to identify older adults eligible for community-based services | Other clinical study | Primary enrollment | No, only ED | Cross sectional study | Yes | English | No | 60 years or older | critically ill patients with an ESI Level 1, altered mental status, skilled nursing residents |
| Comparing video and audio interpretation services in the modern emergency health care setting | Emily Au, Michael Chary, Sara Verma, Nameka Khan, Zuhair Ali, and Jonathan Siega | 2020 | Society for Academic Emergency Medicine | Abstract | Comparing time for audiovisual interpretation vs audio interpretation in the ED | Health equity/social EM | Primary enrollment | No, only ED | Cross sectional study | No | Spanish; Chinese (including Mandarin); Arabic; Korean; Other: Bengali | No | all patients 22 years or older | immediately life-threatening injuries, hearing impairment, unable to provide consent, declined to participate or asked for a family member to interpret instead |
| Misconceptions and poor understanding of domestic violence reporting | Robert M. Rodriguez, Clarissa Ferguson, Afomia Mesfin, Jennifer Sun, and Erik Sorem Anderson | 2020 | Society for Academic Emergency Medicine | Abstract | To assess emergency department patients' beliefs about reporting of DV | Health equity/social EM | Primary enrollment | No, only ED | Cross sectional study | No | English; Spanish | No | adult ED patients | critical illness, psychiatric hold, incarceration, and chiefcomplaint of DV |
| Use of rideshare services for patient transport to the emergency department | Chiemeke Nwabueze, Aizad Dasti, Barbara Stahlman, Brent Becker | 2021 | Society for Academic Emergency Medicine | Abstract | To investigate the use of rideshare by patients accessing emergency medical care at our ED | Other clinical study | Primary enrollment | No, only ED | Cross sectional study | No | English; Spanish | Does not say | arrived via private vehicle or ambulance or declined to participate | Patients were excluded if they arrived via private vehicle or ambulance, were non-English/Spanish fluent, or declined to participate |
| Using linear probe point-of-care ultrasound to identify early intrauterine pregnancies in an emergency department | Shadi Lahham, Soheil Saadat, Michelle Nguyen, Isabelle Nepomuceno, Erinna Thai,  John | 2021 | Society for Academic Emergency Medicine | Abstract | Deter-mine whether a linear probe alone can identify first trimester IUP | Other clinical study | Primary enrollment | No, only ED | Cross sectional study | No | English; Spanish | No | adult women (18 years and older) in their first trimester of pregnancy (<12 weeks pregnant) with a BMI of <35 | psychiatric, incarcerated, or cognitively impaired patients |
| Patient-endorsed suicidality in emergency department settings: A comparison of frequent vs nonfrequent utilizers | Gloria Essien, Celine Larkin, April Sawko, Joe Davenport, Rachel Davis-Martin, Bo Wang,  Edwin D. Boudreaux | 2021 | Society for Academic Emergency Medicine | Abstract | Compare patient-endorsed suicidality between frequent and non-frequent utilizers in ED settings within a nine-month enrollment period | Health equity/social EM | Secondary analysis | No, only ED | Cohort study | No | English | Does not say |  |  |
